# Synthesis and Antiproliferative Activity of Triazoles Based on 2-Azabicycloalkanes

**DOI:** 10.3390/ma14082039

**Published:** 2021-04-18

**Authors:** Franz Steppeler, Dagmara Kłopotowska, Joanna Wietrzyk, Elżbieta Wojaczyńska

**Affiliations:** 1 Faculty of Chemistry, Wrocław University of Science and Technology, Wybrzeże Wyspiańskiego 27, 50-370 Wrocław, Poland; franz.steppeler@pwr.edu.pl; 2 Hirszfeld Institute of Immunology and Experimental Therapy, Polish Academy of Sciences, ul. Rudolfa Weigla 12, 53-114 Wrocław, Poland; dagmara.klopotowska@hirszfeld.pl (D.K.); joanna.wietrzyk@hirszfeld.pl (J.W.)

**Keywords:** 2-azabicycloalkanes, antiproliferative activity, cancer, CuAAC, triazoles

## Abstract

A library of 21 novel chiral 1,2,3-triazole-based 2-azabicycloalkane conjugates was designed and synthesized using the copper(I)-catalyzed click reaction. The obtained hybrids were assessed for their antiproliferative potency against three selected human cancer cell lines: Hs294T (melanoma), MIA PaCa-2 (pancreas tumor) and NCI-H1581 (lung tumor). The majority of the synthesized compounds demonstrated moderate to potent activity, and some of them appeared more selective than cisplatin, with selectivity index exceeding 9.

## 1. Introduction

According to WHO, malignant tumors are one of the leading causes of death globally (9.6 million cases in 2018), and it is predicted to remain a major cause of mortality in the next decades [1]. Although numerous anticancer agents have been developed, their use is connected with certain drawbacks, including side effects resulting from low selectivity and drug resistance. Thus, there is a continuous need for the design of new, efficient, and selective chemotherapeutics.

**Figure 1:**
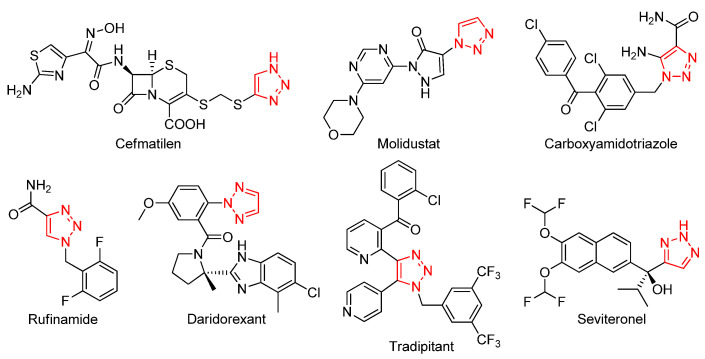
Selected biologically active compounds bearing triazole moieties.

Nitrogen-containing heterocycles have been recognized as important and efficient pharmacophores [2,3]. Among them, 1,2,3-triazoles exhibit both chemical stability and a unique ability to participate in various interactions with ions and neutral molecules, including biological targets. Not surprisingly, among numerous applications of 1,2,3-triazole as a building block in various fields, its use in the construction of biologically active structures has been explored [4,5,6]. For example, derivatives of this heterocycle have been used or tested as antibiotics (cefmatilen, cefatrizine, radezolid, solithromycin), anticonvulsants (rufinamide), drugs for the treatment of insomnia (daridorexant, suvorexant), anemia (molidustat), and atopic dermatitis (tradipitant), to name a few (Figure 1). Some of these compounds have been under clinical trials as anticancer agents, including carboxyamidotriazole and seviteronel [7,8] and other monomeric, but also dimeric, triazole derivatives were reported to exhibit significant antiproliferative activity [9,10,11].

The majority of the above-mentioned compounds contain also other pharmacophores. Such a combination of 1,2,3-triazole framework with other anticancer moieties may result in a development of new promising agents with the potential to overcome the drug resistance problem [12,13,14,15].

In our research, we have focused on the study of 2-azabicycloalkanes as attractive chiral scaffolds for the development of new ligands and catalysts for asymmetric synthesis but also as compounds exhibiting interesting biological activity [16]. Polyamine and sulfonamide derivatives of 2-azabicyclo[2.2.1]heptane and 2-azabicyclo-[3.2.1]octane were found to inhibit proliferation of selected cancer lines [16,17]. In this communication, a study on the synthesis of new hybrids incorporating 2-azabicycloalkane moiety and 1,2,3-triazole is described. Monomeric, dimeric, and trimeric derivatives were prepared from available starting materials according to convenient synthetic procedures [18]. Their action against selected cancer cell lines was evaluated in order to investigate if the combination of these heterocyclic units could generate active compounds. We have chosen melanoma cell line Hs294T, pancreas tumor cell line MIA PaCa-2 and lung tumor cell line NCI-H1581 which is particularly resistant to chemotherapy in humans. We believed that the attachment of 1,2,3-triazole to the 2-azabicycloalkane backbone could serve as a promising strategy to prepare novel drug candidates which could be efficient in the treatment of both drug-sensitive and drug-resistant cancers due to their independent mechanisms of action.

## 2. Materials and Methods

### 2.1. Chemistry

#### 2.1.1. General Considerations

All solvents and reagents were acquired from Merck KGaA, Darmstadt, Germany, and were used without any further purification. For the chromatographic separation silica gel 60 (70–230 mesh) was used, and thin layer chromatography (TLC) was carried out on silica gel 60 precoated plates. The TLC plates were visualized with UV light and by staining with iodine. The NMR spectra were recorded on Jeol 400yh (Jeol Ltd., Tokyo, Japan) and Bruker Avance II 600 instruments (Bruker, Billerica, MA, USA). ^1^H-NMR spectra were acquired at 400 and 600 MHz, respectively, whilst ^13^C-NMR spectra were acquired at 100 and 150 MHz, respectively. The measurements were conducted at 298 K, if not otherwise stated. The spectra were calibrated using residual solvent signals. All NMR spectra of new compounds are shown in the supplementary materials. Optical rotations were measured using an Optical Activity Ltd. Model AA-5 automatic polarimeter (Optical Activity, Ltd., Ramsey, UK). Infrared spectra in the range of 500–4000 cm^−1^ were recorded using a Perkin Elmer 2000 FTIR spectrophotometer (PerkinElmer, Waltham, MA, USA).High resolution mass spectra (HRMS) were collected using a Waters LCT Premier XE TOF instrument (Waters Corporation, Milford, MA, USA) with electrospray ionization. Melting points were determined on the Schmelzpunkt Bestimmer Apotec apparatus (WEPA Apothekenbedarf GmbH & Co. KG., Hillscheid, Germany) using standard open capillary.

#### 2.1.2. General Procedure of Triazole Synthesis

The synthesis of the non-commercial starting materials (1*S*,4*S*,5*R*)- and (1*S*,4*R*,5*R*)-2- [(*S*)-1-Phenylethyl]-4-azide-2-azabicyclo[3.2.1]octane was previously reported by us [19].

To synthesize the triazoles the following general procedure was conducted [18]: to a suspended mixture of (1*S*,4*S*,5*R*)-2-[(*S*)-1-Phenylethyl]-4-azide-2-azabicyclo[3.2.1]octane (256 mg, 1 mmol, 1 eq.) and appropriate alkynes with equivalent amount of alkyne moieties to the azide (mono: 1 eq., di: 0.5 eq., tri: 0.33 eq.) in t-butanol (2 mL) and water (1 mL) subsequently pyridine (0.2 mL), CuSO_4_·5H_2_O (51 mg, 0.2 mmol, 0.2 eq.), sodium ascorbate (100 mg, 0.5 mmol, 0.5 eq.), and potassium carbonate (112 mg, 0.8 mmol, 0.8 eq.) were added. The mixture was stirred for at least a day until the addition of dichloromethane (5 mL) and aq. NH_3_ (25%, 0.5 mL). After stirring overnight, sodium sulfide nonahydrate (sat. aq., 0.2 mL) was added to precipitate the copper. The reaction mixture was filtered through a pad of silica gel, washed with 50 mL of a chloroform methanol mixture (9:1 *v*/*v*), and the solvent was evaporated. The crude product was purified using liquid chromatography on silica gel using a 19:1 up to 14:1 chloroform methanol eluent yielding in products with various colors and either solid or liquid state of aggregation. The crystalline compounds were recrystallized out of MeOH and checked for their melting point, revealing broad ranges of transitions, a first transition to a semisolid state and a second nonreversible transition. (1*S*,4*R*,5*R*)-2-[(*S*)-1-Phenylethyl]-4-azide-2-azabicyclo[3.2.1]octane products were prepared in a similar manner. Syntheses of compounds **20** and **21** have been formerly published, and a deeper structural analysis was conducted [20].

#### 2.1.3. Experimental Data

**(1-((1*S*,4*S*,5*R*)-2-((*S*)-1-phenylethyl)-2-azabicyclo[3.2.1]octan-4-yl)-1H-1,2,3-triazol-4-yl)methanol (1)**


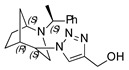


Yellow oil. Yield 280 mg (86%). ${\left[\alpha \right]}_{D}^{25}$ = +108.8 (c 0.68, CH_2_Cl_2_). ^1^H-NMR (400 MHz. CDCl_3_): δ 1.31–1.35 (m, 1H), 1.42 (d, 3H, *J* = 6.3 Hz), 1.49–1.54 (m, 1H), 1.57–1.66 (m, 2H), 1.85–1.90 (m, 2H), 2.61 (m, 1H), 2.73 (m, 1H), 3.10 (br, 1H), 3.39 (s, 1H), 3.79 (s, 1H), 4.44 (s, 1H), 4.79 (s, 2H), 7.22–7.26 (m, 1H), 7.32–7.37 (m, 4H), 8.02 (s, 1H) ppm. ^13^C-NMR (100 MHz, CDCl_3_): δ 21.0, 21.6, 27.2, 33.7, 40.3, 47.7, 56.2, 56.5, 59.7, 63.0, 121.9, 127.3, 127.4, 128.6, 146.4 ppm. IR (KBr): 549, 704, 779, 1058, 1212, 1347, 1453, 1492, 1600, 1667, 2868, 2925, 3061, 3435 cm^−1^. HRMS (ESI-TOF): *m/z* [M + H]^+^ calcd for (C_18_H_25_N_4_O_2_)^+^ 313.2029; found 313.2023. Elementary analysis: calcd C 69.20, H 7.74, N 17.93; found C 68.97, H 7.72, N 17.69.

**(1-((1*S*,4*R*,5*R*)-2-((*S*)-1-phenylethyl)-2-azabicyclo[3.2.1]octan-4-yl)-1H-1,2,3-triazol-4-yl)methanol (2)**


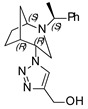


Yellow crystals. Yield 182 mg (58%). ${\left[\alpha \right]}_{D}^{25}$ = +28.6 (c 0.21, CH_2_Cl_2_). ^1^H-NMR (400 MHz, CDCl_3_): δ 1.05–1.11 (m, 1H), 1.31 (d, 3H, *J* = 6.7 Hz), 1.42–1.90 (m, 5H), 2.65 (dd, 1H, *J* = 9.7, 4.3 Hz), 2.77 (br, 1H), 2.81 (dd, 1H, *J* = 13.2, 4.7 Hz), 3.15 (t, 1H, *J* = 4.9 Hz), 3.26 (d, 1H, *J* = 13.1 Hz), 3.46 (q, 1H, *J* = 6.7 Hz), 4.61 (t, 1H, *J* = 4.2 Hz), 4.85 (d, 2H, *J* = 5.1 Hz), 7.23–7.29 (m, 1H), 7.30–7.36 (m, 4H), 8.32 (s, 1H) ppm. ^13^C-NMR (100 MHz, CDCl_3_): δ 21.7, 21.8, 27.1, 33.4, 40.4, 46.5, 56.7, 57.4, 59.9, 62.1, 122.0, 127.2, 127.4, 128.7, 145.2, 146,5 ppm. IR (KBr): 550, 702, 766, 953, 1019, 1054, 1119, 1224, 1343, 1452, 1492, 1722, 2868, 2968, 3144 cm^−1^. HRMS (ESI-TOF): *m/z* [M + H]^+^ calcd for (C_18_H_25_N_4_O)^+^ 313.2029; found 313.2021. Elementary analysis: calcd C 69.20, H 7.74, N 17.93; found C 69.07, H 8.01, N 17.61.

**(1-((1*S*,4*S*,5*R*)-2-((*S*)-1-phenylethyl)-2-azabicyclo[3.2.1]octan-4-yl)-1H-1,2,3-triazol-4-yl)methanamine (3)**


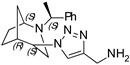


Orange oil. Yield 124 mg (39%). ${\left[\alpha \right]}_{D}^{25}$ = +128.0 (c 0.25, CH_2_Cl_2_). ^1^H-NMR (400 MHz, CDCl_3_): δ 1.25–1.31 (m, 1H), 1.37 (d, 3H, *J* = 6.6 Hz), 1.40–1.56 (m, 2H), 1.61 (d, 1H, *J* = 12.2), 1.78–1.88 (m, 2H), 2.44 (br., 2H), 2.55 (dd, 1H, *J* = 13.7, 4.7 Hz), 2.65 (d, 2H, *J* = 14.2 Hz), 3.33 (q, 1H, *J* = 6.6 Hz), 3.74 (t, 1H, *J* = 4.9 Hz), 3.99 (m, 2H), 4.38 (t, 1H, *J* = 4.3 Hz), 7.19–7.24 (m, 1H), 7.27–7.33 (m, 4H), 7.91 (s, 1H) ppm. ^13^C-NMR (100 MHz, CDCl_3_): δ 21.1, 21.6, 25.4, 27.2, 33.8, 40.4, 47.9, 56.2, 59.7, 63.0, 121.0, 127.4, 127.4, 128.6, 128.7, 144.8 ppm. IR (film): 549, 704, 779, 956, 1046, 1133, 1212, 1346, 1452, 1492, 1658, 2824, 2868, 2933, 3148 cm^−1^. HRMS (ESI-TOF): *m/z* [M + H]^+^ calcd for (C_18_H_26_N_5_)^+^ 312.2188; found 312.2190. Elementary analysis: calcd C 69.42, H 8.09, N 22.49; found C 69.56, H 8.17, N 22.10.

**(1-((1*S*,4*S*,5*R*)-2-((*S*)-1-phenylethyl)-2-azabicyclo[3.2.1]octan-4-yl)-1H-1,2,3-triazol-4-yl)methyl acrylate (4)**


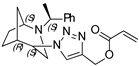


Orange crystals. Yield 250 mg (68%). ${\left[\alpha \right]}_{D}^{25}$ = +114.3 (c 0.51, CH_2_Cl_2_). ^1^H-NMR (600 MHz, CDCl_3_): δ 1.28–1.32 (m, 1H), 1.37 (d, 3H, *J* = 6.6 Hz), 1.43–1.49 (m, 1H), 1.52–1.56 (m, 1H), 1.58 (d, 1H, *J* = 12.5 Hz), 1.81–1.88 (m, 2H), 2.53 (dd, 1H, *J* = 4.7, 13.7 Hz), 2.63 (d, 1H, *J* = 13.7), 2.68 (dd, 1H, *J* = 4.5, 9.9 Hz), 3.32 (q, 1H, *J* = 6.6 Hz), 3.75 (t, 1H, *J* = 4.9 Hz), 4.41 (t, 1H, *J* = 4.2 Hz), 5.24 (d, 1H, *J* = 12.6 Hz), 5.35 (d, 1H, *J* = 12.6 Hz), 5.86 (dd, 1H, *J* = 1.4, 10.5 Hz), 6.16 (dd, 1H, *J* = 10.5, 17.4 Hz), 6.46 (dd, 1H, *J* = 1.4, 17.4 Hz), 7.19 (m, 1H), 7.28–7.31 (m, 4H), 8.10 (s, 1H) ppm. ^13^C-NMR (150 MHz, CDCl_3_): δ 21.2, 21.5, 27.3, 33.8, 40.3, 47.9, 56.1, 58.1, 59.8, 63.0, 124.1, 127.4, 127.4, 128.3, 128.8, 131.4, 141.6, 144.8, 166.0 ppm. IR (KBr): 546, 708, 773, 815, 925, 982, 1049, 1058, 1097, 1132, 1182, 1292, 1337, 1376, 1413, 1453, 1492, 1629, 1726, 1973, 2830, 2959, 3148 cm^−1^. HRMS (ESI-TOF): *m/z* [M + H]^+^ calcd for (C_21_H_27_N_4_O_2_)^+^ 367.2134; found 367.2130. Elementary analysis: calcd C 68.83, H 7.15, N 15.29; found C 68.71, H 7.17, N 15.14.

**(1-((1*S*,4*R*,5*R*)-2-((*S*)-1-phenylethyl)-2-azabicyclo[3.2.1]octan-4-yl)-1H-1,2,3-triazol-4-yl)methyl acrylate (5)**


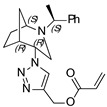


Yellow crystals. Yield 239 mg (62%). ${\left[\alpha \right]}_{D}^{25}$ = +12.1 (c 0.52, CH_2_Cl_2_). ^1^H-NMR (600 MHz, CDCl_3_): δ 1.05–1.10 (m, 1H), 1.31 (d, 3H, *J* = 6.6 Hz), 1.46–167 (m, 3H), 1.75–1.90 (m, 2H), 2.67 (dd, 1H, *J* = 9.8, 4.3 Hz), 2.82 (dd, 1H, *J* = 13.2, 4.6 Hz), 3.15 (t, 1H, *J* = 4.9 Hz), 3.25 (d, 1H, *J* = 13.1 Hz), 3.47 (q, 1H, *J* = 6.7 Hz), 4.61 (t, 1H, 4.1 Hz), 5.36 (d, 2H, 1.8 Hz), 5.86 (dd, 1H, *J* = 10.4, 1.4 Hz), 6.17 (dd, 1H, *J* = 17.3, 10.4 Hz), 6.47 (dd, 1H, *J* = 17.3, 1.4 Hz), 7.23–7.29 (m, 1H), 7.31–7.36 (m, 4H), 8.42 (s, 1H) ppm. ^13^C-NMR (150 MHz, CDCl_3_): δ 21.7, 21.9, 27.1, 33.4, 40.3, 46.4, 57.5, 58.1, 59.9, 62.1, 124.0, 127.2, 127.4, 128.3, 128.7, 131.4, 141.7, 145.2, 166.1 ppm. IR (KBr): 550, 702, 770, 954, 1047, 1183, 1268, 1407, 1452, 1492, 1634, 1726, 2869, 2964, 3148 cm^−1^. HRMS (ESI-TOF): *m/z* [M + H]^+^ calcd for (C_21_H_27_N_4_O_2_)^+^ 367.2134; found 367.2125. Elementary analysis: calcd C 68.83, H 7.15, N 15.29; found C 68.47, H 7.37, N 15.21.

**3-(1-((1*S*,4*S*,5*R*)-2-((*S*)-1-phenylethyl)-2-azabicyclo[3.2.1]octan-4-yl)-1H-1,2,3-triazol-4-yl)aniline (6)**


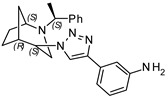


Brownish crystals. Yield 300 mg (78%). ${\left[\alpha \right]}_{D}^{25}$ = +247.5 (c 0.56, CH_2_Cl_2_). ^1^H-NMR (400 MHz, CDCl_3_): δ 1.29–1.34 (m, 1H), 1.40 (d, 3H, *J* = 6.7 Hz), 1.45–1.60 (m, 2H), 1.63 (d, 2H, *J* = 12.0 Hz), 1.82–1.91 (m, 2H), 2.60 (dd, 1H, *J* = 4.6, 13.7 Hz), 2.72 (d, 1H, *J* = 13.8 Hz), 2.78 (dd, 1H, *J* = 4.5, 9.7 Hz), 3.36 (q, 1H, *J* = 6.6 Hz), 3.78 (t, 1H, *J* = 4.8 Hz), 4.43 (t, 1H, *J* = 4.1 Hz), 6.65 (ddd, 1H, *J* = 1.0, 2.4, 7.9 Hz), 7.12 (m, 1H), 7.21–7.29 (m, 4H), 7.32–7.39 (m, 4H), 8.17 (s, 1H) ppm. ^13^C-NMR (100 MHz, CDCl_3_): δ 21.2, 21.7, 27.3, 33.8, 40.4, 48.1, 56.1, 59.7, 63.0, 112.3, 114.7, 116.0, 120.3, 127.4, 127.6, 128.7, 129.8, 132.3, 146.6, 147.0, 150.0 ppm. IR (KBr): 548, 703, 780, 874, 956, 1057, 1132, 1225, 1280, 1349, 1363, 1452, 1491, 1590, 1613, 2820, 2934, 3142, 3223, 3351 cm^−1^. HRMS (ESI-TOF): *m/z* [M + H]^+^ calcd for (C_23_H_28_N_5_)^+^ 374.2345; found 374.2344. Elementary analysis: calcd C 73.96, H 7.29, N 18.75; found C 73.75, H 7.47, N 18.43.

**3-(1-((1*S*,4*R*,5*R*)-2-((*S*)-1-phenylethyl)-2-azabicyclo[3.2.1]octan-4-yl)-1H-1,2,3-triazol-4-yl)aniline (7)**


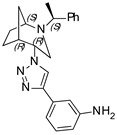


Brownish crystals. Yield 283 mg (76%). ${\left[\alpha \right]}_{D}^{25}$ = +102.4 (c 0.42, CH_2_Cl_2_). ^1^H-NMR (400 MHz, CDCl_3_): δ 1.06–1.14 (m, 1H), 1.35 (d, 3H, *J* = 6.6 Hz), 1.54–1.62 (m, 2H), 1.76–1.93 (m, 2H), 2.72 (dd, 1H, *J* = 9.5, 4.3 Hz), 2.86 (dd, 1H, *J* = 13.2, 4.5 Hz), 3.2 (t, 1H, *J* = 4.7 Hz), 3.29 (d, 1H, *J* = 13.1 Hz), 3.53 (q, 1H, *J* = 6.6 Hz), 4.64 (t, 1H, *J* = 3.8 Hz), 6.67 (ddd, 1H, *J* = 7.7, 2.4, 1.3 Hz), 7.16–7.24 (m, 2H), 7.25–7.31 (m, 4H), 7.33–7.37 (m, 4H), 7.66 (tt, 1H, *J* = 7.7, 1.7 Hz), 8.50 (s, 1H), 8.61 (s, 1H) ppm. ^13^C-NMR (100 MHz, CDCl_3_): δ 21.4, 22.1, 27.2, 33.5, 40.4, 46.3, 57.8, 59.8, 62.2, 112.4, 114.8, 116.2, 120.0, 127.2, 127.4, 128.7, 129.8, 132.2, 136.0, 147.0, 149.9 ppm. IR (KBr): 547, 702, 791, 874, 1055, 1138, 1224, 1281, 1351, 1447, 1484, 1591, 1610 2822, 2958, 3138, 3222, 3351 cm^−1^. HRMS (ESI-TOF): *m/z* [M + H]^+^ calcd for (C_23_H_28_N_5_)^+^ 374.2345; found 374.2343. Elementary analysis: calcd C 73.96, H 7.29, N 18.75; found C 74.15, H 7.17, N 18.40.

**(1*S*,4*S*,5*R*)-4-(4-((benzyloxy)methyl)-1H-1,2,3-triazol-1-yl)-2-((*S*)-1-phenylethyl)-2-azabicyclo[3.2.1]octane (8)**


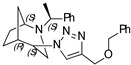


Orange oil. Yield 346 mg (79%). ${\left[\alpha \right]}_{D}^{25}$ = +83.6 (c 0.55, CH_2_Cl_2_). ^1^H-NMR (400 MHz, CDCl_3_): δ 1.27–1.33 (m, 2H), 1.37 (d, 3H, *J* = 6.6 Hz), 1.42–1.58 (m, 2H), 1.61 (d, 1H, *J* = 11.7 Hz), 1.80–1.89 (m, 2H), 2.56 (dd, 1H, *J* = 13.7, 4.7 Hz), 2.67 (d, 1H, *J* = 13.8 Hz), 2.72 (dd, 1H, *J* = 9.9, 4.5 Hz), 3.33 (q, 1H, *J* = 6.6 Hz), 3.75 (t, 1H, *J* = 4.9 Hz), 4.41 (t, 1H, *J* = 4.3 Hz), 4.60 (s, 2H), 4.69 (d, 2H, *J* = 3.1 Hz), 7.16–7.24 (m, 2H), 7.25–7.41 (m, 8H), 8.01 (s, 1H) ppm. ^13^C-NMR (100 MHz, CDCl_3_): δ 21.2, 21.6, 27.2, 33.8, 40.4, 47.9, 56.1, 59.7, 63.0, 64.0, 72.4, 122.8, 127.3, 127.4, 127.8, 128.0, 128.5, 128.7, 138.2, 143.9, 144.9 ppm. IR (film): 549, 702, 737, 778, 1046, 1094, 1347, 1453, 1492, 1600, 1684, 2097, 2866, 2953, 3147 cm^−1^. HRMS (ESI-TOF): *m/z* [M + H]^+^ calcd for (C_25_H_31_N_4_O_2_)^+^ 403.2498; found 403.2490. Elementary analysis: calcd C 74.59, H 7.51, N 13.92; found C 74.26, H 7.80, N 14.14.

**(1*S*,4*R*,5*R*)-4-(4-((benzyloxy)methyl)-1H-1,2,3-triazol-1-yl)-2-((*S*)-1-phenylethyl)-2-azabicyclo[3.2.1]octane (9)**


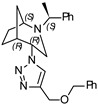


Orange oil. Yield 231 mg (56%). ${\left[\alpha \right]}_{D}^{25}$ = +25.1 (c 0.47, CH_2_Cl_2_). ^1^H-NMR (400 MHz, CDCl_3_): δ 1.05–1.11 (m, 1H), 1.24–1.37 (m, 1H), 1.31 (d, 3H, *J* = 6.6 Hz), 1.47–1.60 (m, 2H), 1.73–1.90 (m, 2H), 2.67 (dd, 1H, *J* = 9.8, 4.3 Hz), 2.81 (dd, 1H, *J* = 13.1, 4.7 Hz), 3.15 (t, 1H, *J* = 4.6 Hz), 3.26 (d, 1H, *J* = 13.2 Hz), 3.46 (q, 1H, 6.7 Hz), 4.62 (m, 1H), 4.65 (s, 2H), 4.75 (s, 2H), 7.22–7.42 (m, 10H), 8.35 (s, 1H) ppm. ^13^C-NMR (100 MHz, CDCl_3_): δ 21.8, 21.9, 27.1, 33.5, 40.4, 46.5, 57.5, 59.9, 62.1, 63.9, 72.4, 122.9, 127.2, 127.4, 127.8, 128.0, 128.6, 128.7, 138.1, 144.0, 145.3 ppm. IR (film): 551, 700, 737, 953, 1046, 1098, 1226, 1343, 1452, 1493, 1600, 1720, 2866, 1968, 3141 cm^−1^. HRMS (ESI-TOF): *m/z* [M + H]^+^ calcd for (C_25_H_31_N_4_O)^+^ 403.2420; found 403.2454. Elementary analysis: calcd C 74.59, H 7.51, N 13.92; found C 74.32, H 7.57, N 14.33.

**1,3-Bis-(1-((1*S*,4*S*,5*R*)-2-((*S*)-1-phenylethyl)-2-azabicyclo[3.2.1]octan-4-yl)-1H-1,2,3-triazol-4-yl) propane (10)**


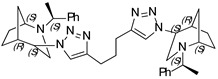


Off-white crystals. Yield 284 mg (92%). ${\left[\alpha \right]}_{D}^{25}$ = +121.1 (c 0.83, CH_2_Cl_2_). ^1^ H-NMR (400 MHz, CDCl_3_): δ 1.30 (m, 2H), 1.37 (d, 6H, *J* = 6.6 Hz), 1.41–1.59 (m, 4H), 1.64 (d, 2H, *J* = 12.2 Hz), 1.78–1.88 (m, 4H), 2.06 (qi, 2H, *J* = 7.7 Hz), 2.55 (dd, 2H, *J* = 13.6, 4.7 Hz), 2.66 (d, 2H, *J* = 13.6 Hz), 2.71 (m, 2H), 2.79 (td, 4H, *J* = 7.3, 3.0 Hz), 3.33 (q, 2H, *J* = 6.6 Hz), 3.76 (t, 2H, *J* = 4.8 Hz), 4.40 (t, 2H, *J* = 4.2 Hz), 7.14–7.19 (m, 2H), 7.23–7.33 (m, 8H), 7.82 (s, 2H) ppm. ^13^C-NMR (100 MHz, CDCl_3_): δ 21.2, 21.6, 25.5, 27.3, 29.4, 33.9, 40.5, 48.1, 56.2, 59.7, 63.1, 121.1, 127.5, 128.7, 145.0, 146.4, 150.0 ppm. IR (KBr): 548, 703, 761, 955, 1046, 1132, 1211, 1347, 1542, 1492, 1548, 1683, 2820, 2865, 2936, 3144 cm^−1^. HRMS (ESI-TOF): *m/z* [M + H]^+^ calcd for [C_37_H_49_N_8_]^+^ 605.4080; found 605.4084. Elementary analysis: calcd C 73.47, H 8.00, N 18.53; found C 73.12, H 8.27, N 18.26.

**1,3-Bis-(1-((1*S*,4*R*,5*R*)-2-((*S*)-1-phenylethyl)-2-azabicyclo[3.2.1]octan-4-yl)-1H-1,2,3-triazol-4-yl) propane (11)**


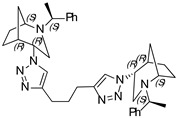


Off-white crystals. Yield 234 mg (73%). ${\left[\alpha \right]}_{D}^{25}$ = +46.7 (c 0.46, CH_2_Cl_2_). ^1^H-NMR (400 MHz, CDCl_3_): δ 1.09 (m, 2H), 1.32 (d, 6H, *J* = 6.6 Hz), 1.50–1.60 (m, 4H), 1.75–1.90 (m, 4H), 2.18 (qi, 2H, *J* = 7.6 Hz), 2.67 (dd, 2H, *J* = 9.7, 4.3 Hz), 2.81 (dd, 4H, *J* = 13.1, 4.6 Hz), 2.91 (t, 4H, *J* = 7.7 Hz), 3.16 (t, 2H, *J* = 4.8 Hz), 3.26 (d, 2H, *J* = 13.0 Hz), 3.47 (q, 2H, *J* = 6.6 Hz), 4.60 (t, 2H, *J* = 4.0 Hz), 7.22–7.25 (m, 2H), 7.28–7.35 (m, 8H), 8.12 (s, 2H) ppm. ^13^C-NMR (100 MHz, CDCl_3_): δ 21.7, 21.9, 25.6, 27.2, 29.8, 33.6, 40.4, 46.4, 57.5, 59.8, 62.2, 121.1, 127.1, 127.4, 128.7, 145.3, 146.6 ppm. IR (KBr): 550, 701, 761, 953, 1047, 1136, 1213, 1343, 1452, 1492, 1546, 2095, 2820, 2866, 2935, 3140 cm^−1^. HRMS (ESI-TOF): *m/z* [M + H]^+^ calcd for (C_37_H_49_N_8_)^+^ 605.4080; found 605.4088. Elementary analysis: calcd C 73.47, H 8.00, N 18.53; found C 73.56, H 8.30, N 18.14.

**(1*S*,1′*S*,4*S*,4′*S*,5*R*,5′*R*)-4,4′-(4,4′-(oxybis(methylene))bis(1H-1,2,3-triazole-4,1-diyl))bis(2-((*S*)-1-phenylethyl)-2-azabicyclo[3.2.1]octane) (12)**


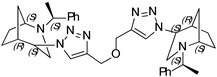


Yellow crystals. Yield 244 mg (83%). ${\left[\alpha \right]}_{D}^{25}$ = +118.9 (c 0.61, CH_2_Cl_2_). ^1^H-NMR (400 MHz, CDCl_3_): δ 1.27–1.32 (m, 2H), 1.38 (d, 6H, *J* = 6.5 Hz), 1.42–1.58 (m, 4H), 1.65 (d, 2H, *J* = 11.9 Hz), 1.80–1.88 (m, 4H), 2.55–2.60 (m, 2H), 2.69 (m, 4H), 3.34 (dd, 2H, *J* = 12.5, 6.1 Hz), 3.75 (s, 2H), 4.43 (s, 2H), 4.70 (d, 4H, *J* = 4.4 Hz), 7.15–7.19 (m, 2H), 7.24–7.33 (m, 8H), 8.07 (s, 2H) ppm. ^13^C-NMR (100 MHz, CDCl_3_): δ 21.1, 21.7, 27.3, 33.9, 40.4, 47.9, 56.3, 59.8, 63.1, 63.7, 123.1, 123.9, 127.4, 128.7, 136.1, 149.9 ppm. IR (KBr): 548, 703, 778, 956, 1046, 1093, 1132, 1225, 1346, 1452, 1492, 1599, 1684, 2820, 2866, 2965, 3145 cm^−1^. HRMS (ESI-TOF): *m/z* [M + H]^+^ calcd for (C_36_H_47_N_8_O)^+^ 607.3873; found 607.3885. Elementary analysis: calcd C 71.26, H 7.64, N 18.47; found C 71.52, H 7.76, N 18.21.

**(1*S*,1′*S*,4*R*,4′*R*,5*R*,5′*R*)-4,4′-(4,4′-(oxybis(methylene))bis(1H-1,2,3-triazole-4,1-diyl))bis(2-((*S*)-1-phenylethyl)-2-azabicyclo[3.2.1]octane) (13)**


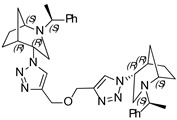


Off-white crystals. Yield 235 mg (76%). ${\left[\alpha \right]}_{D}^{25}$ = +23.5 (c 0.37, CH_2_Cl_2_). ^1^H-NMR (400 MHz, CDCl_3_): δ 1.07 (m, 2H), 1.30 (d, 6H, *J* = 6.6 Hz), 1.54 (d, 4H, *J* = 13.0 Hz), 1.74–1.89 (m, 6H), 2.67 (dd, 2H, *J* = 9.5, 4.3 Hz), 2.80 (dd, 2H, *J* = 13.1, 4.6 Hz), 3.15 (t, 2H, *J* = 4.7 Hz), 3.27 (d, 2H, *J* = 13.0 Hz), 3.44 (q, 2H, *J* = 6.6 Hz), 4.62 (t, 2H, *J* = 4.2 Hz), 4.83 (s, 4H), 7.21–7.29 (m, 2H), 7.32 (d, 8H, *J* = 4.4 Hz), 8.39 (s, 2H) ppm. ^13^C-NMR (100 MHz, CDCl_3_): δ 21.7, 21.8, 27.2, 33.5, 40.4, 46.5, 57.4, 59.9, 62.2, 64.7, 123.3, 127.4, 128.7, 143.5, 145.2, 150.0 ppm. IR (KBr): 551, 702, 760, 953, 1046, 1077, 1098, 1118, 1226, 1343, 1452, 1492, 1683, 2095, 2820, 2867, 2967, 3140 cm^−1^. HRMS (ESI-TOF): *m/z* [M + H]^+^ calcd for (C_36_H_47_N_8_O)^+^ 607.3873; found 607.3866. Elementary analysis: calcd C 71.26, H 7.64, N 18.47; found C 71.45, H 7.84, N 18.32.

**1,4-Bis-(1-((1*S*,4*S*,5*R*)-2-((*S*)-1-phenylethyl)-2-azabicyclo[3.2.1]octan-4-yl)-1H-1,2,3-triazol-4-yl) benzene (14)**


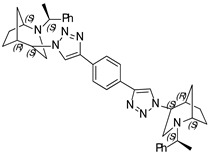


Brownish crystals. Yield 301 mg (94%). ${\left[\alpha \right]}_{D}^{25}$ = +321.3 (c 0.39, CH_2_Cl_2_). ^1^H-NMR (400 MHz, CDCl_3_): δ 1.30–1.36 (m, 2H), 1.42 (d, 6H, *J* = 6.5 Hz), 1.46–1.63 (m, 4H), 1.67 (d, 2H, *J* = 12.4 Hz), 1.84–1.94 (m, 4H), 2.64 (dd, 2H, *J* = 13.7, 4.4 Hz), 2.76 (d, 2H, *J* = 13.8 Hz), 2.81 (d, 2H, *J* = 4.1 Hz), 3.37 (q, 2H, *J* = 6.4 Hz), 3.82 (t, 2H, *J* = 4.3 Hz), 4.46 (s, 2H), 7.26–7.29 (m, 2H), 7.35–7.43 (m, 8H), 7.92 (s, 4H), 8.26 (s, 2H) ppm. ^13^C-NMR (100 MHz, CDCl_3_): δ 21.2, 21.7, 27.3, 33.8, 40.4, 48.2, 56.0, 59.7, 63.0, 120.2, 126.0, 127.4, 127.6, 128.7, 130.7, 145.2, 146.2 ppm. IR (KBr): 548, 702, 761, 956, 1057, 1132, 1225, 1348, 1452, 1491, 1682, 2096, 2820, 2866, 2966, 3141 cm^−1^. HRMS (ESI-TOF): *m/z* [M + H]^+^ calcd for (C_40_H_47_N_8_)^+^ 639.3924; found 639.3921. Elementary analysis: calcd C 75.20, H 7.26, N 17.54; found C 74.87, H 7.43, N 17.22.

**1,4-Bis-(1-((1*S*,4*R*,5*R*)-2-((*S*)-1-phenylethyl)-2-azabicyclo[3.2.1]octan-4-yl)-1H-1,2,3-triazol-4-yl) benzene (15)**


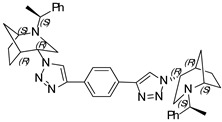


Off-white crystals. Yield 239 mg (68%). ${\left[\alpha \right]}_{D}^{25}$ = +106.2 (c 0.37, CH_2_Cl_2_). ^1^H-NMR (400 MHz, CDCl_3_): δ 1.12–1.17 (m, 1H), 1.38 (d, 3H, *J* = 6.7 Hz), 1.57–1.64 (m, 2H), 1.79–1.94 (m, 3H), 2.75 (dd, 1H, *J* = 9.5, 4.3 Hz), 2.90 (dd, 1H, *J* = 13.2, 4.6 Hz), 3.24 (t, 1H, *J* = 4.7 Hz), 3.31 (d, 1H, *J* = 13.1 Hz), 3.56 (q, 1H, *J* = 6.6 Hz), 4.66 (t, 1H, *J* = 4.0 Hz), 7.26–7.31 (m, 1H), 7.35–7.39 (m, 4H), 7.98 (s, 2H), 8.56 (s, 1H) ppm. ^13^C-NMR (100 MHz, CDCl_3_): δ 21.1, 22.3, 27.2, 33.6, 40.4, 46.1, 58.0, 59.9, 62.2, 120.0, 126.2, 127.3, 127.5, 128.7, 130.8, 145.1, 146.5, 149.9 ppm. IR (KBr): 542, 701, 761, 1054, 1225, 1345, 1451, 1492, 1722, 2821, 2867, 2966, 3136 cm^−1^. HRMS (ESI-TOF): *m/z* [M + H]^+^ calcd for (C_40_H_47_N_8_)^+^ 639.3924; found 639.3937. Elementary analysis: calcd C 75.20, H 7.26, N 17.54; found C 75.52, H 7.13, N 17.25.

**1,3-Bis-(1-((1*S*,4*S*,5*R*)-2-((*S*)-1-phenylethyl)-2-azabicyclo[3.2.1]octan-4-yl)-1H-1,2,3-triazol-4-yl) benzene (16)**


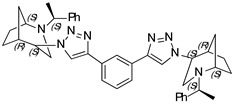


Yellow crystals. Yield 307 mg (96%). ${\left[\alpha \right]}_{D}^{25}$ = +307.9 (c 0.93, CH_2_Cl_2_). ^1^H-NMR (400 MHz, CDCl_3_): δ 1.32–1.38 (m, 2H), 1.40 (d, 6H, *J* = 7.0 Hz), 1.47–1.63 (m, 4H), 1.69 (d, 2H, *J* = 12.8 Hz), 1.88 (m, 4H), 2.62 (dd, 2H, *J* = 4.9, 13.8 Hz), 2.76 (d, 2H, 13.9 Hz), 3.35 (q, 2H, *J* = 13.9 Hz), 3.81 (t, 2H, *J* = 5.4 Hz), 4.47 (t, 2H, *J* = 5,4 Hz), 7.21–7.30 (m, 2H), 7.38–7.46 (m, 10H), 7.53 (t, 2H, *J* = 7.7 Hz), 7.82 (dd, 2H, *J* = 1.9, 7.9 Hz), 8.34 (s, 2H). ^13^C-NMR (100 MHz, CDCl_3_): δ 21.3, 21.7, 27.4, 33.9, 40.4, 48.1, 56.1, 59.8, 63.1, 120.5, 122.7, 124.9, 127.4, 127.7, 128.9, 129.4, 131.9, 145.1, 146.4 ppm. IR (KBr): 548, 703, 778, 1057, 1132, 1226, 1347, 1452, 1492, 1616, 1683, 2820, 2866, 2953, 3140 cm^−1^. HRMS (ESI-TOF): *m/z* [M + H]^+^ calcd for (C_40_H_47_N_8_)^+^ 639.3924; found 639.3920. Elementary analysis: calcd C 75.20, H 7.26, N 17.54; found C 74.94, H 7.51, N 17.31.

**1,3-Bis-(1-((1*S*,4*R*,5*R*)-2-((*S*)-1-phenylethyl)-2-azabicyclo[3.2.1]octan-4-yl)-1H-1,2,3-triazol-4-yl) benzene (17)**


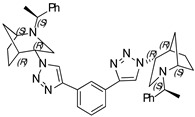


White crystals. Yield 239 mg (71%). ${\left[\alpha \right]}_{D}^{25}$ = +72.1 (c 0.43, CH_2_Cl_2_). ^1^H-NMR (400 MHz, CDCl_3_): δ 1.12–1.17 (m, 2H), 1.35 (t, 6H, *J* = 6.7 Hz), 1.37–1.42 (m, 2H), 1.53–1,70 (m, 6H), 1.78–1.93 (m, 4H), 2.76 (dd, 2H, *J* = 9.5, 4.3 Hz), 2.89 (dd, 2H, *J* = 13.1, 4.5 Hz), 3.23 (t, 2H, *J* = 4.8 Hz), 3.31 (d, 2H, *J* = 13.1 Hz), 3.54 (q, 2H, *J* = 6.6 Hz), 4.67 (t, 2H, 3.9 Hz), 7.23–7.28 (m, 2H), 7.33–7.93 (m, 8H), 7.87 (dd, 2H, *J* = 7.7, 1.7 Hz), 8.59 (s, 2H) ppm. ^13^C-NMR (100 MHz, CDCl_3_): δ 21.1, 22.3, 27.2, 33.6, 40.4, 46.2, 57.8, 60.0, 62.3, 120.2, 123.2, 125.5, 127.2, 127.5, 128.7, 129.4, 131.9, 144.9, 146.6 ppm. IR (KBr): 550, 700, 769, 953, 1054, 1137, 1226, 1344, 1452, 1492, 1616, 2095, 2822, 2867, 2967, 3136 cm^−1^. HRMS (ESI-TOF): *m/z* [M + H]^+^ calcd for (C_40_H_47_N_8_)^+^ 639.3924; found 639.3939. Elementary analysis: calcd C 75.20, H 7.26, N 17.54; found C 75.02, H 7.29, N 17.43.

***N*,*N*,*N*-tri((1-((1*S*,4*S*,5*R*)-2-((*S*)-1-phenylethyl)-2-azabicyclo[3.2.1]octan-4-yl)-1H-1,2,3-triazol-4-yl)methyl) amine (18)**


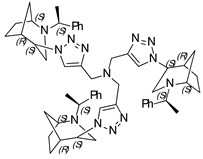


Yellow Crystals. Yield 222 mg (76%). ${\left[\alpha \right]}_{D}^{25}$ = +233.3 (c 0.06, CH_2_Cl_2_). ^1^H-NMR (400 MHz, CDCl_3_): δ 1.29–1.34 (m, 3H), 1.39 (d, 9H, *J* = 6.6 Hz), 1.34–1.73 (m, 9H), 1.79 (d, 3H, *J* = 12.9 Hz), 1.82–1.89 (m, 6H), 2.56 (dd, 3H, *J* = 4.8, 13.6 Hz), 2.68–2.75 (m, 6H), 3.34 (q, 3H, *J* = 6.4 Hz), 3.78 (t, 3H, *J* = 4.7 Hz), 3.82 (s, 3H), 4.47 (t, 3H, *J* = 4.0 Hz), 7.06–7.19 (m, 6H), 7.25–7-37 (m, 9H), 8.29 (s, 3H) ppm. ^13^C-NMR (100 MHz, CDCl_3_): δ 21.3, 21.6, 27.4, 33.9, 40.5, 47.1, 48.0, 56.3, 59.9, 63.1, 123.9, 127.3, 127.6, 128.7, 136.1, 144.9, 150.0 ppm. IR (KBr): 550, 703, 759, 956, 1045, 1132, 1211, 1335, 1452, 1492, 1650, 2821, 2867, 2953, 3143 cm^−1^. HRMS (ESI-TOF): *m/z* [M + H]^+^ calcd for (C_54_H_70_N_13_)^+^ 900.5877; found 900.5876. Elementary analysis: calcd C 72.05, H 7.73, N 20.23; found C 72.32, H 7.46, N 20.11.

***N*,*N*,*N*-tris((1-((1*S*,4*R*,5*R*)-2-((*S*)-1-phenylethyl)-2-azabicyclo[3.2.1]octan-4-yl)-1H-1,2,3-triazol-4-yl)methyl) amine (19)**


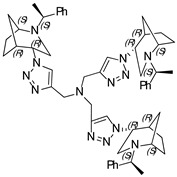


Yellow crystals. Yield 213 mg (70%). ${\left[\alpha \right]}_{D}^{25}$ = +35.6 (c 0.43, CH_2_Cl_2_). ^1^H-NMR (600 MHz, CDCl_3_): δ 1.08 (m, 3H), 1.24 (s, 3H), 1.34 (d, 9H, *J* = 6.6 Hz), 1.44–1.63 (m, 9H), 1.75–1.86 (m, 6H), 2.71 (m, 3H), 2.81 (dd, 3H, *J* = 12.9, 4.4 Hz), 3.15 (t, 3H, *J* = 4.5 Hz), 3.36 (d, 3H, *J* = 13.0 Hz), 3.43 (d, 3H, *J* = 6.5 Hz), 3.95 (d, 3H, *J* = 9.4 Hz), 4.63 (t, 3H, *J* = 3.8 Hz), 7.18–7.21 (m, 3H), 7.24–7.33 (m, 12H), 8.60 (d, 3H, *J* = 4.2 Hz) ppm. ^13^C-NMR (100 MHz, CDCl_3_): δ 21.7, 21.9, 27.2, 33.6, 40.5, 46.6, 57.3, 59.9, 61.2, 123.8, 127.0, 127.7, 128.6, 136.0, 145.5, 149.9 ppm. IR (KBr): 551, 702, 801, 1045, 1098, 1262, 1335, 1452, 1492, 1644, 1721, 2821, 2866, 2965, 3140 cm^−1^. HRMS (ESI-TOF): *m/z* [M + H]^+^ calcd for (C_54_H_70_N_13_)^+^ 900.5877; found 900.5879. Elementary analysis: calcd C 72.05, H 7.73, N 20.23; found C 71.77, H 8.05, N 19.91.

Compound **20**, C_31_H_39_N_5_. Elementary analysis: calcd C 77.30, H 8.16, N 14.54; found C 77.06, H 8.38, N 14.24.

Compound **21**, C_26_H_37_N_5_O_2_. Elementary analysis: calcd C 69.15, H 8.26, N 15.51; found C 68.93, H 8.48, N 15.25.

### 2.2. Biological Activity Analysis

#### 2.2.1. Cell Culture

Three human cancer cell lines were used to evaluate the antiproliferative activity of the obtained compounds: melanoma cell line Hs294T, pancreatic tumor cell line MIA PaCa-2, and lung tumor cell line NCI-H1581. The normal mouse fibroblasts cell line (BALB/3T3 clone A31) was selected as reference. All these lines were purchased from the ATCC (American Type Culture Collection, Rockville, MD, USA). NCI-H1581 cells were grown in RPMI 1640 with GlutaMax medium (Gibco, Thermo Fisher Scientific, Leicestershire, UK) with 5% fetal bovine serum (FBS; HyClone, GE Healthcare, Logan, UT, USA). Hs294T and BALB/3T3 cells were grown in Dulbecco’s Modified Eagle Medium (DMEM; Gibco, Thermo Fisher Scientific, Leicestershire, UK) supplemented with 2 mM L-glutamine and 10% FBS (HyClone, GE Healthcare, Logan, UT, USA). MIA PaCa-2 cells were grown in DMEM (Gibco, Thermo Fisher Scientific, Leicestershire, UK) supplemented with 2 mM L-glutamine, 2.5% Horse Serum (Gibco, Thermo Fisher Scientific, Leicestershire, UK), and 10% FBS (HyClone, GE Healthcare, Logan, UT, USA). All culture media were supplemented with antibiotics: 100 U/mL penicillin (Polfa Tarchomin SA, Warsaw, Poland) and 100 µg/mL streptomycin (Sigma-Aldrich Chemie GmbH, Steinheim, Germany); cells were grown at 37 °C in a humid atmosphere with 5% CO_2_.

#### 2.2.2. Growth Inhibition Assay (SRB Assay)

Twenty-four hours prior to the addition of the tested compounds, 2000 cells per well of Balb/3T3, MIA PaCa-2, and Hs294T cell lines and 2500 cells of NCI-H1581 cell line were seeded in 384-well plates (Greiner Bio-One, Kremsmünster, Austria) in an appropriate medium. The studied compounds were added to the cells at 8 different concentrations in a series of dilutions from 100 to 0.00001 µg/mL for 72 h. Cisplatin was applied as a reference compound (Teva Pharmaceuticals, Warsaw, Poland). DMSO (Sigma-Aldrich Chemie GmbH, Steinheim, Germany) was used as solvent control. The DMSO concentrations corresponded to the solvent concentrations in the tested compound solutions. Then, 30 µL per well of ice-cold 50% TCA (trichloroacetic acid, Avantor, Gliwice, Poland) was added to the cells. After one hour of incubation at room temperature (RT) cells were washed five times with water and stained with 0.4% SRB (sulforhodamine B, Sigma-Aldrich Chemie GmbH, Steinheim, Germany). Twenty-five microliters per well of 0.4% solution of SRB in 1% acetic acid (Avantor, Gliwice, Poland) was applied. After 0.5 h of incubation at RT, the unbound dye was washed out with 1% acetic acid. The bound dye in each well was dissolved in 70 µl of 10 mM TRIS (Tris base, Sigma-Aldrich, Chemie GmbH, Steinheim, Germany). A microplate reader (BioTek Synergy H4, Swindon, UK) with Gen5 software was used to read the absorbance at 540 nm [21]. The results are presented as mean IC_50_ values (concentration of the compound that inhibits cell proliferation by 50%) ± standard deviation. Half-maximal inhibitory concentration (IC_50_) values were calculated using the Prolab-3 system based on Cheburator 0.4 [22]. Each experiment was performed in triplicate, and three independent repetitions were made for each experiment. Raw data and IC_50_ values were placed in the supplementary materials.

## 3. Results

### 3.1. Synthesized Compounds

**Scheme 1:**
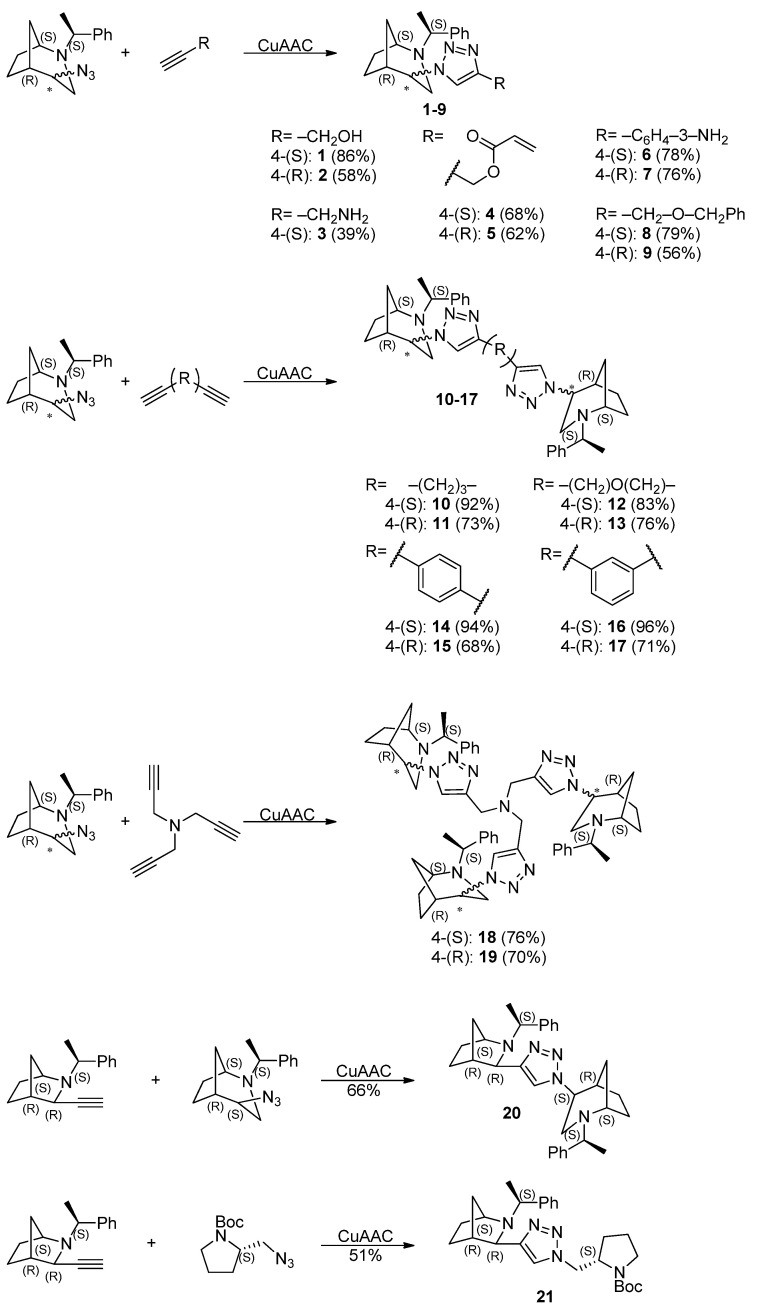
Synthesis of mono-, di-, and trimeric triazoles based on 2-azabicycloalkanes.

A library of 21 compounds was obtained (Scheme 1) using copper(I)-catalyzed azide-alkyne cycloaddition (CuAAC) procedure yielding pure 1,2,3-triazoles incorporating 2-azabicycloalkane moieties. Attention was paid to cover various aspects: number of triazole rings, linker, configuration, and type of the 2-azabicycloalkane moiety. The bicyclic starting materials were synthesized as earlier described by us [19,20]. Assemblies containing either one, two, or three triazoles and 2-azabicycloalkane subunits were obtained using different substrates and linkers to investigate their influence on the antiproliferative activity. Properties, such as hydrophilicity and rigidity, were especially considered when choosing the linker and substituents. It has to be pointed out that, for the dimeric and trimeric derivatives, no complex mixtures of partially clicked products were observed, but only the desired product was received after purification process. Furthermore, for each compound, the epimer differing in the configuration of 4C was prepared (except for the 4-(*R*)-epimer of **3**). At last, an approach was made to include not only the 7-membered 2-azabicyclo-[3.2.1]octane moiety (**1–20**) but also a 6-membered 2-azabicyclo[2.2.1]heptane skeleton (**20**,**21**). The structure and absolute configuration of the two latter compounds have been recently studied by our group [20].

### 3.2. Antiproliferative Activity

All compounds, **1–21**, were evaluated for their antiproliferative activity towards three human cancer cell lines: melanoma Hs294T, pancreatic MIA PaCa-2 and lung NCI-H1581 cancer and evaluated against normal murine fibroblasts cell line BALB/3T3 clone A31 to check the selectivity of the tested compounds (Table 1). Cisplatin served as reference compound. A valuable insight into the structure-activity relationship of the triazoles was achieved. All IC_50_ values can be found in Table 1 (raw data are placed in the supplementary materials); in the following discussion, they are provided without the standard deviation for the benefit of readability.

Overall, good results were found for the cell line NCI-H1581, with IC_50_ values as low as 2.4 μM for compound **18**, compared to 7.3 μM for cisplatin. In total 8 of the 21 triazole compounds exhibited a better activity towards this lung cancer cell line than cisplatin, and some of them were vastly less toxic towards BALB/3T3 (the selectivity indices are presented in Table 1). Whilst no notable results were achieved for pancreatic cancer cell line MIA PaCa‑2, better antiproliferative activities were noted towards melanoma cell line Hs294T. Compounds **4**, **10**, **11**, and **13** exhibited IC_50_ values in the range of 9.5 to 13.7 μM compared to cisplatin with 1.3 μM; the most active compound **4**, however, also exhibited the highest toxicity towards normal murine fibroblast cells.

### 3.3. Structure—Activity Analysis

As mentioned before, three aspects were covered by the synthesized triazoles: number of triazole units, configuration and type of 2-azabicycloalkane (7-membered or 6-membered ring). Structure-activity analysis of all three characteristics resulted in the following conclusions. Taking a look on the most active compounds (IC_50_ < 15 μM against NCI-H1581 and/or Hs294T), a first correlation can be found. Especially, the antiproliferative activities against the lung cancer cell line NCI-H1581 reveal a pattern towards an increasing activity accompanied by introduction of additional triazole and azabicycloalkane moieties. This indicates that the activity towards this cell line can originate from both structural motifs. Furthermore, it shows a clear trend from monomeric, over dimeric to trimeric compounds accompanied by increasing activities, particularly for NCI-H1581 line, due to more active groups in the molecule. However, since there are only two trimeric compounds studied, further investigation is necessary to provide more evidence.

The linker between the active moieties plays a prime role. Whilst more rigid structures as in **14–17** result in lower activities, the more flexible assemblies **10–13** exhibit both good activities towards lung cancer cell line NCI-H1581, as well as melanoma cell line Hs294T and rather low toxicities for normal murine fibroblast cells BALB/3T3. The use of either the alkyl-linker (**10**, **11**) or ether-linker (**12**, **13**) results in comparable activities and low toxicity for both epimers; compounds **11** (26.7 μM, SI = 6) and **13** (46.1 μM, SI = 9; for cisplatin SI = 0.3) exhibit particularly good antiproliferative activities against NCI-H1581 and Hs294T.

Various criteria of selectivity can be found in the literature. Certainly, a safety margin is required; thus, just observation that a given compound exhibits higher toxicity for cancer cells than for regular cells is not sufficient. Some approaches describe SI values above 2 or 3 as promising [23,24]; however, a general unification is hard, also considering the use of different cancer cell lines and methods for evaluation. Taking this into account, we can say that, in our study, very good results were achieved with 12 compounds with SI > 3, whereas 10 have SI above 6, and three have as high as 9 or 9.5.

Comparing the epimers 4-(*S*) and 4-(*R*) the activity of the 2-azabicycloalkane moiety can be proved. A generally higher activity of the 4-(*R*)-epimers can be seen (**1** vs. **2**, **6** vs. **7**, **8** vs. **9**, **12** vs. **13**, **14** vs. **15,** and **18** vs. **19**), revealing also a clear influence of the 2-azabicycloalkane moiety on the antiproliferative action.

A comparison of the activities of **20** and **21** show that the activity originates from the 7-membered 2-azabicycloalkane moiety (6.3 μM), rather than the 6-membered one (77.6 μM). The difference in activity against NCI‑H1581 also supports the previous assumption of the origin of the antiproliferative activity of the synthesized triazoles laying within the 7-membered 2-azabicylcoalkane moiety. This fact is supported by a generally good antiproliferative activity of the monomeric triazoles (with the exception of hydroxymethyl derivatives **1** and **2**).

## 4. Conclusions

In summary, a library of 21 triazoles bearing 2-azabicycloalkanes was obtained and evaluated for their antiproliferative activities. Among tested compounds, **11** and **13** were revealed to efficiently inhibit proliferation of both lung cancer and melanoma cell lines, which is accompanied by good selectivity (6–9 times higher activity against lung cancer cells vs. mouse fibroblasts). Compounds **18** and **19**, highly active only against lung cancer cells, also have high selectivity against neoplastic cells (SI above 8). The study can be regarded as a promising starting point for further modifications and investigations.

## Figures and Tables

**Table 1:** Antiproliferative activity of triazoles **1–21**.

Compound	IC50 [µM]				SI
	Hs294T	MIA PaCa-2	NCI-H1581	BALB3T3	
Cisplatin	1.30 ± 0.50	12.63 ± 3.13	7.27 ± 2.83	1.83 ± 0.70	0.3
1	na *	na	na	na	-
2	na	na	72.34 ± 33.99	na	-
3	88.53 ± 9.41	99.90 ± 4.46	11.27 ± 1.25	94.44 ± 1.89	8
4	9.52 ± 2.51	87.73 ± 1.66	7.91 ± 1.20	1.75 ± 0.76	0.2
5	72.8 ± 9.71	86.31 ± 0.35	18.77 ± 4.45	28.54 ± 22.68	1.5
6	74.54 ± 14.94	84.42 ± 1.04	30.76 ± 3.51	77.08 ± 1.10	2.5
7	65.87 ± 8.30	76.95 ± 1.98	7.39 ± 1.15	52.69 ± 6.16	7
8	73.81 ± 10.11	79.12 ± 0.5	15.48 ± 0.12	61.46 ± 3.28	4
9	70.88 ± 5.86	76.47 ± 4.37	8.52 ± 1.39	50.03 ± 3.25	6
10	10.35 ± 3.39	40.19 ± 1.60	4.20 ± 0.36	11.85 ± 5.03	3
11 **	9.71 ± 3.08	37.58 ± 5.51	4.40 ± 0.61	26.72 ± 4.76	6
12	32.42 ± 7.83	51.47 ± 1.60	6.00 ± 0.86	44.40 ± 7.50	7
13	13.65 ± 2.31	44.91 ± 3.69	4.88 ± 0.41	46.06 ± 11.29	9
14 **	77.26 ± 9.50	na	20.91 ± 15.20	na	-
15 **	58.82 ± 4.07	111.47 ± 9.55	6.87 ± 1.46	59.5 ± 11.71	9
16 **	na	na	53.93 ± 5.07	na	-
17	na	na	na	na	-
18	20.83 ± 5.02	25.67 ± 0.26	2.44 ± 0.07	23.08 ± 2.72	9.5
19	29.13 ± 1.36	31.84 ± 2.31	3.27 ± 0.42	27.65 ± 3.24	8.5
20	57.01 ± 13.64	63.01 ± 7.29	6.25 ± 1.37	39.40 ± 4.86	6
21 **	92.58 ± 21.97	90.28 ± 0.69	77.57 ± 8.68	na	-

* not active (IC_50_ > 100 µM), ** poor or no solvability in dimethyl sulfoxide (DMSO). The IC_50_ value defined as the concentration of a compound which inhibited cell proliferation in 50%. The SI (Selectivity Index) was calculated for each compound using the formula: SI = IC_50_ for normal cell line BALB/3T3/ IC_50_ for NCI-H1581 human lung cancer cell line. The beneficial SI > 1.0 indicates a compound with efficacy against tumor cells greater than the toxicity against normal cells.

## Data Availability

Not applicable.

## Supplementary Materials

The following are available online at https://www.mdpi.com/article/10.3390/ma14082039/s1, Experimental data and ^1^H and ^13^C-NMR spectra of novel compounds **1–****19** and their IC_50_’s as raw [µg/mL] and processed data [µM].

## References

[1] World Health Organisation Cancer. https://www.who.int/news-room/fact-sheets/detail/cancer.

[2] Yet L. (2018). Privileged Structures in Drug Discovery: Medicinal Chemistry and Synthesis.

[3] Bräse S. (2015). Privileged Scaffolds in Medicinal Chemistry: Design, Synthesis, Evaluation.

[4] Thirumurugan P., Matosiuk D., Jozwiak K. (2013). Click chemistry for drug development and diverse chemical-biology applications. Chem. Rev..

[5] Lauria A., Delisi R., Mingoia F., Terenzi A., Martorana A., Barone G., Almerico A.M. (2014). 1,2,3-triazole in heterocyclic compounds, endowed with biological activity, through 1,3-dipolar cycloadditions. Eur. J. Org. Chem..

[6] Wojaczynska E., Wojaczynski J. (2018). Synthesis and Applications of 1,2,3-Triazoles. Adv. Org. Syntehsis.

[7] Bonnefond M.L., Florent R., Lenoir S., Lambert B., Abeilard E., Giffard F., Louis M.H., Elie N., Briand M., Vivien D. (2018). Inhibition of store-operated channels by carboxyamidotriazole sensitizes ovarian carcinoma cells to anti-BclxL strategies through Mcl-1 down-regulation. Oncotarget.

[8] Gomez L., Kovac J.R., Lamb D.J. (2015). CYP17A1 inhibitors in castration-resistant prostate cancer. Steroids.

[9] Pokhodylo N. (2013). Synthesis of 1,2,3-Triazole Derivatives and Evaluation of their Anticancer Activity. Sci. Pharm..

[10] Elamari H., Slimi R., Chabot G.G., Quentin L., Scherman D., Girard C. (2013). Synthesis and in vitro evaluation of potential anticancer activity of mono- and bis-1,2,3-triazole derivatives of bis-alkynes. Eur. J. Med. Chem..

[11] Aouad M., Soliman M., Alharbi M., Bardaweel S., Sahu P., Ali A., Messali M., Rezki N., Al-Soud Y. (2018). Design, Synthesis and Anticancer Screening of Novel Benzothiazole-Piperazine-1,2,3-Triazole Hybrids. Molecules.

[12] Xu Z., Zhao S.J., Liu Y. (2019). 1,2,3-Triazole-containing hybrids as potential anticancer agents: Current developments, action mechanisms and structure-activity relationships. Eur. J. Med. Chem..

[13] Valdomir G., Fernández M.D.L.A., Lagunes I., Padrón J.I., Martín V.S., Padrón J.M., Davyt D. (2018). Oxa/thiazole-tetrahydropyran triazole-linked hybrids with selective antiproliferative activity against human tumour cells. New J. Chem..

[14] Singh K., Gangrade A., Jana A., Mandal B.B., Das N. (2019). Design, Synthesis, Characterization, and Antiproliferative Activity of Organoplatinum Compounds Bearing a 1,2,3-Triazole Ring. ACS Omega.

[15] El Malah T., Abdel Mageid R.E., Awad H.M., Nour H.F. (2020). Copper(i)-catalysed azide-alkyne cycloaddition and antiproliferative activity of mono- A nd bis-1,2,3-triazole derivatives. New J. Chem..

[16] Kamińska K., Wojaczyńska E., Wietrzyk J., Turlej E., Błażejczyk A., Wieczorek R. (2016). Synthesis, structure and antiproliferative activity of chiral polyamines based on a 2-azanorbornane skeleton. Tetrahedron Asymmetry.

[17] Samadaei M., Pinter M., Senfter D., Madlener S., Rohr-Udilova N., Iwan D., Kamińska K., Wojaczyńska E., Wojaczyński J., Kochel A. (2020). Synthesis and Cytotoxic Activity of Chiral Sulfonamides Based on the 2-Azabicycloalkane Skeleton. Molecules.

[18] Boratyński P.J., Kowalczyk R. (2016). Click-Dimerized Cinchona Alkaloids. J. Org. Chem..

[19] Wojaczyńska E., Turowska-Tyrk I., Skarzewski J. (2012). Novel chiral bridged azepanes: Stereoselective ring expansion of 2-azanorbornan-3-yl methanols. Tetrahedron.

[20] Steppeler F., Górecki M., Wojaczyńska E. (2021). Synthesis of terminal alkynes based on (1S,3R,4R)- and (1S,3S,4R)-2-azabicyclo[2.2.1]heptane. Arkivoc.

[21] Skehan P., Storeng R., Scudiero D., Monks A., McMahon J., Vistica D., Warren J.T., Bokesch H., Kenney S., Boyd M.R. (1990). New Colorimetric Cytotoxicity Assay for Anticancer-Drug Screening. JNCI J. Natl. Cancer Inst..

[22] Nevozhay D. (2014). Cheburator Software for Automatically Calculating Drug Inhibitory Concentrations from In Vitro Screening Assays. PLoS ONE.

[23] Suffness M., Pezzuto J.M., Hostettmann K. (1990). Assays related to cancer drug discovery. Methods in Plant Biochemistry: Assays for Bioactivity.

[24] Bézivin C., Tomasi S., Lohezic-Le Devehat F., Boustie J. (2003). Cytotoxic activity of some lichen extracts on murine and human cancer cell lines. Phytomedicine.

