# Supplementary material for: Synthesis and Antiproliferative Activity of Triazoles Based on 2-Azabicycloalkanes
Source: Materials (Basel). 2021 Apr 18;14(8):2039. doi: 10.3390/ma14082039 (PMC8072719; doi:10.3390/ma14082039)
Supplement: Supplementary file 1 [file materials-14-02039-s001.zip › materials-1134252-supplementary.pdf]

materials

Supplementary Information

Synthesis and antiproliferative activity of triazoles based on 2-azabicycloalkanes

Franz Steppeler[1], Dagmara Kłopotowska[2], Joanna Wietrzyk[2] and Elżbieta Wojaczyńska [1,*]

[1] Faculty of Chemistry, Wrocław University of Science and Technology, Wybrzeże Wyspiańskiego 27, 50-370 Wrocław, Poland; franz.steppeler@pwr.edu.pl
[2] Hirszfeld Institute of Immunology and Experimental Therapy, Polish Academy of Sciences, ul. Rudolfa Weigla 12, 53-114 Wrocław, Poland; dagmara.klopotowska@hirszfeld.pl (D.K.); joanna.wietrzyk@hirszfeld.pl (J.W.)
* Correspondence: elzbieta.wojaczynska@pwr.edu.pl

Citation: Steppeler, F.; Kłopotowska , D.; Wietrzyk , J.; Wojaczyńska, E. Synthesis and antiproliferative activity of triazoles based on 2-azabicycloalkanes. *Materials* **2021**, *14*, 2039. https://doi.org/10.3390/ma14082039

Academic Editor: Nicola Margiotta

Received: 18 February 2021
Accepted: 15 April 2021
Published: 18 April 2021

Publisher's Note: MDPI stays neutral with regard to jurisdictional claims in published maps and institutional affiliations.

Copyright: © 2021 by the authors. Licensee MDPI, Basel, Switzerland. This article is an open access article distributed under the terms and conditions of the Creative Commons Attribution (CC BY) license (http://creativecommons.org/licenses/by/4.0/).

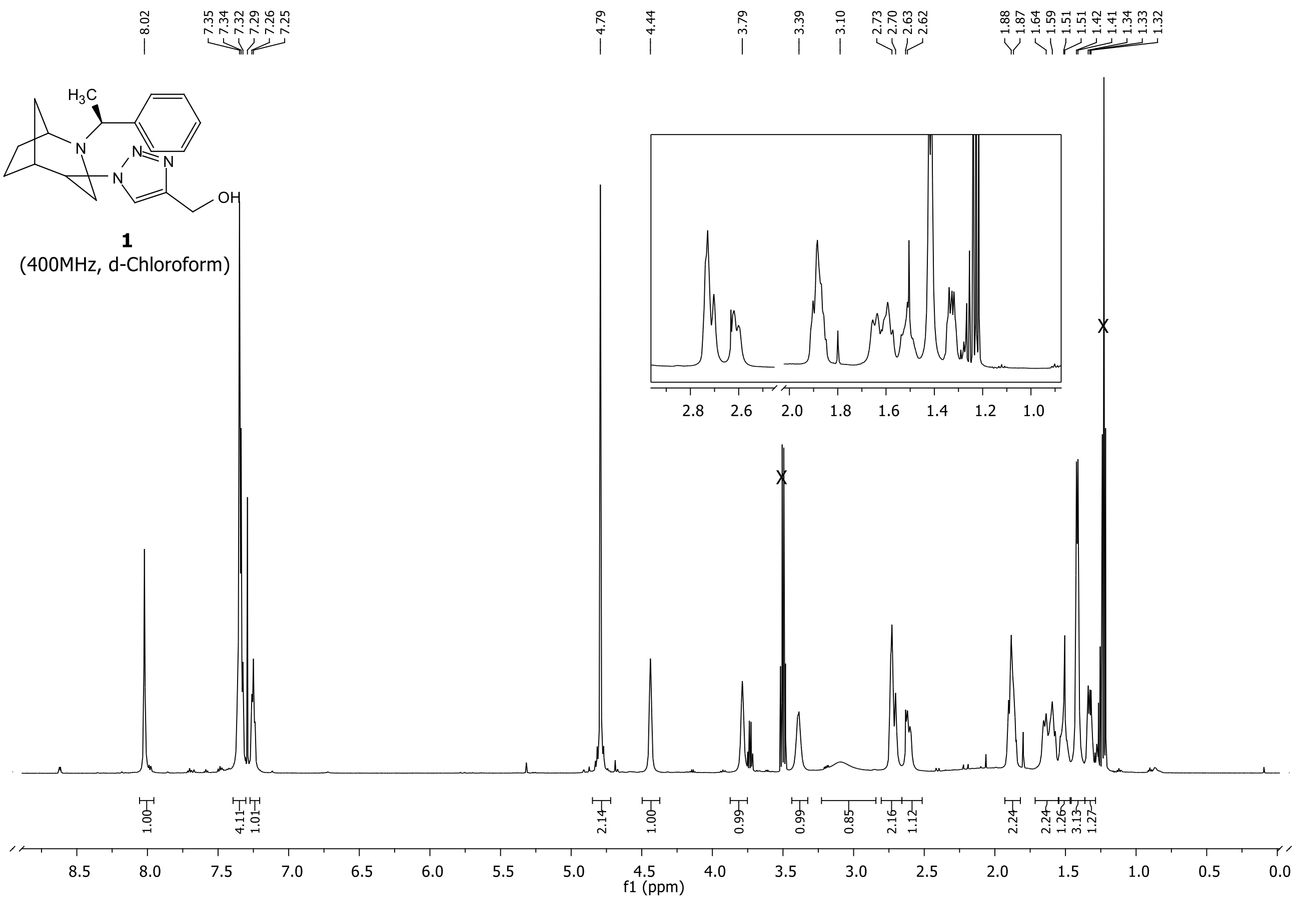
1
(400MHz, d-Chloroform)
f1 (ppm)

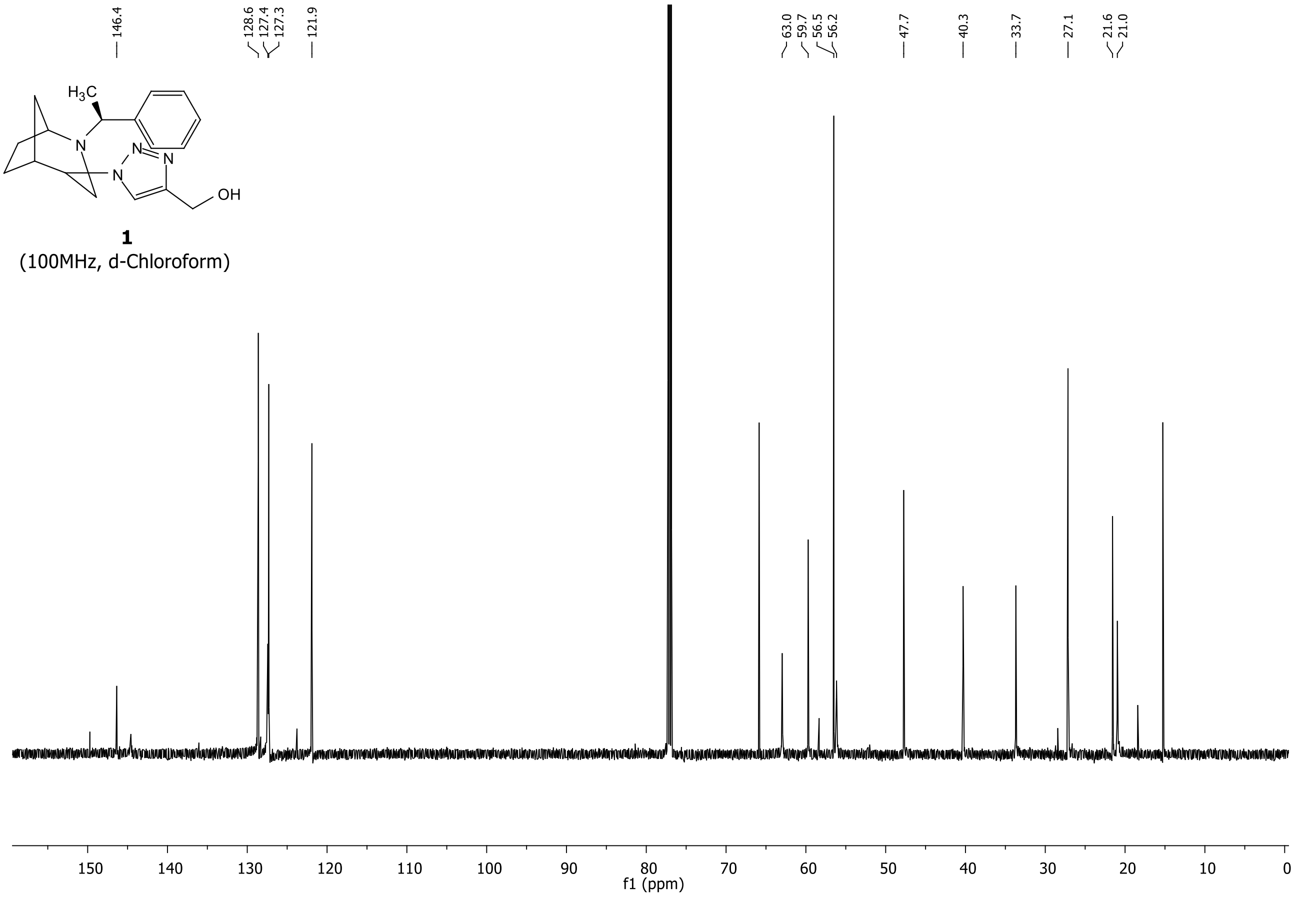
1
(100MHz, d-Chloroform)
f1 (ppm)

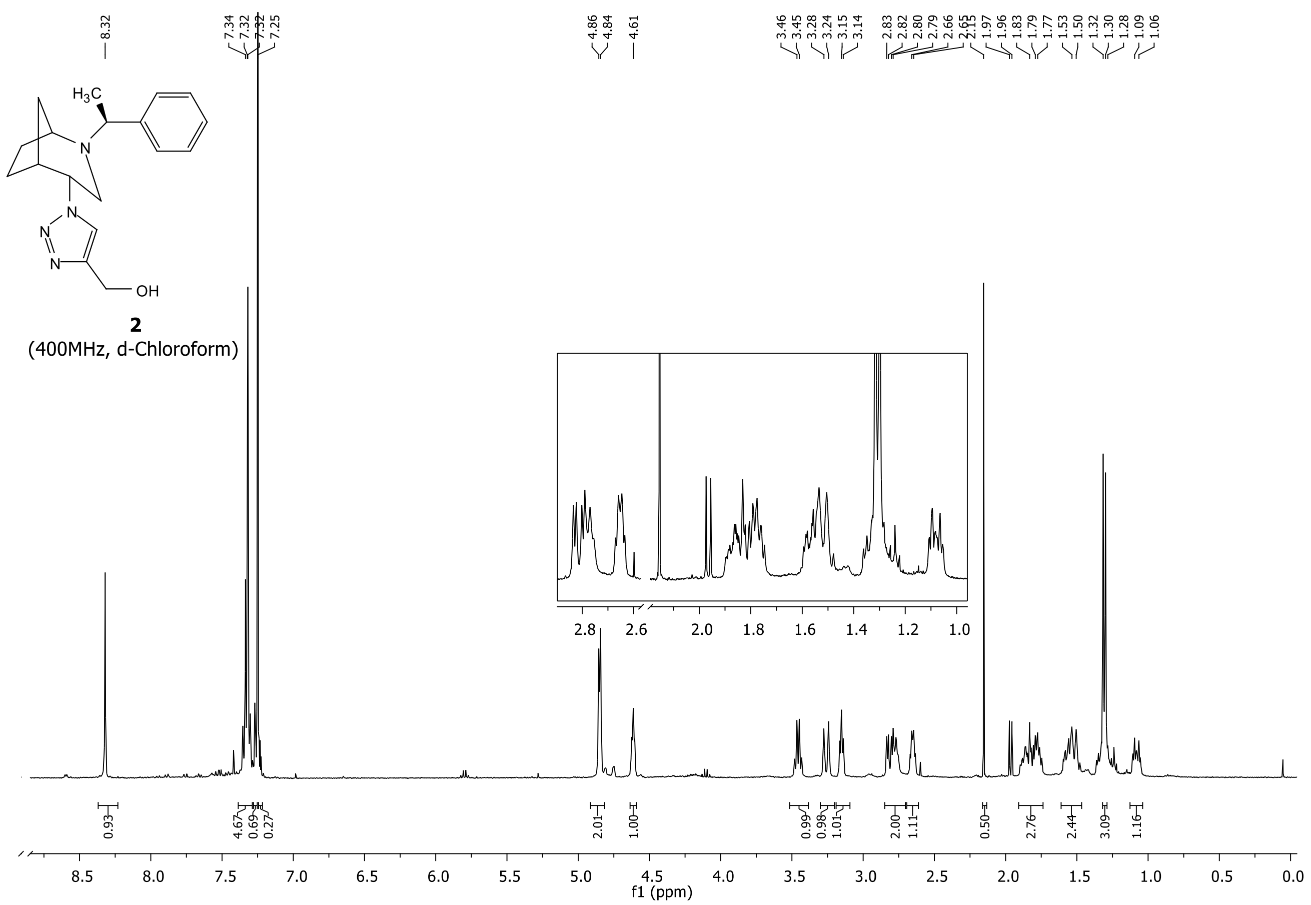

2
(400MHz, d-Chloroform)
f1 (ppm)

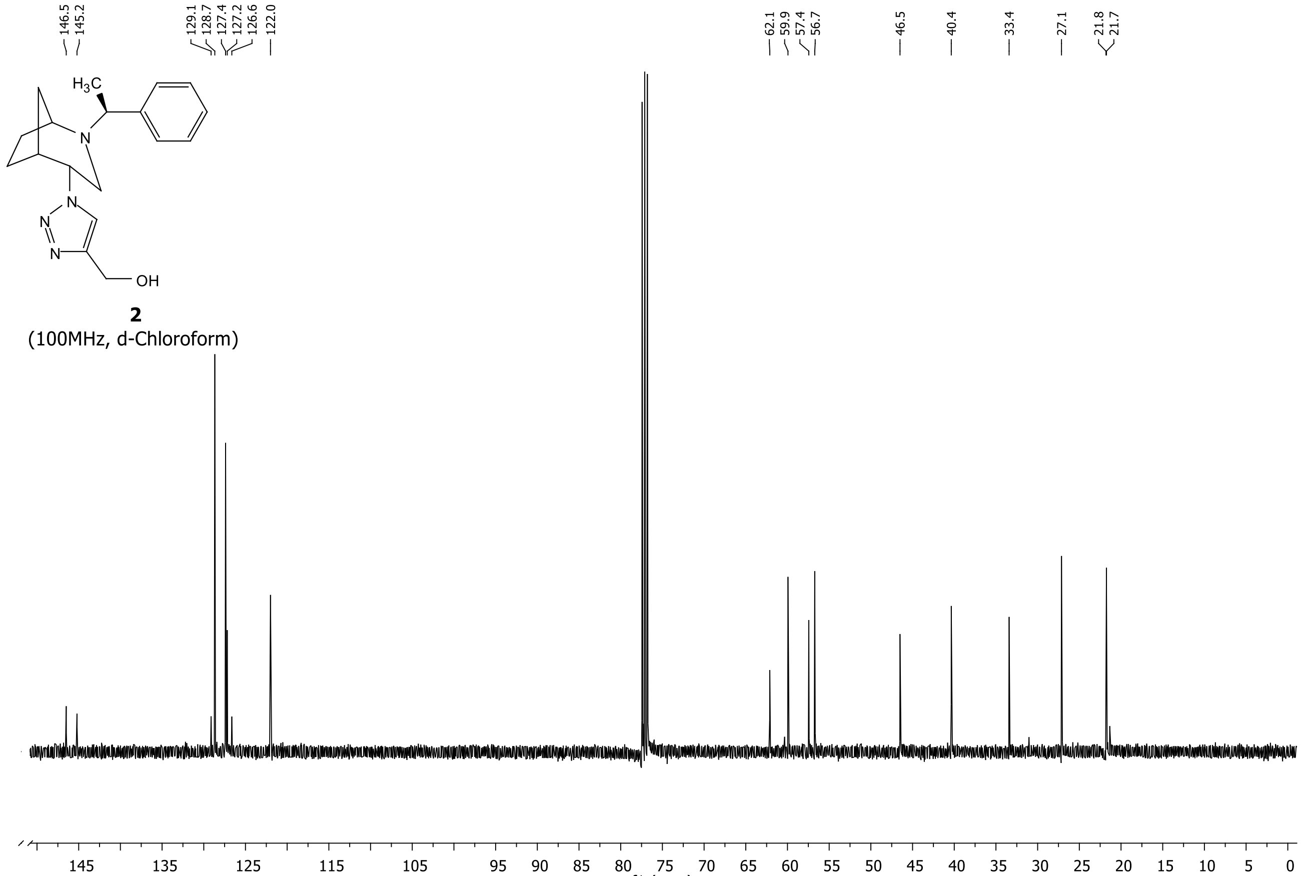

2
(100MHz, d-Chloroform)
f1 (ppm)

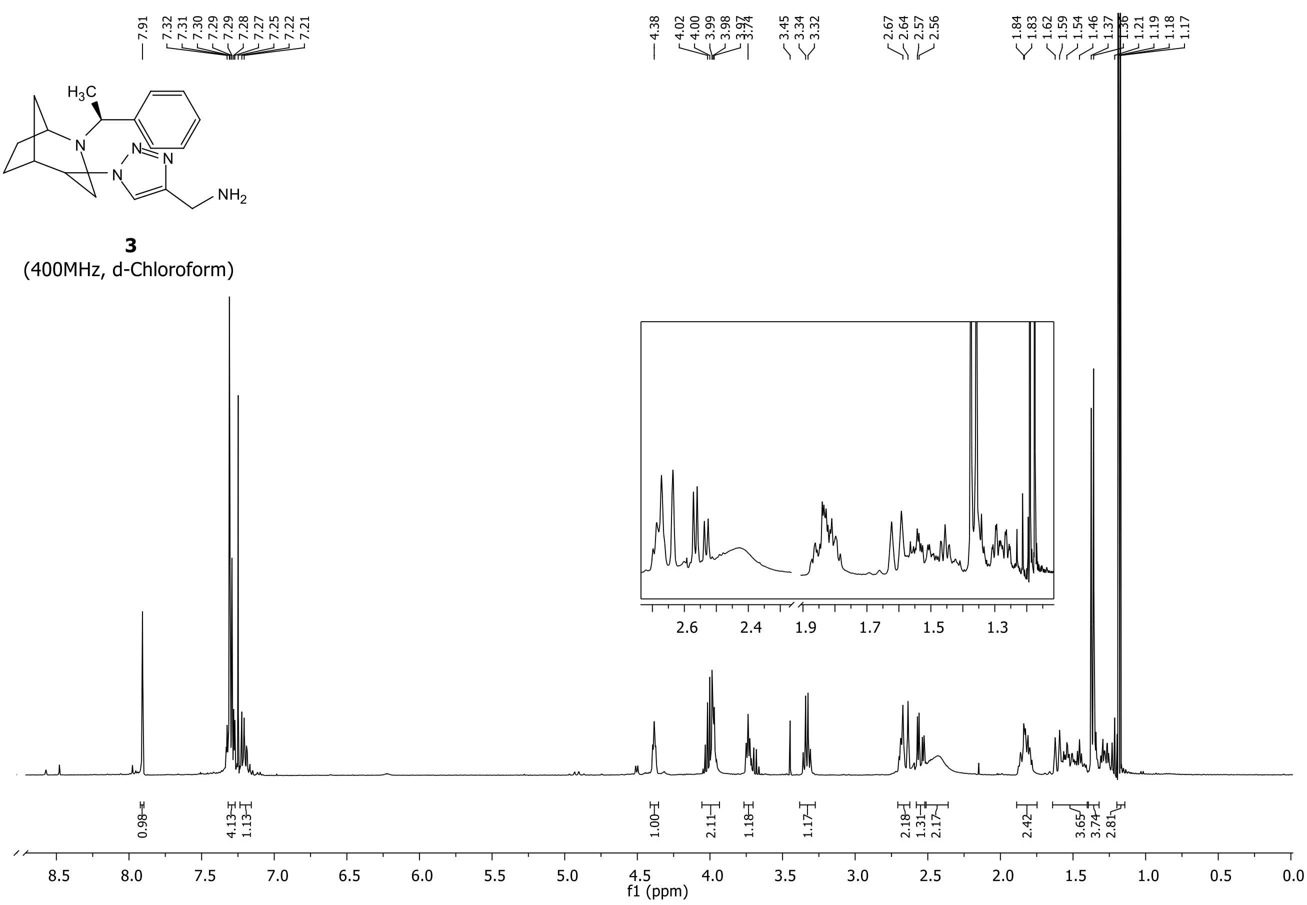
3
(400MHz, d-Chloroform)
f1 (ppm)

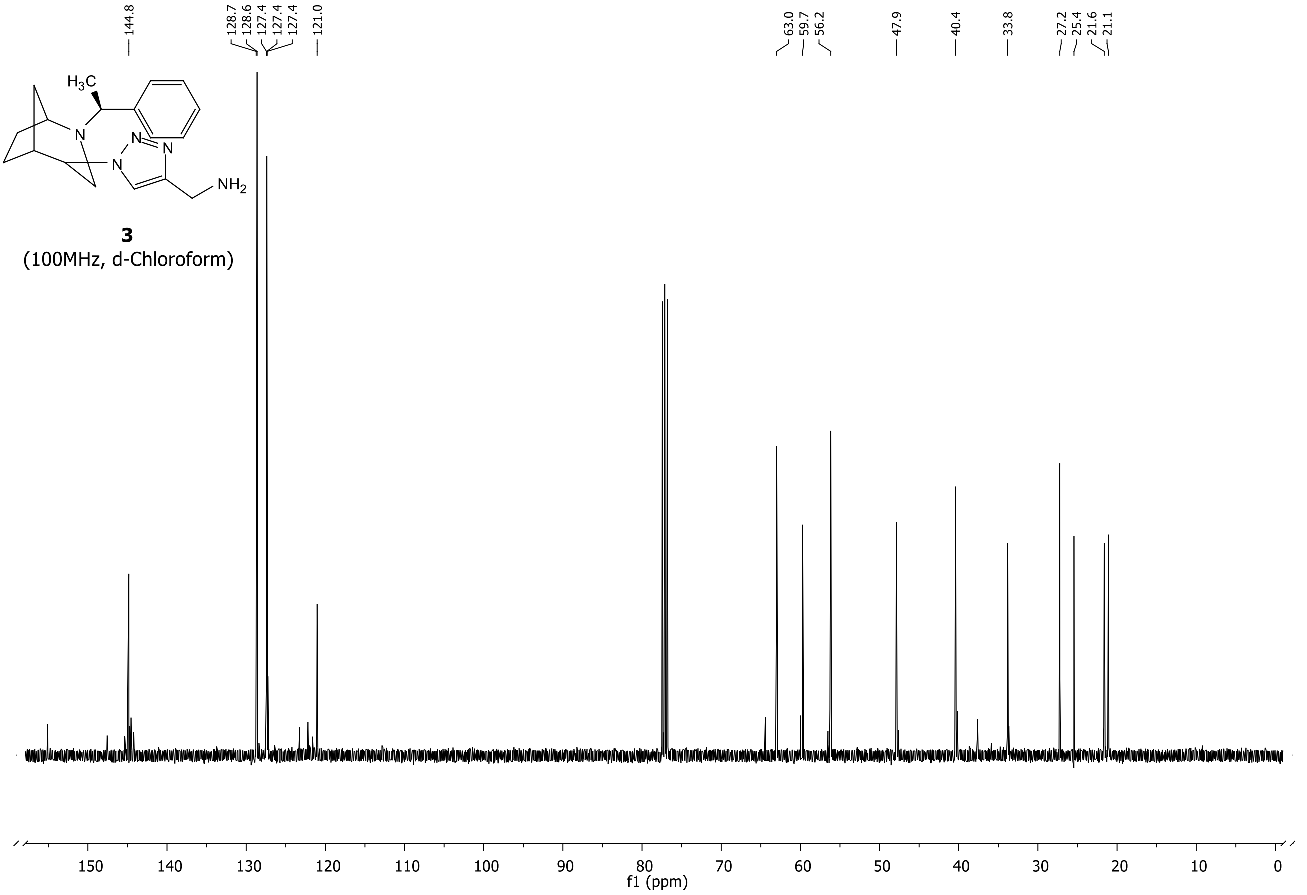
3
(100MHz, d-Chloroform)
f1 (ppm)

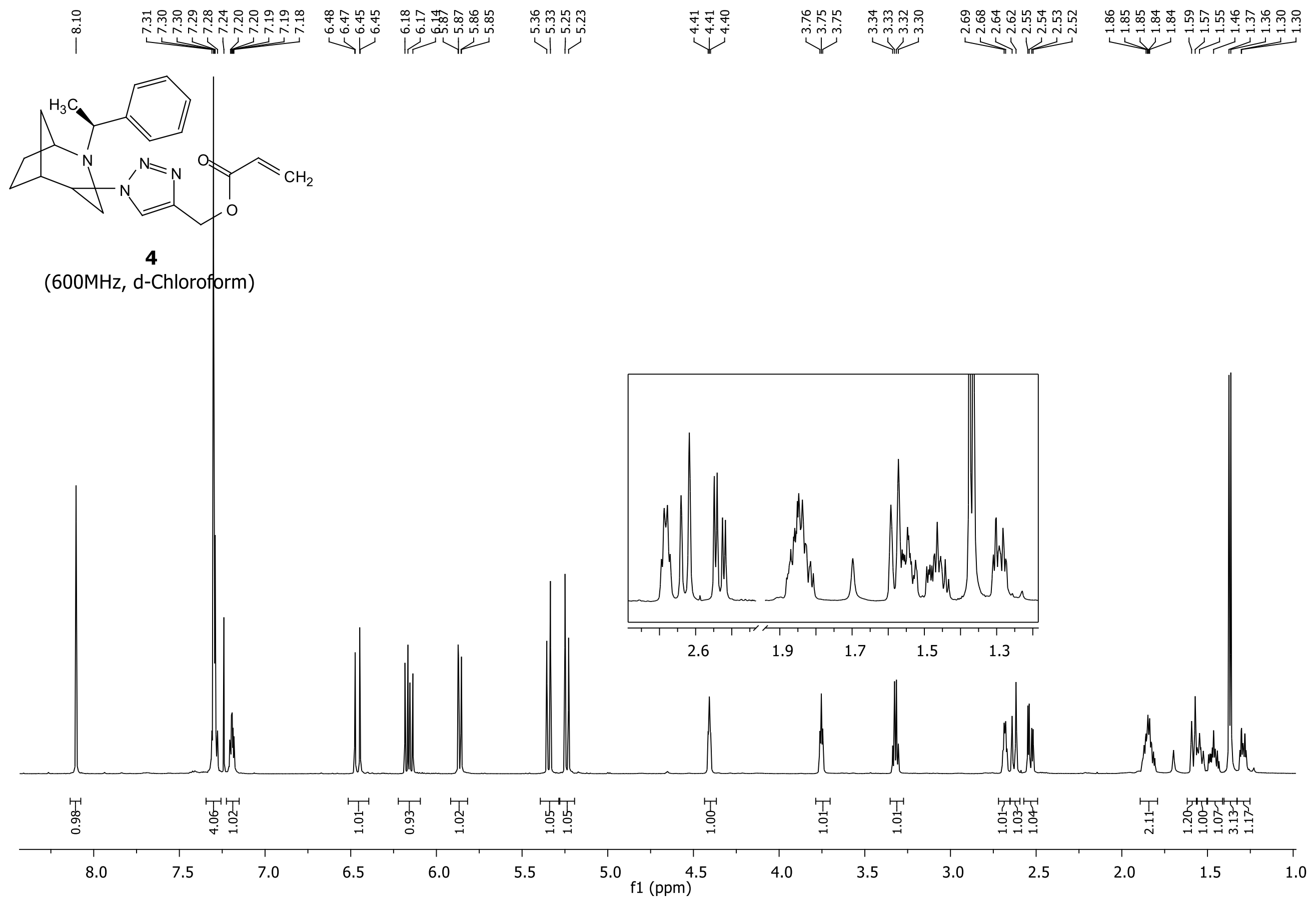

4
(600MHz, d-Chloroform)
f1 (ppm)

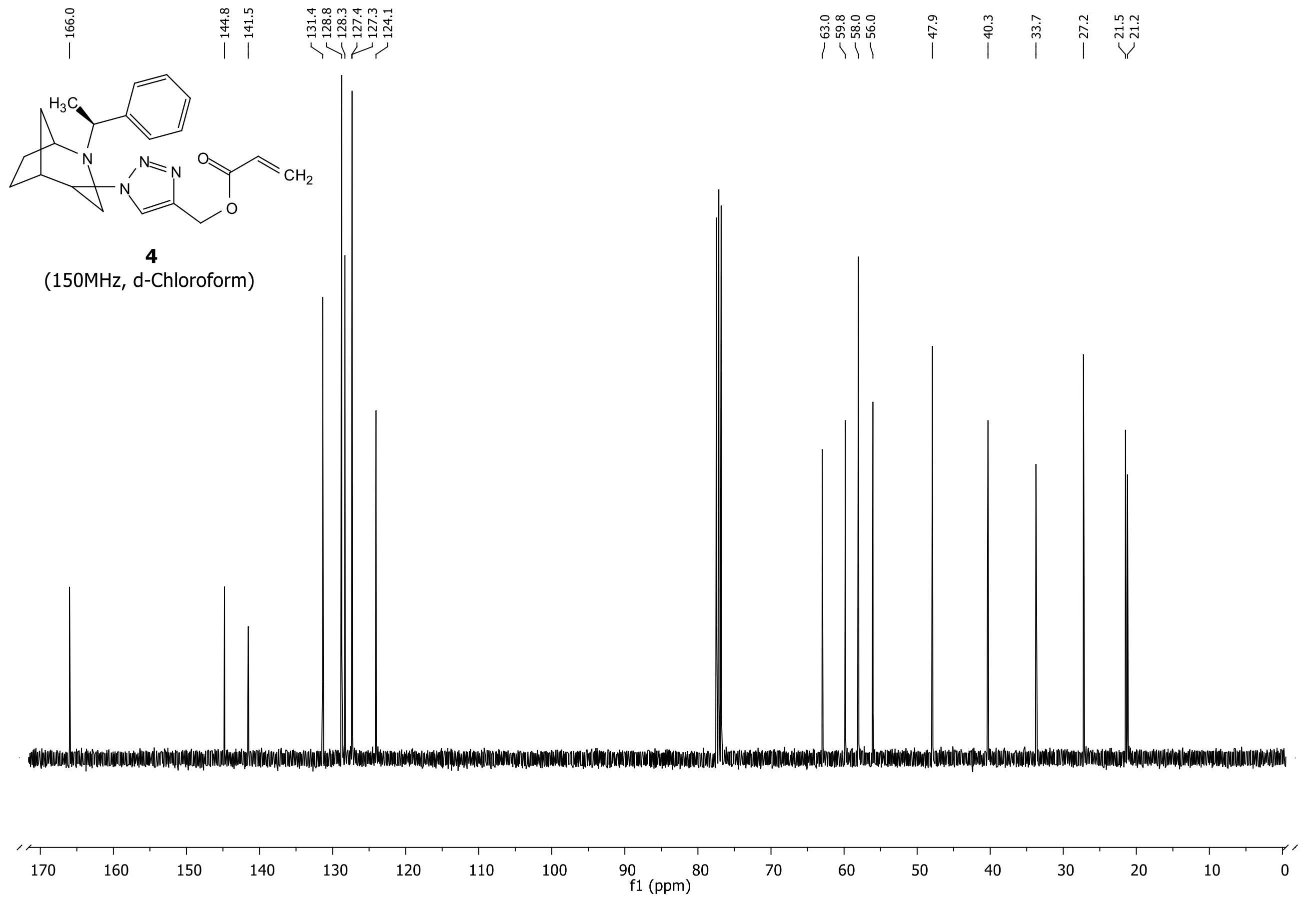

4
(150MHz, d-Chloroform)
f1 (ppm)

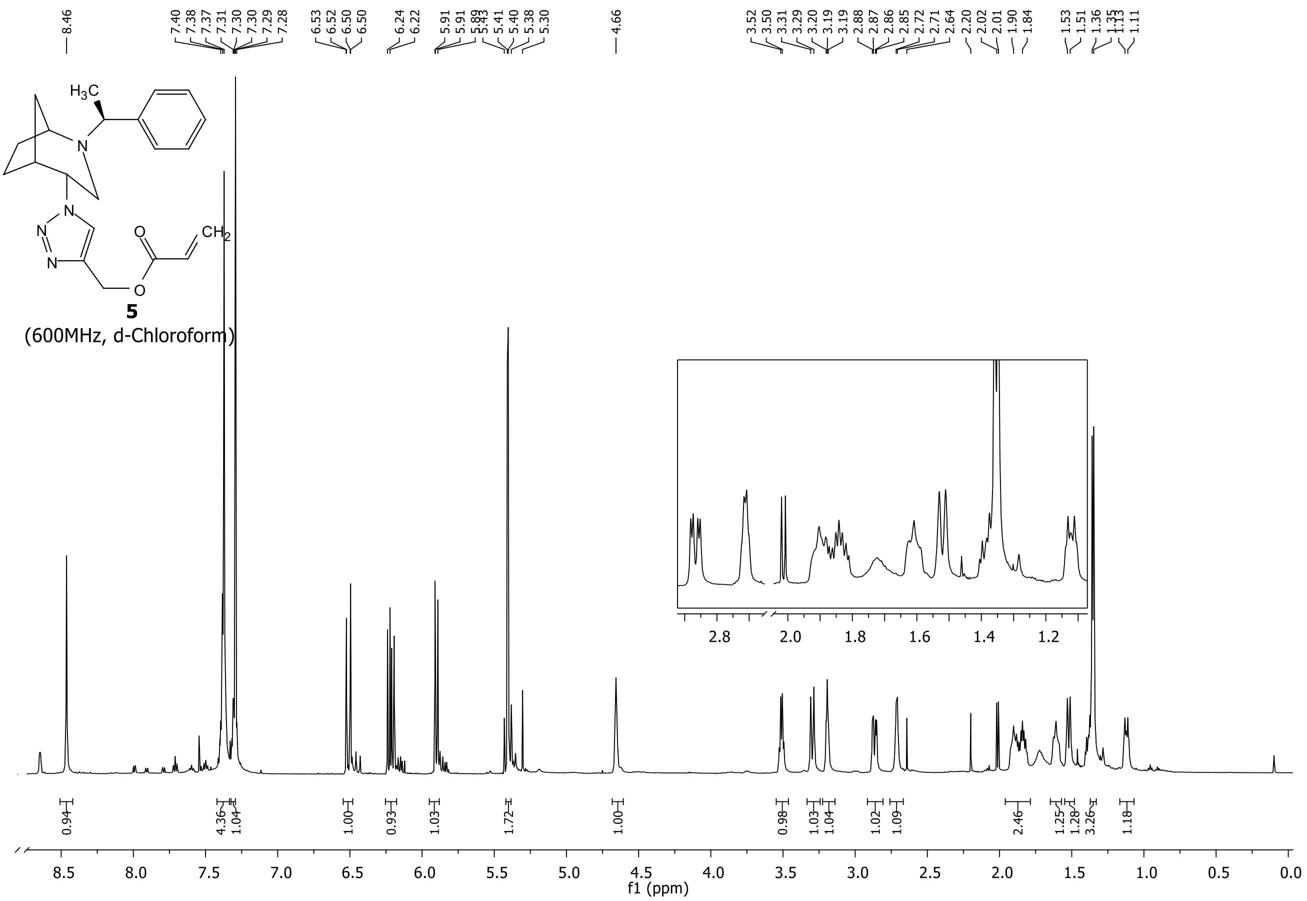
5
(600MHz, d-Chloroform)
f1 (ppm)

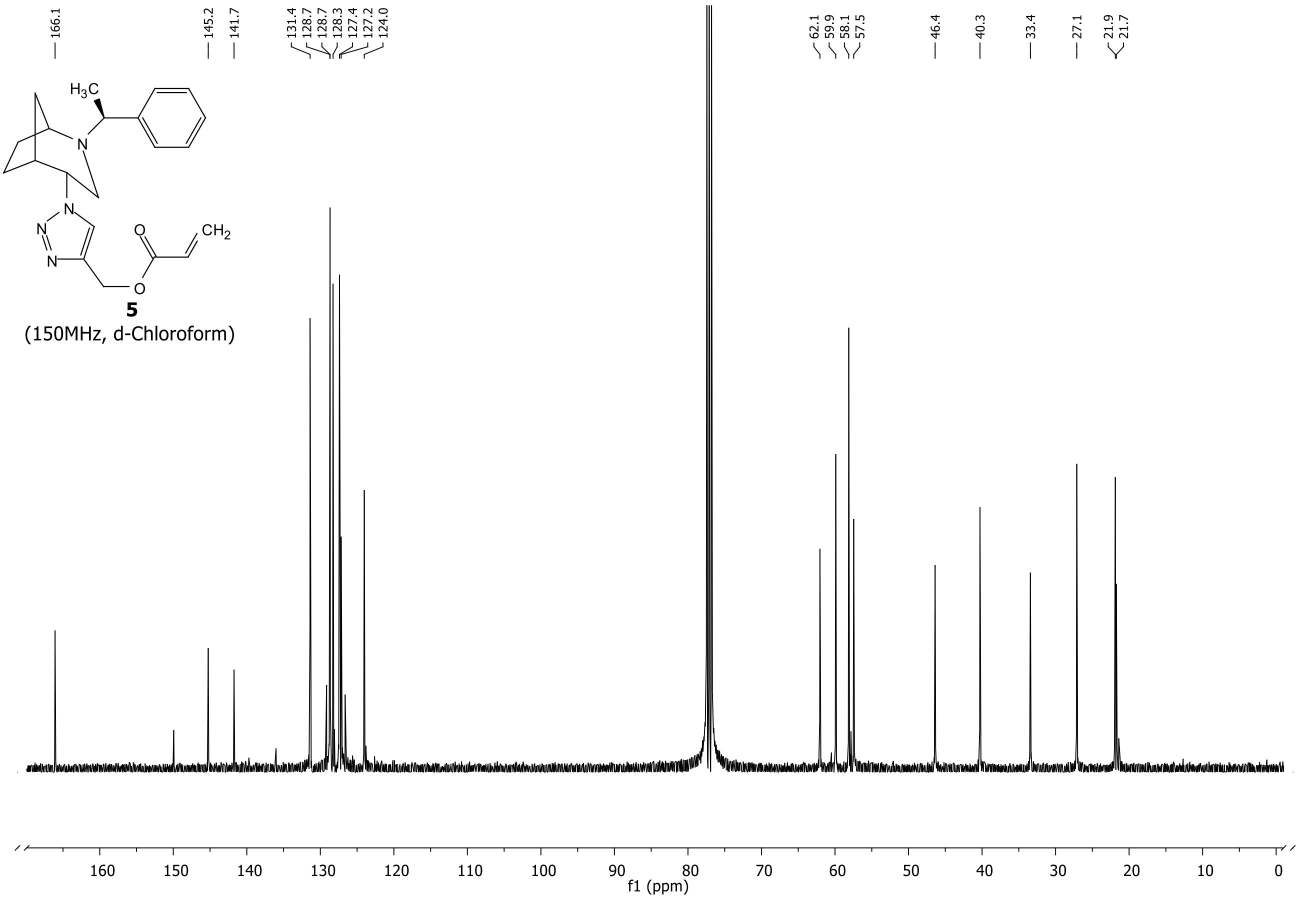
5
(150MHz, d-Chloroform)
f1 (ppm)

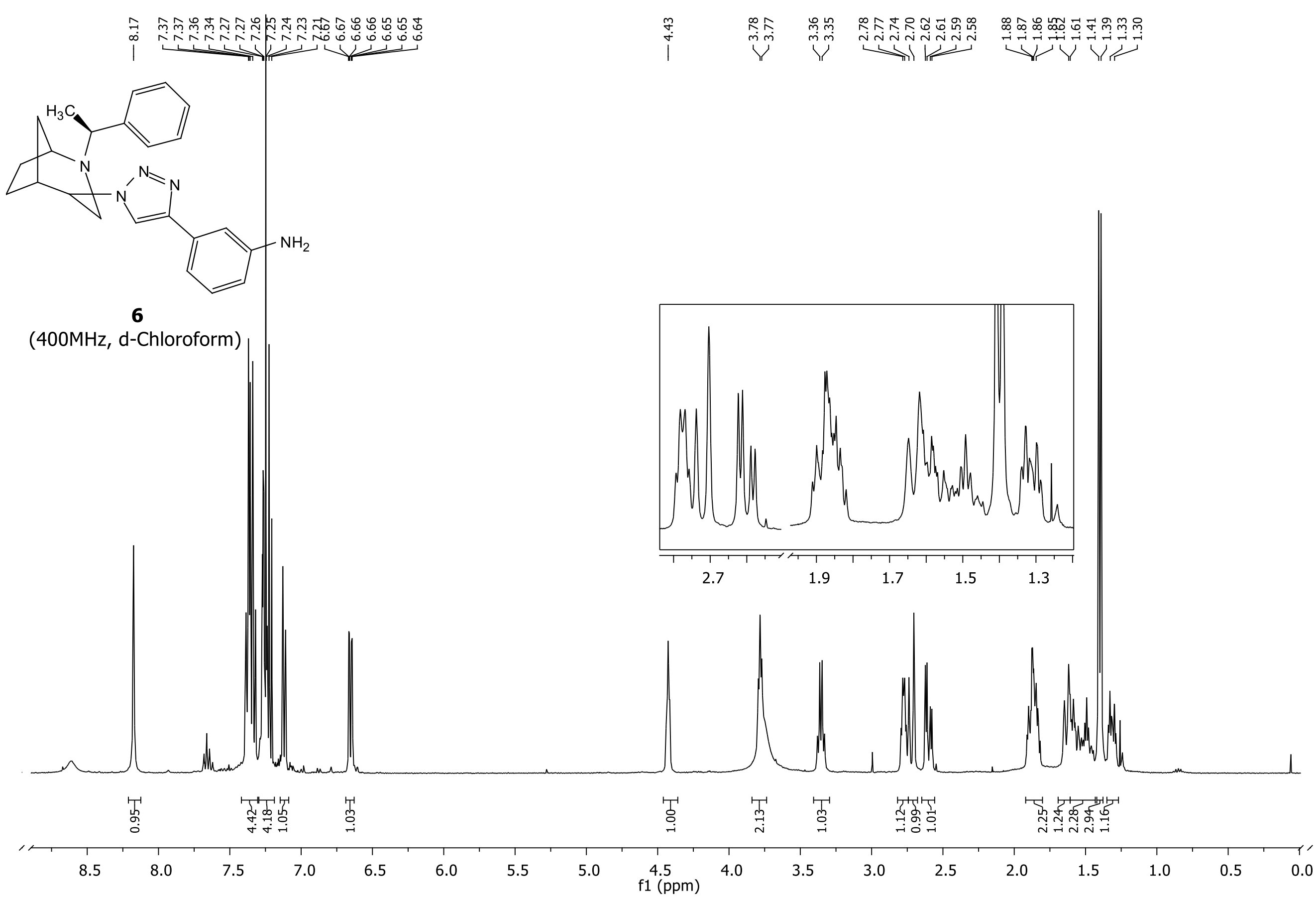
H3C
N
N
N
N
NH2
6
(400MHz, d-Chloroform)
f1 (ppm)

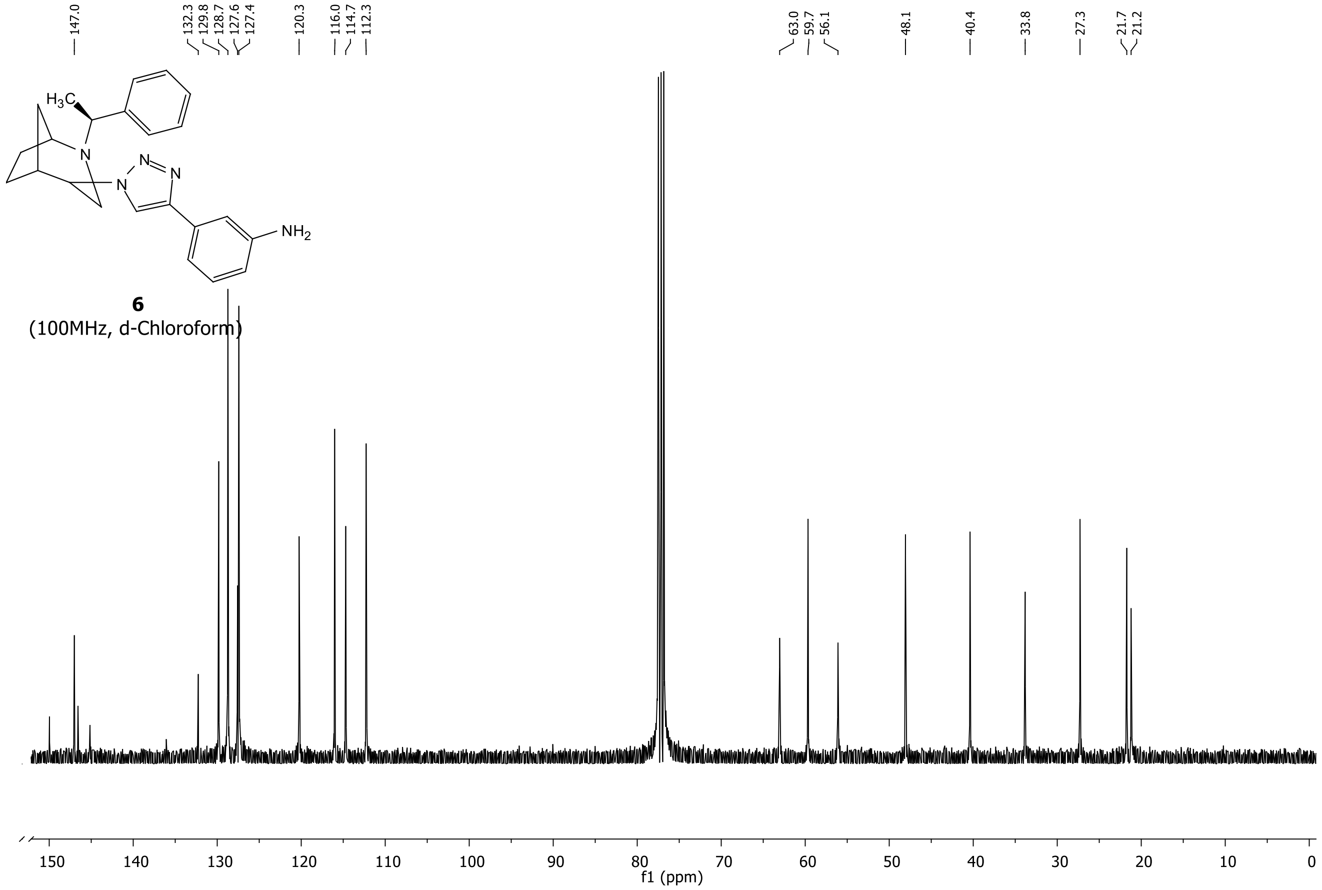
H3C
N
N
N
N
NH2
6
(100MHz, d-Chloroform)
f1 (ppm)

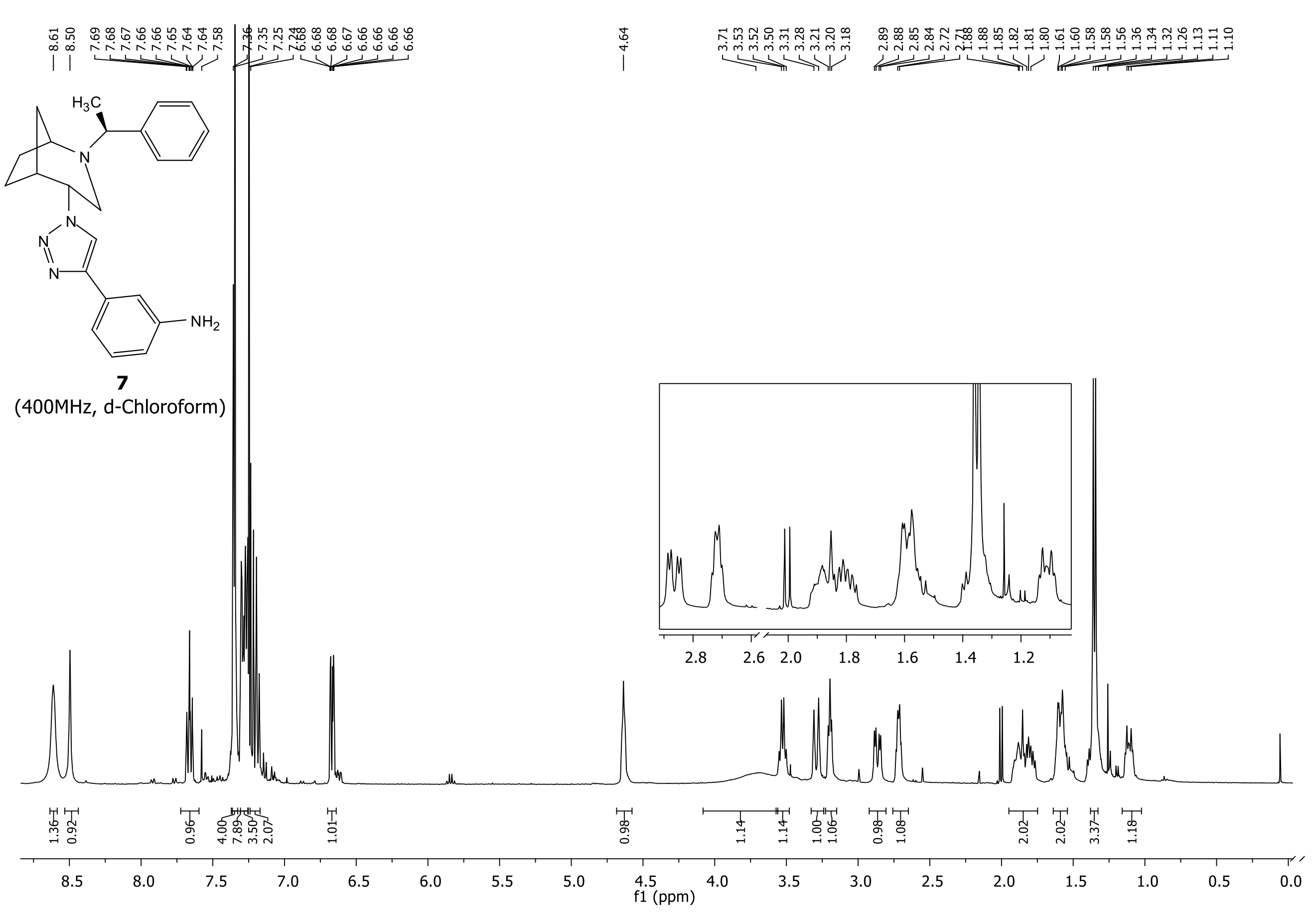
H3C
N
N
N
N
NH2
7
(400MHz, d-Chloroform)
f1 (ppm)

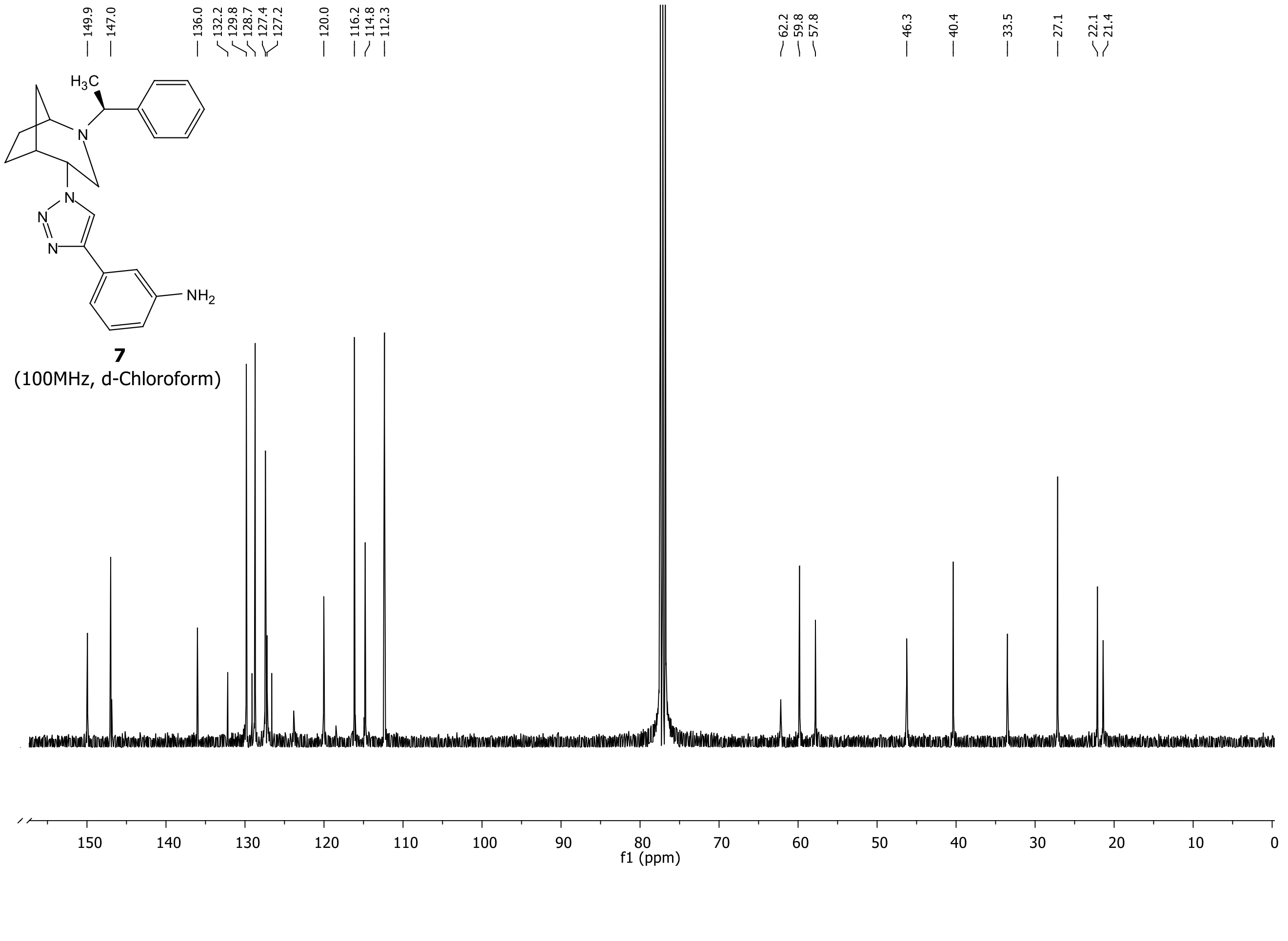
H3C
N
N
N
N
NH2
7
(100MHz, d-Chloroform)
f1 (ppm)

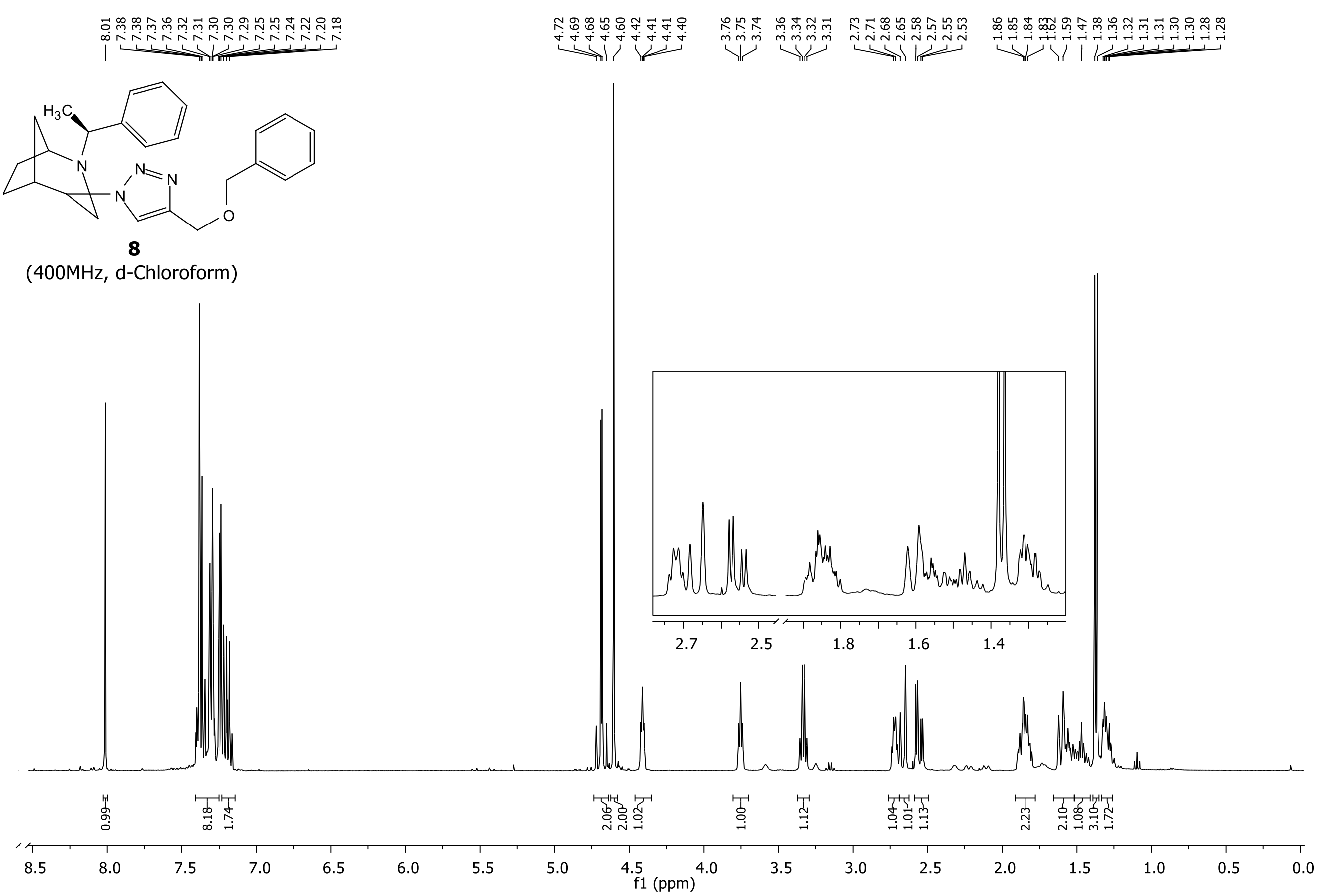
H3C
N
N
N
N
O
8
(400MHz, d-Chloroform)
f1 (ppm)

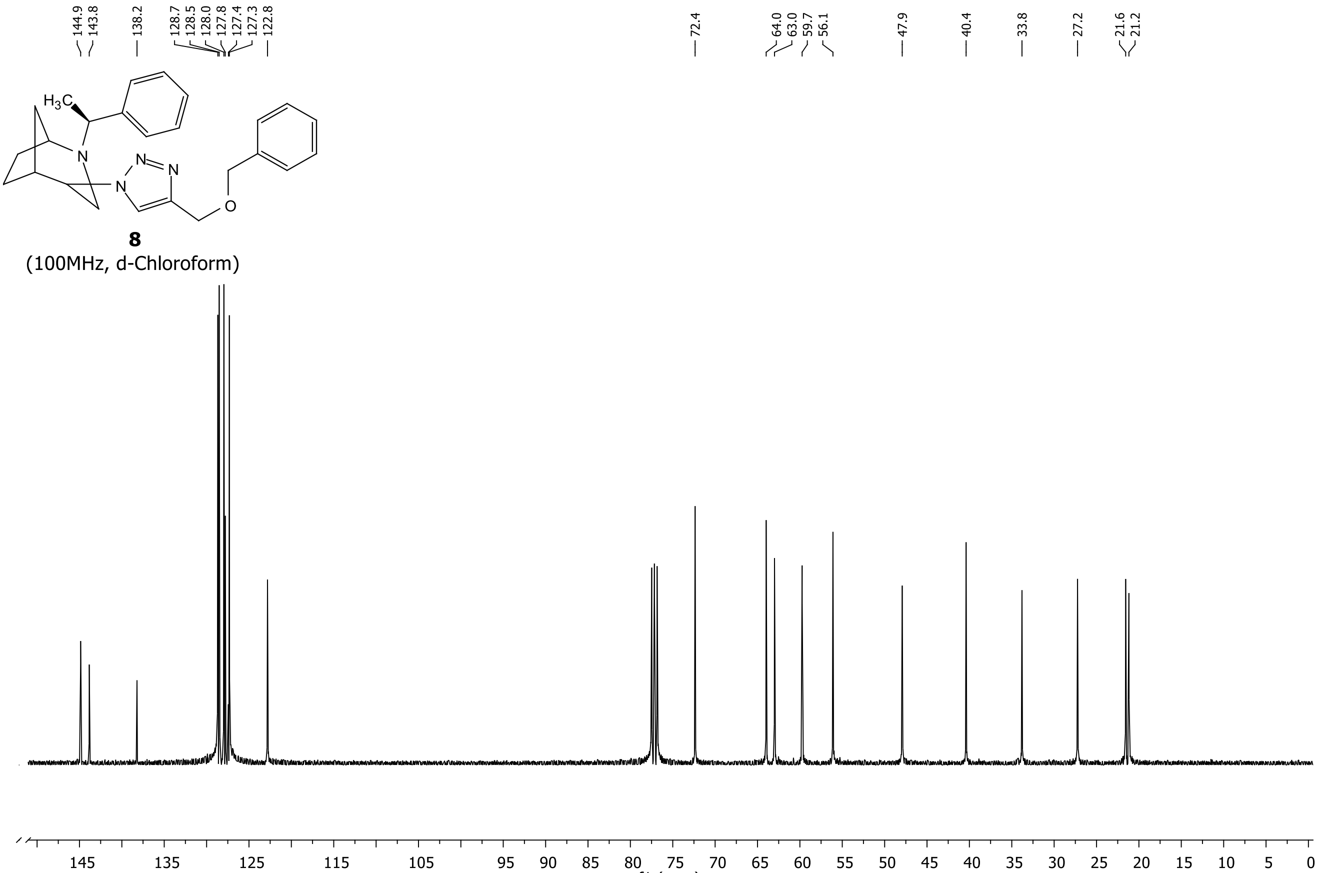
H3C
N
N
N
N
O
8
(100MHz, d-Chloroform)
f1 (ppm)

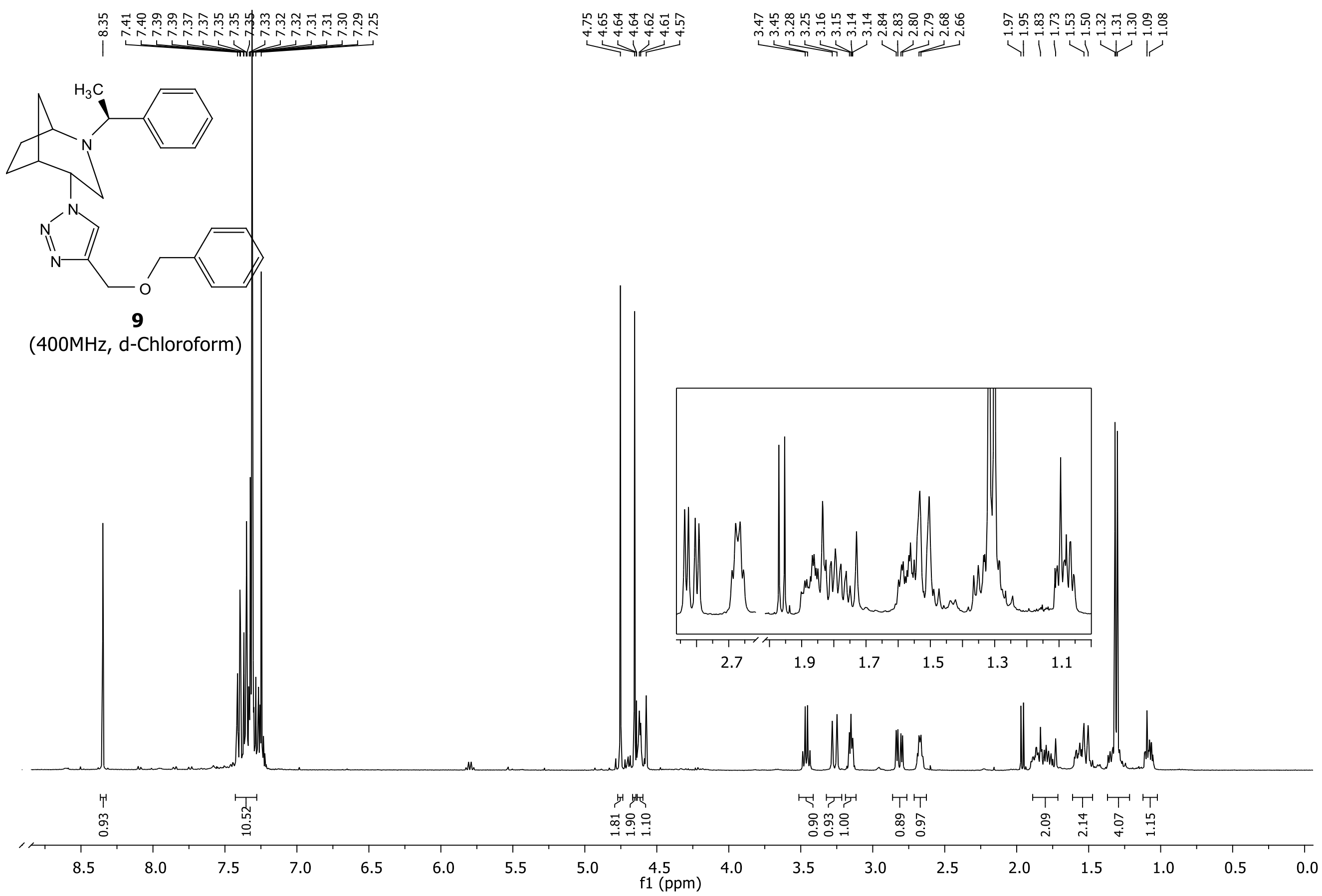
9
(400MHz, d-Chloroform)
f1 (ppm)

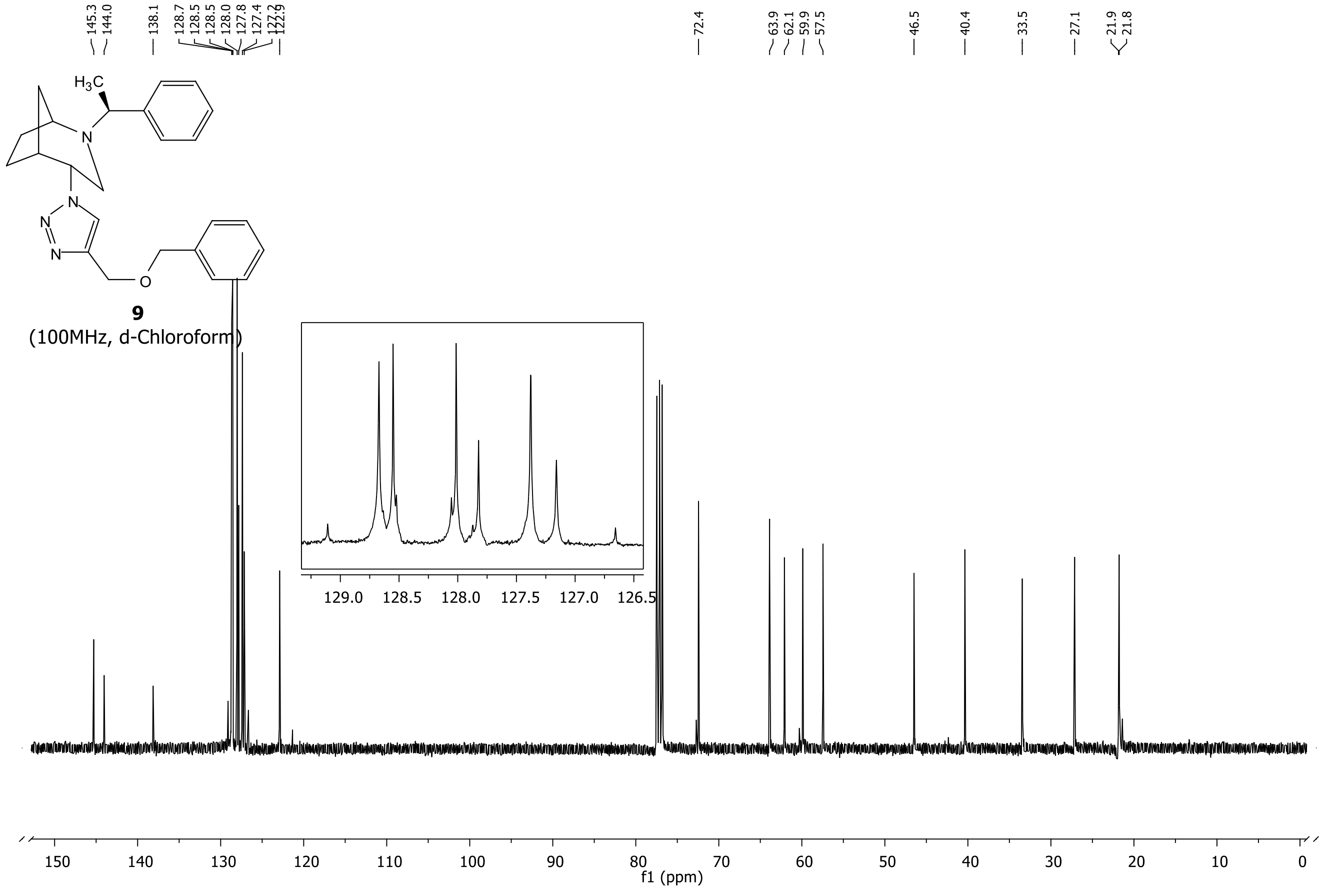
9
(100MHz, d-Chloroform)
f1 (ppm)

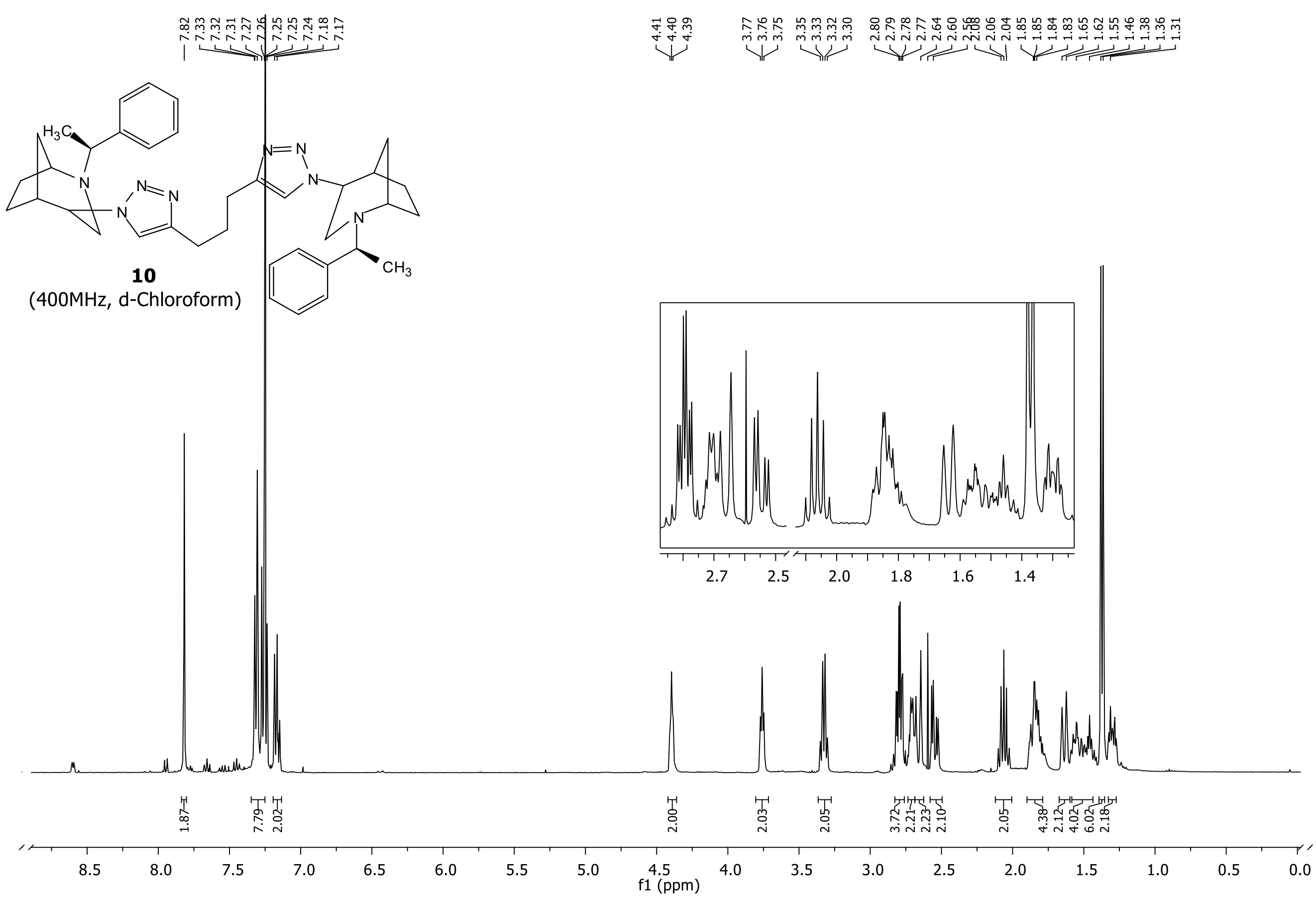

10
(400MHz, d-Chloroform)
f1 (ppm)

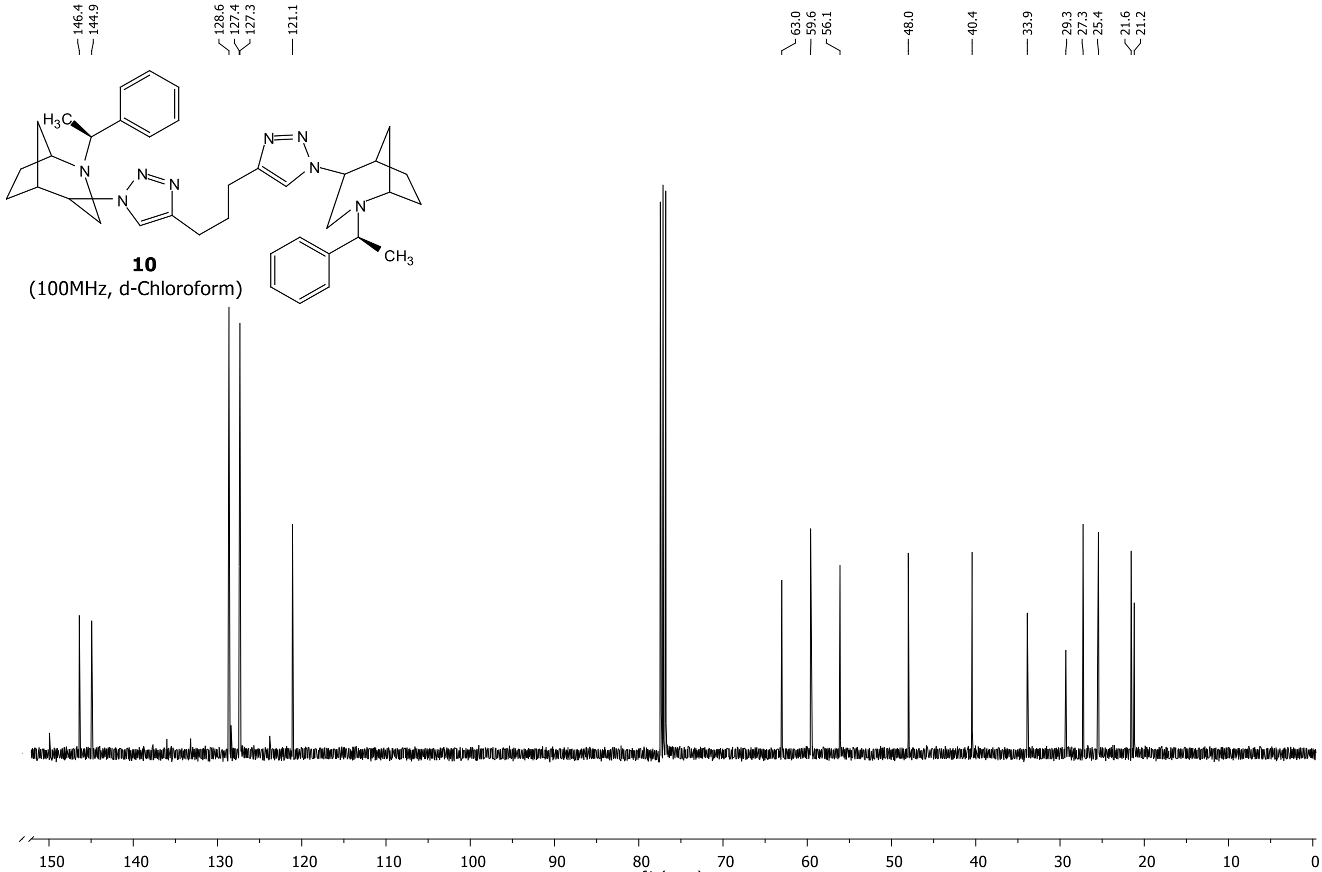

10
(100MHz, d-Chloroform)
f1 (ppm)

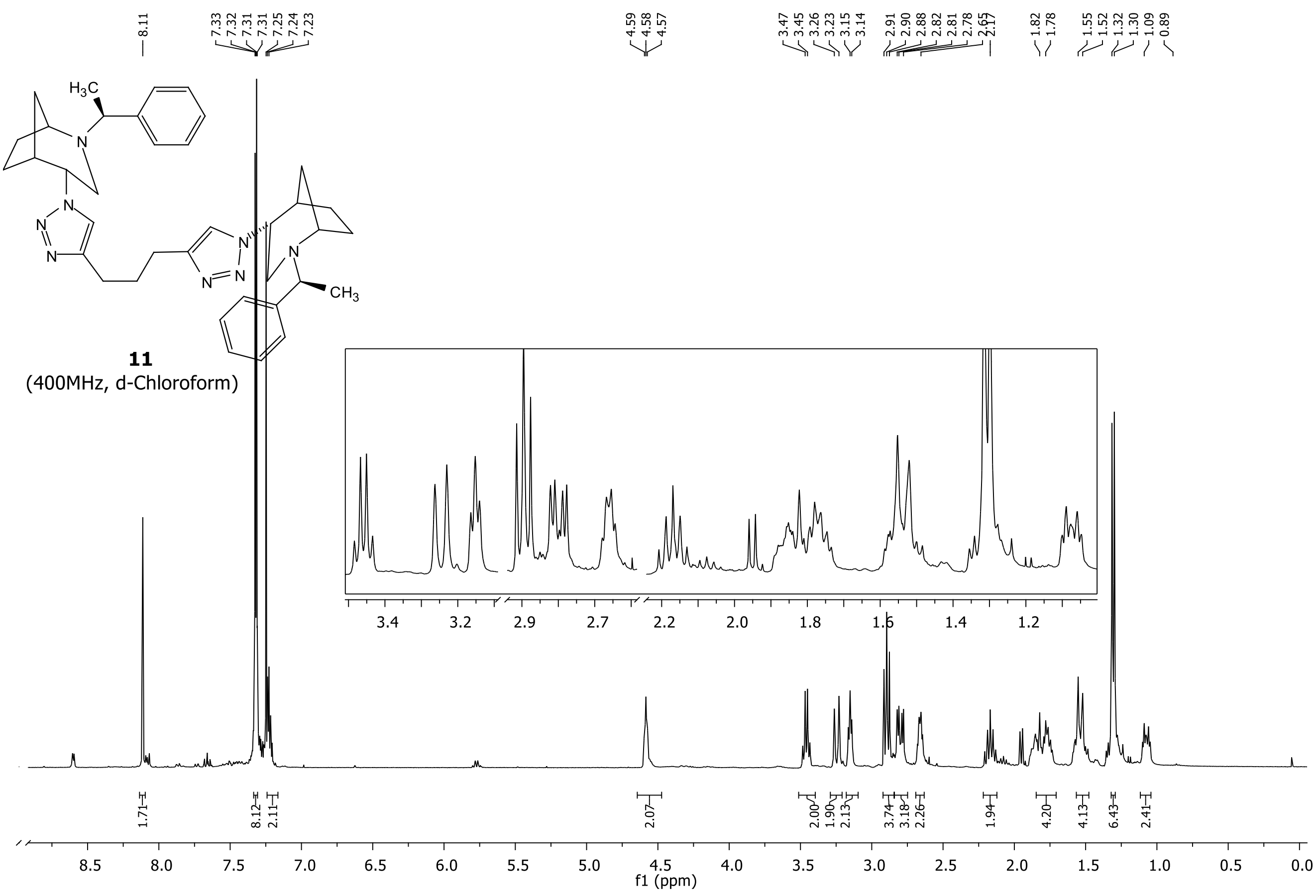

11
(400MHz, d-Chloroform)
f1 (ppm)

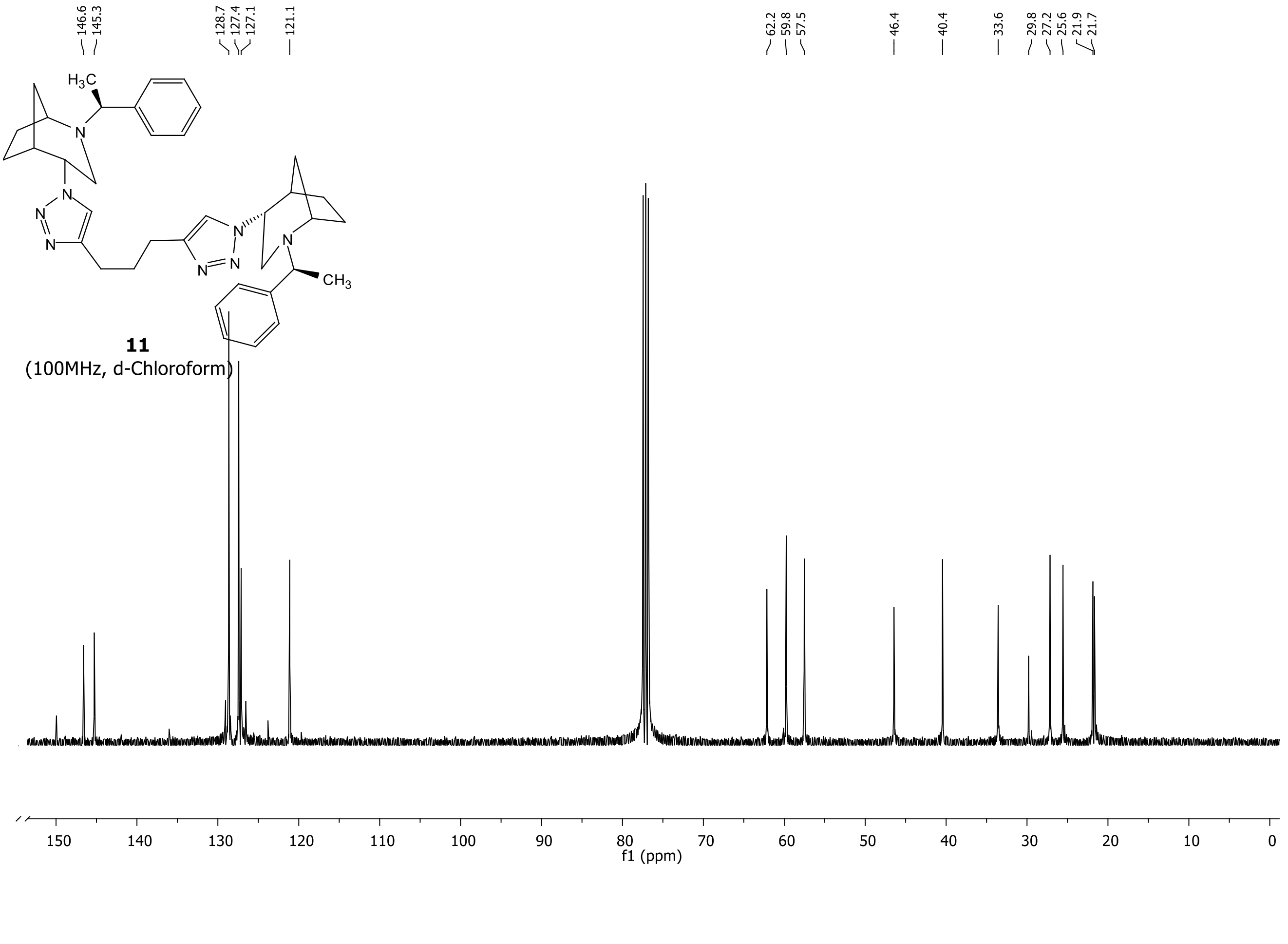

11
(100MHz, d-Chloroform)
f1 (ppm)

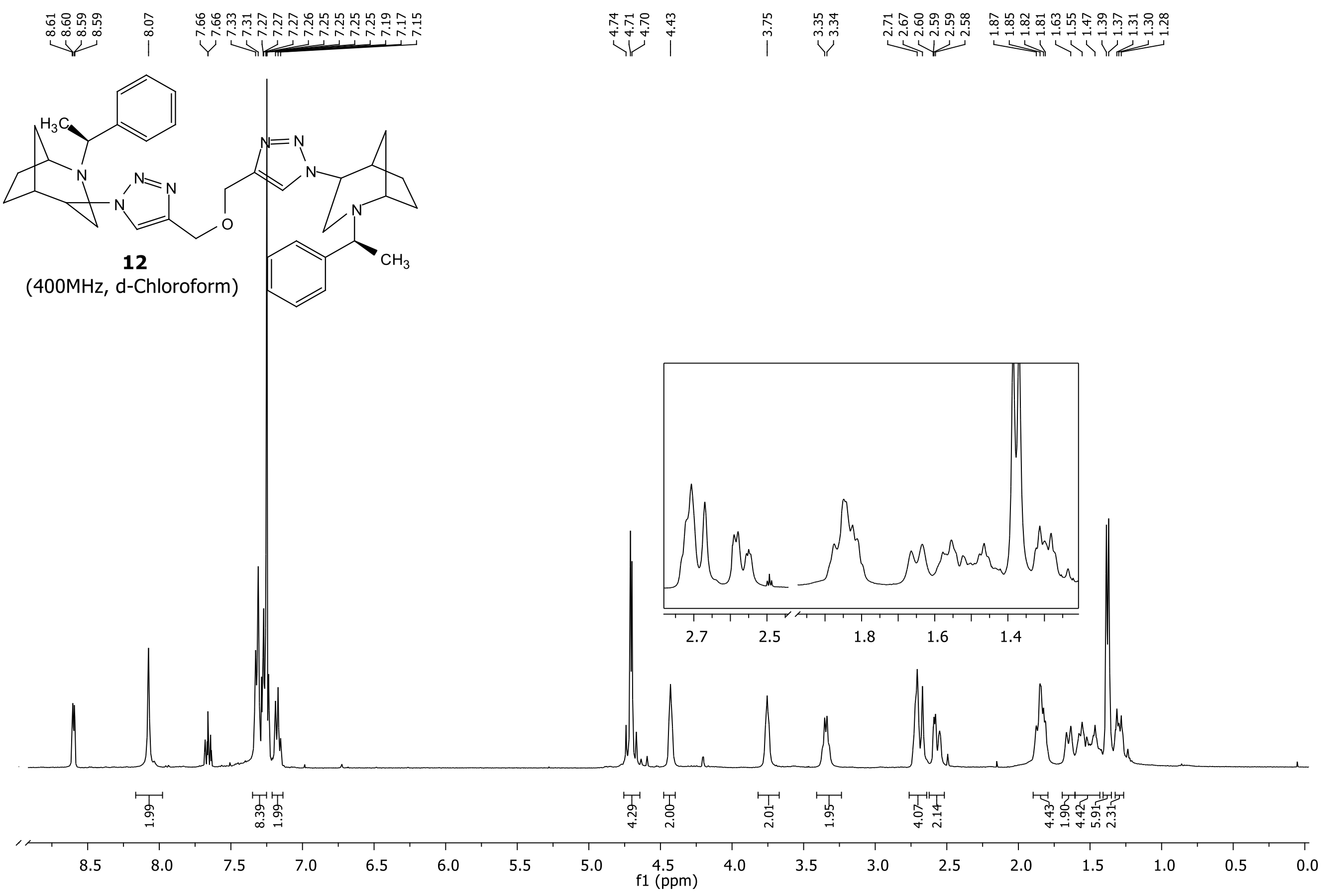
12
(400MHz, d-Chloroform)
8.61
8.60
8.59
8.59
8.07
7.66
7.66
7.33
7.31
7.27
7.27
7.27
7.26
7.25
7.25
7.25
7.25
7.19
7.17
7.15
4.74
4.71
4.70
4.43
3.75
3.35
3.34
2.71
2.67
2.60
2.59
2.59
2.58
1.87
1.85
1.82
1.81
1.63
1.55
1.47
1.39
1.37
1.31
1.30
1.28
1.99
8.39
1.99
4.29
2.00
2.01
1.95
4.07
2.14
4.43
1.90
4.42
5.91
2.31
f1 (ppm)

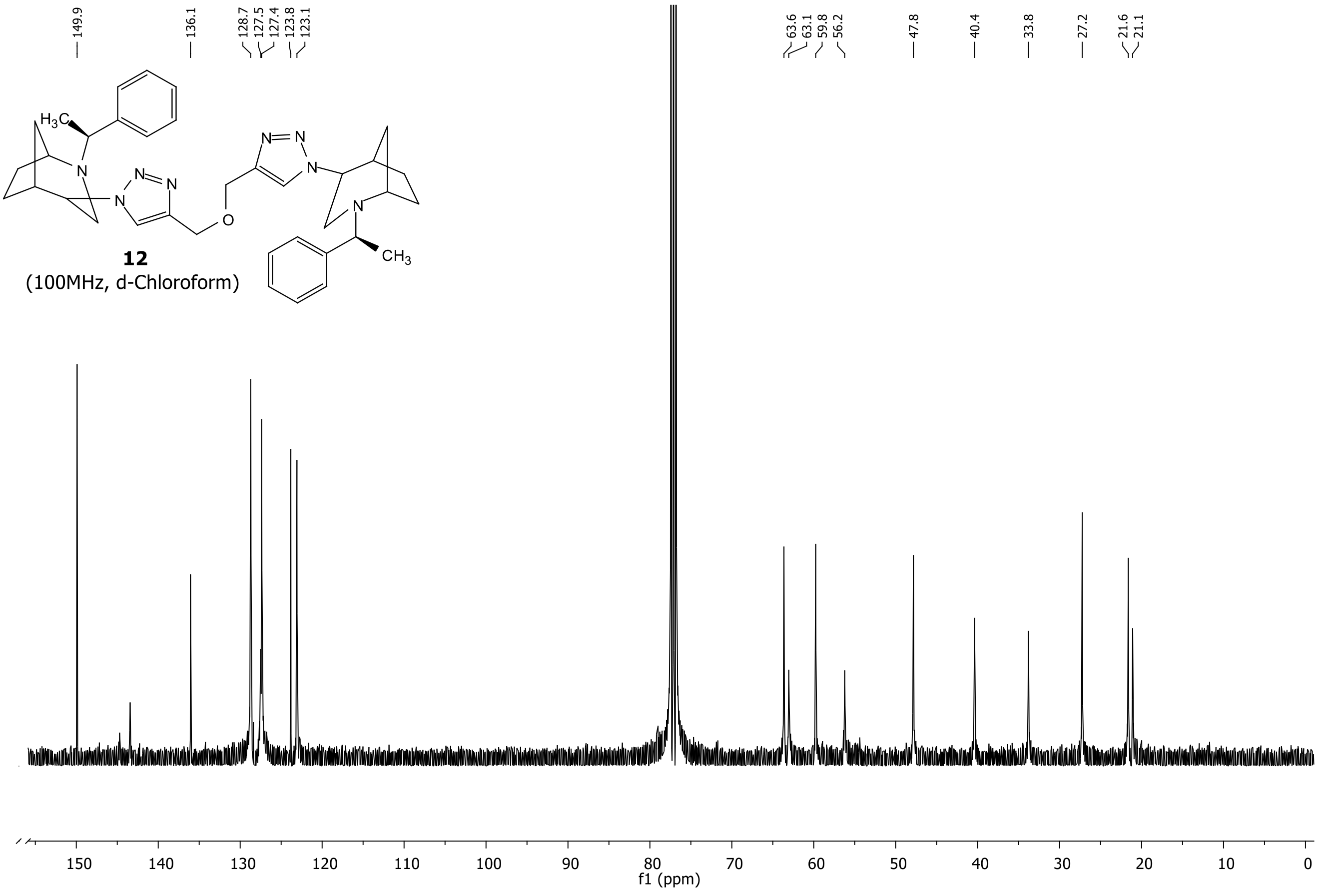
12
(100MHz, d-Chloroform)
149.9
136.1
128.7
127.5
127.4
123.8
123.1
63.6
63.1
59.8
56.2
47.8
40.4
33.8
27.2
21.6
21.1
f1 (ppm)

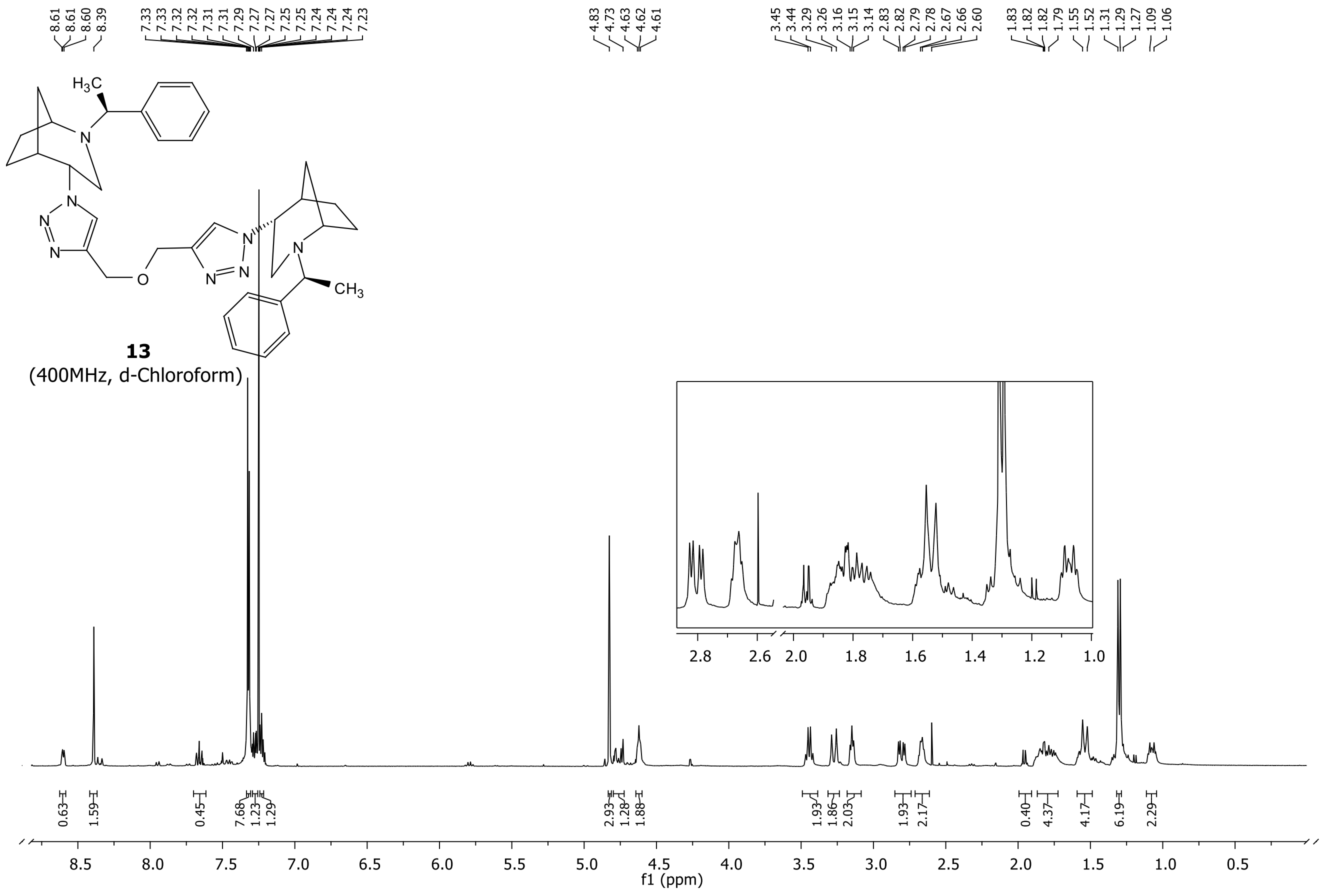
13
(400MHz, d-Chloroform)
f1 (ppm)

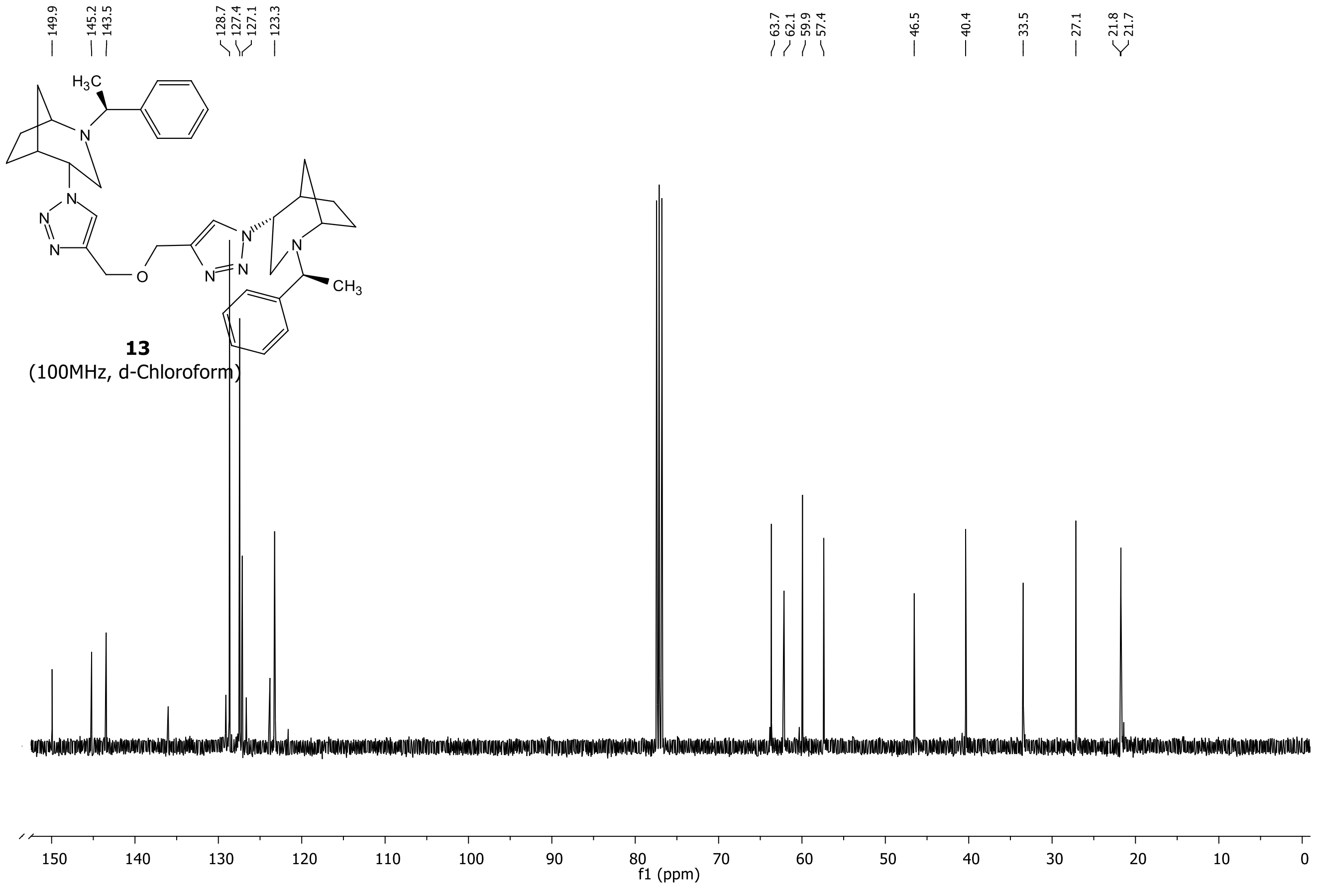
13
(100MHz, d-Chloroform)
f1 (ppm)

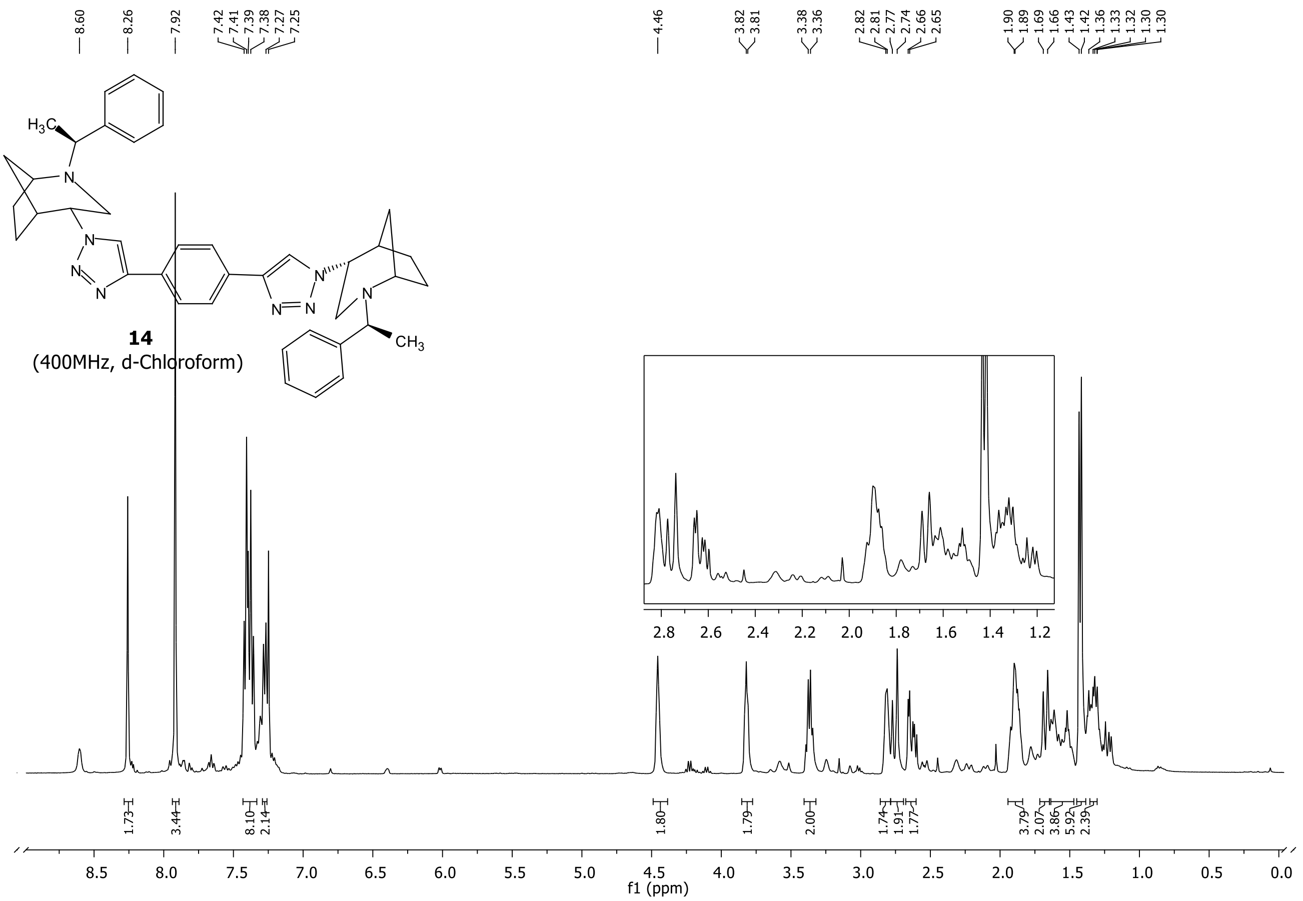
14
(400MHz, d-Chloroform)
f1 (ppm)

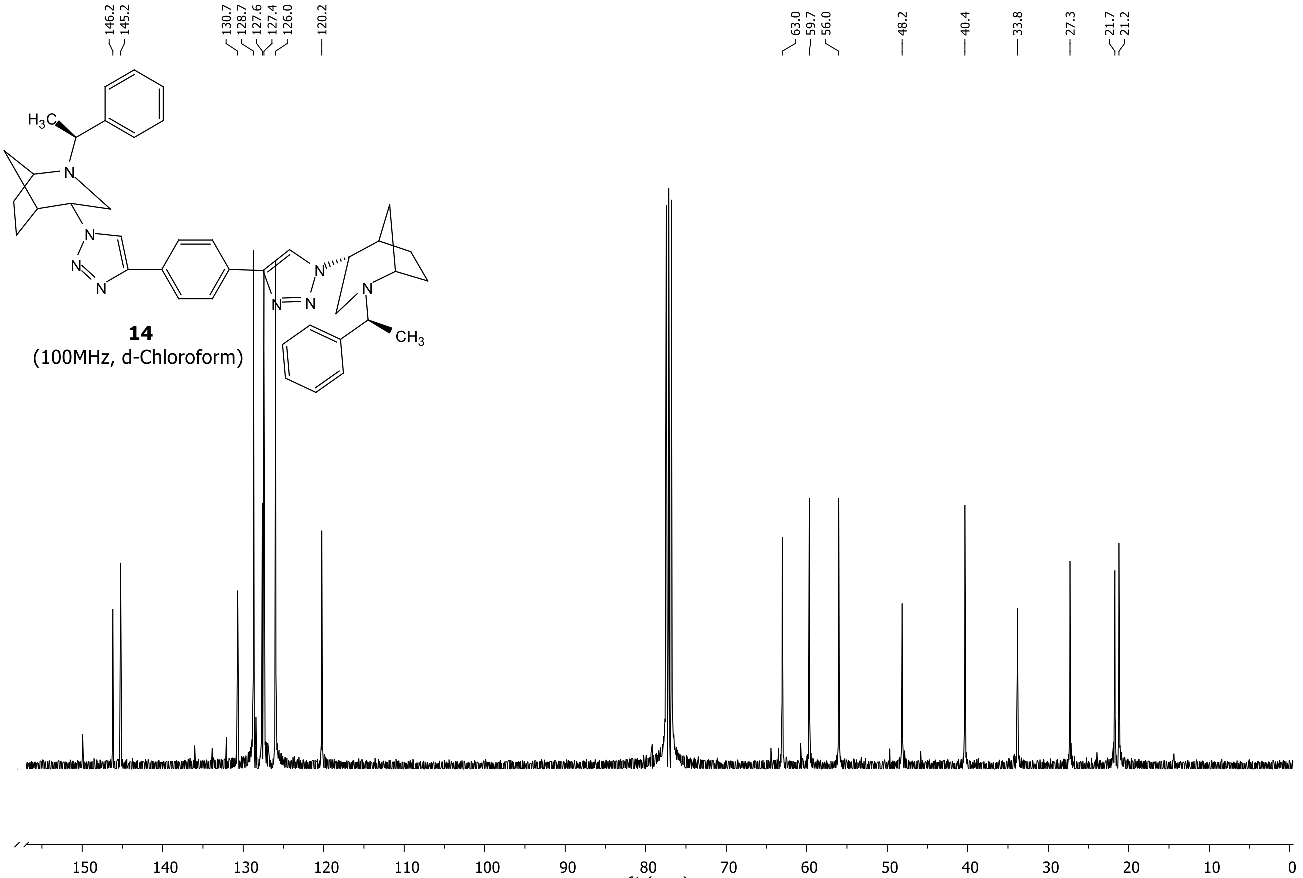
14
(100MHz, d-Chloroform)
f1 (ppm)

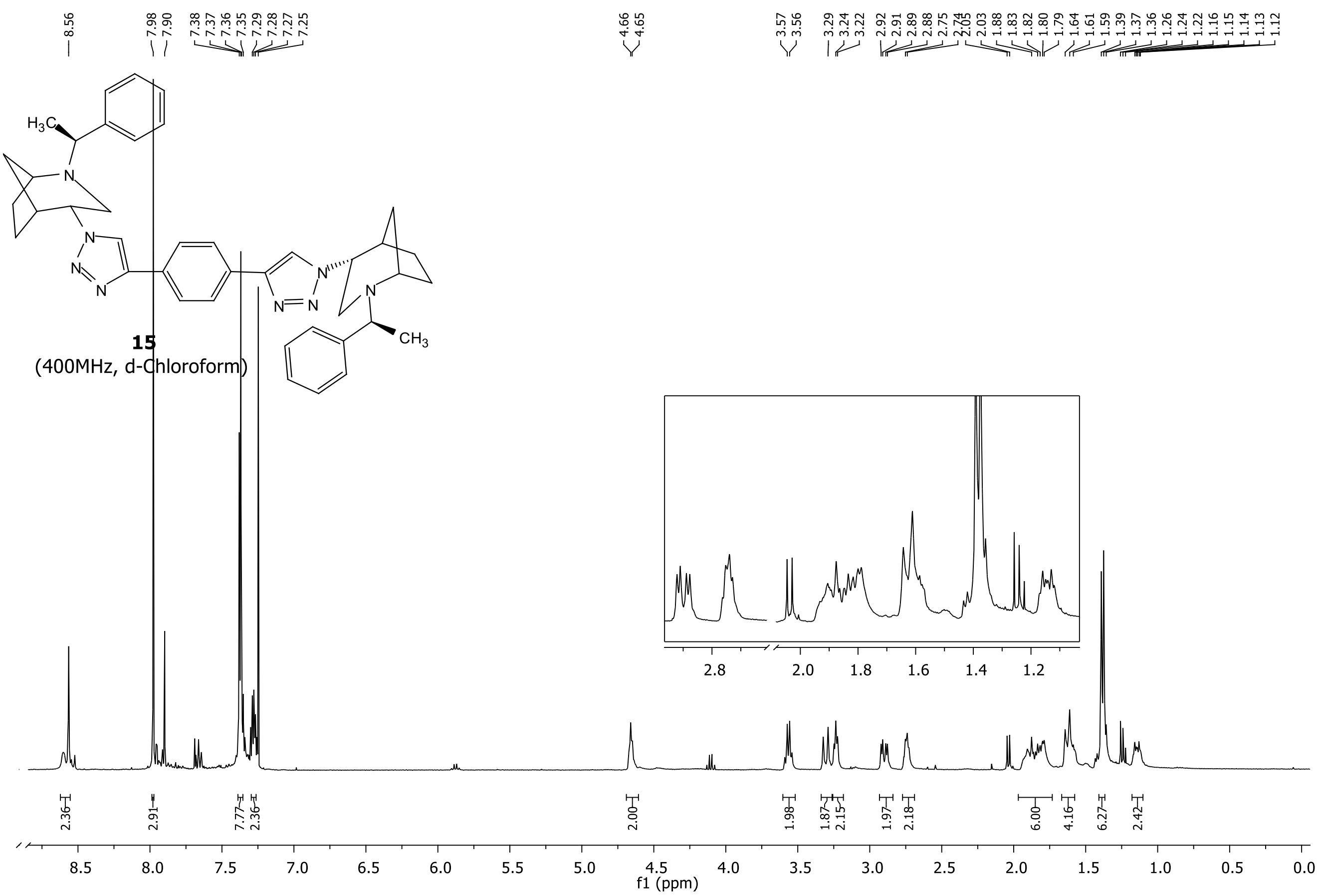

8.56
7.98
7.90
7.38
7.37
7.36
7.35
7.29
7.28
7.27
7.25
4.66
4.65
3.57
3.56
3.29
3.24
3.22
2.92
2.91
2.89
2.88
2.75
2.74
2.05
2.03
1.88
1.83
1.82
1.80
1.79
1.64
1.61
1.59
1.39
1.37
1.36
1.26
1.24
1.22
1.16
1.15
1.14
1.13
1.12
15
(400MHz, d-Chloroform)
2.36
2.91
7.77
2.36
2.00
1.98
1.87
2.15
1.97
2.18
6.00
4.16
6.27
2.42
f1 (ppm)

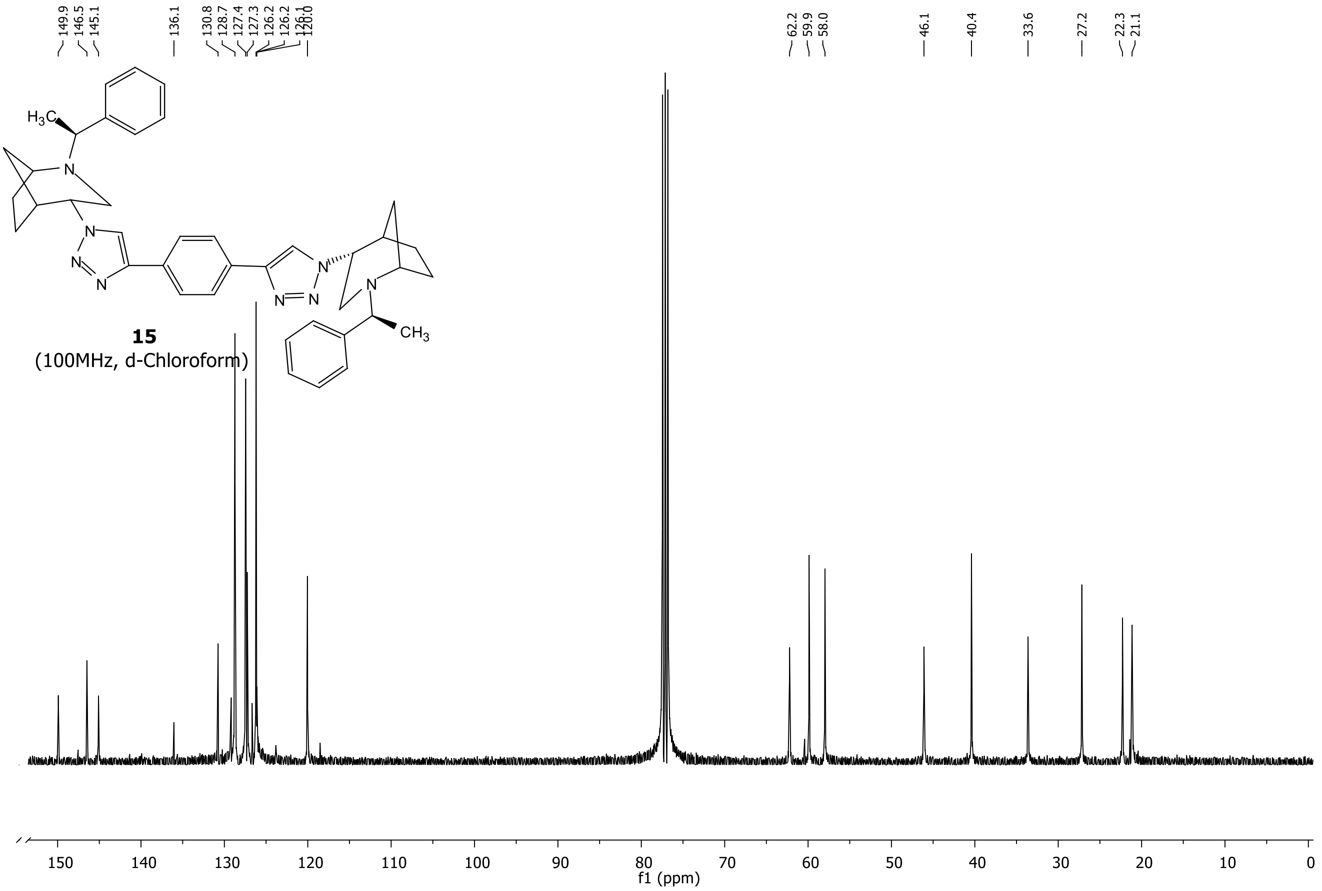

149.9
146.5
145.1
136.1
130.8
128.7
127.4
127.3
126.2
126.2
126.1
120.0
62.2
59.9
58.0
46.1
40.4
33.6
27.2
22.3
21.1
15
(100MHz, d-Chloroform)
f1 (ppm)

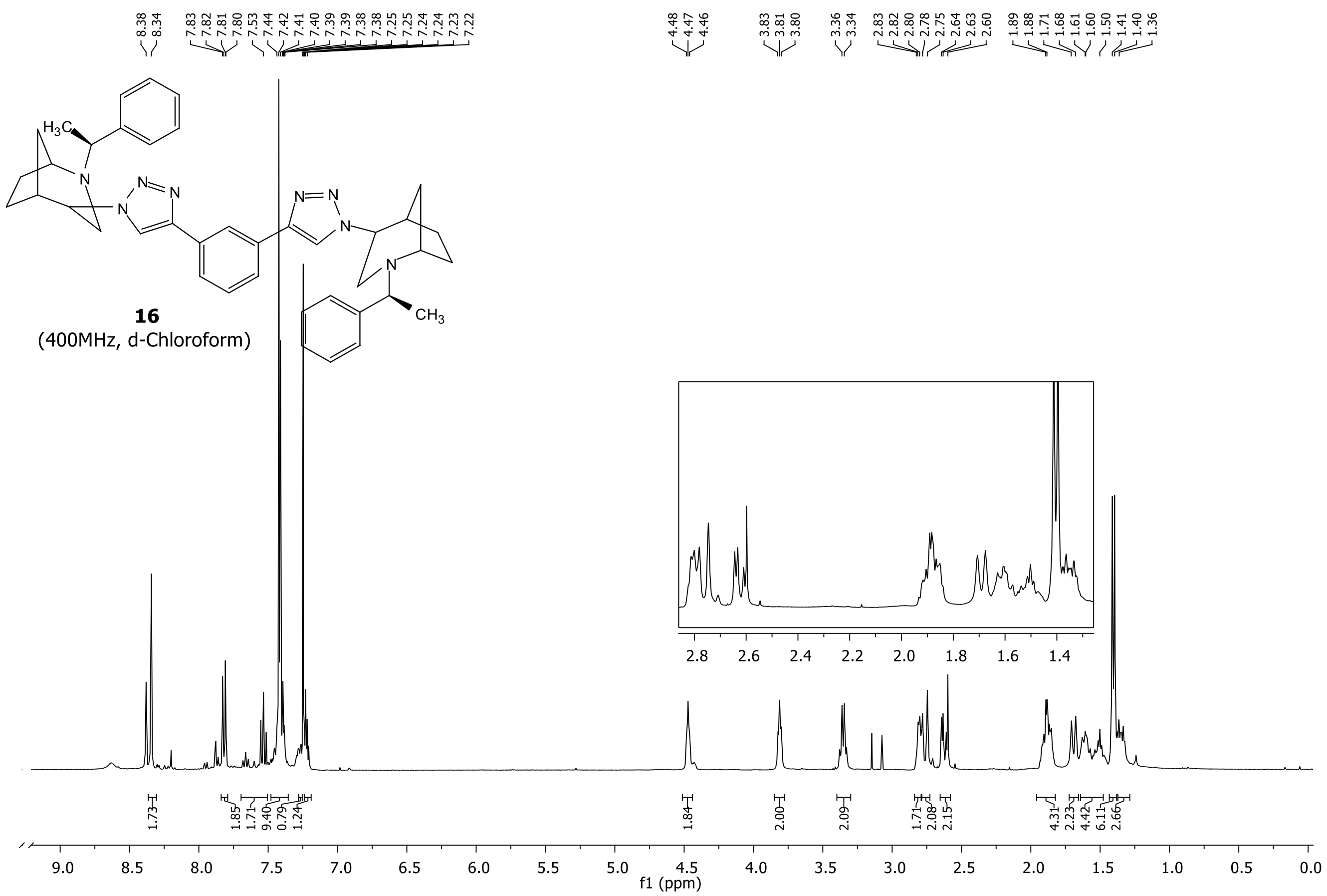
8.38
8.34
7.83
7.82
7.81
7.80
7.53
7.44
7.42
7.41
7.40
7.39
7.39
7.38
7.38
7.25
7.25
7.24
7.24
7.23
7.22
4.48
4.47
4.46
3.83
3.81
3.80
3.36
3.34
2.83
2.82
2.80
2.78
2.75
2.64
2.63
2.60
1.89
1.88
1.71
1.68
1.61
1.60
1.50
1.41
1.40
1.36
H_3C
N
N
N
N
N
N
N
N
N
CH_3
16
(400MHz, d-Chloroform)
1.73
1.85
1.71
9.40
0.79
1.24
1.84
2.00
2.09
1.71
2.08
2.15
4.31
2.23
4.42
6.11
2.66
f1 (ppm)

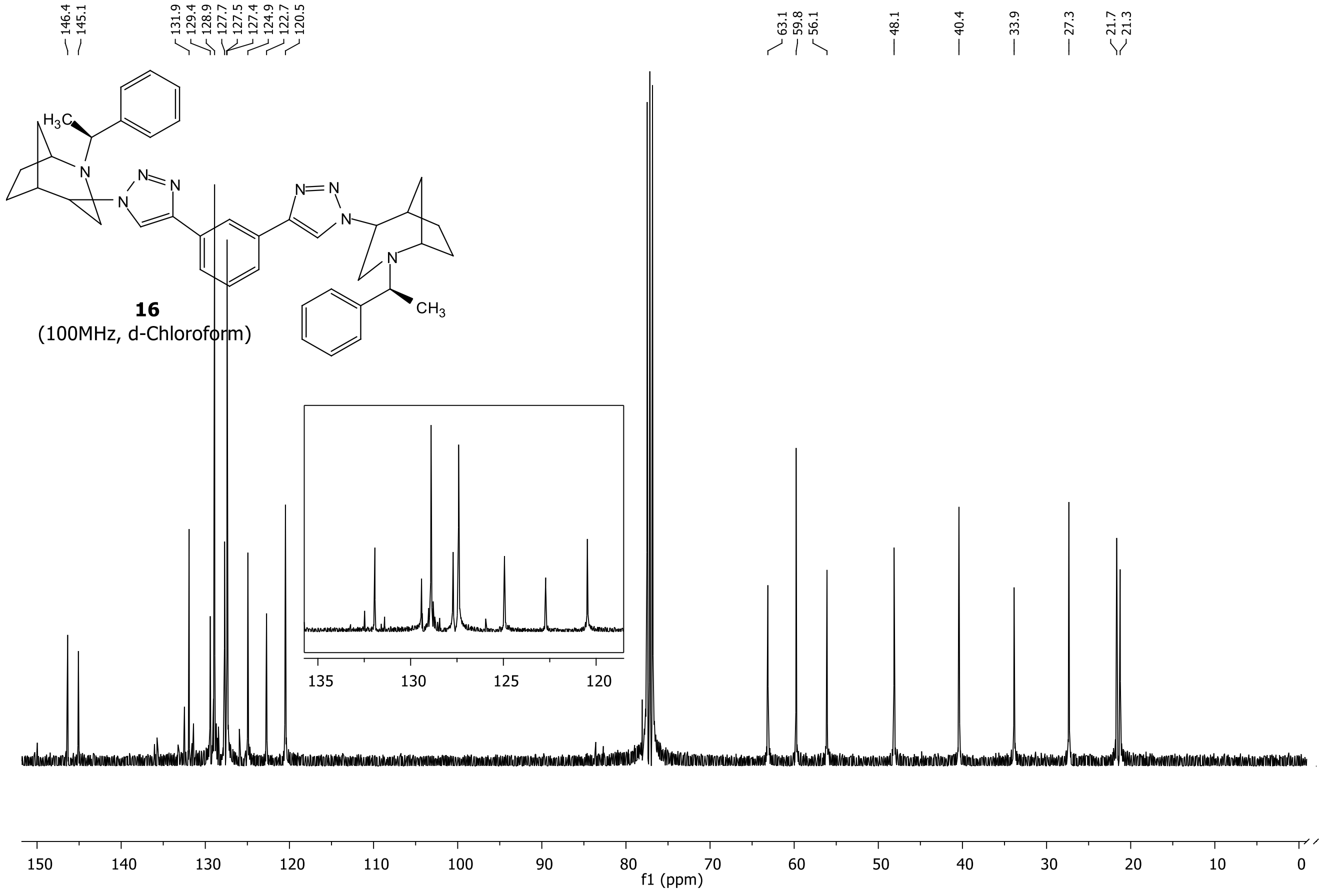
146.4
145.1
131.9
129.4
128.9
127.7
127.5
127.4
124.9
122.7
120.5
63.1
59.8
56.1
48.1
40.4
33.9
27.3
21.7
21.3
H_3C
N
N
N
N
N
N
N
N
N
CH_3
16
(100MHz, d-Chloroform)
f1 (ppm)

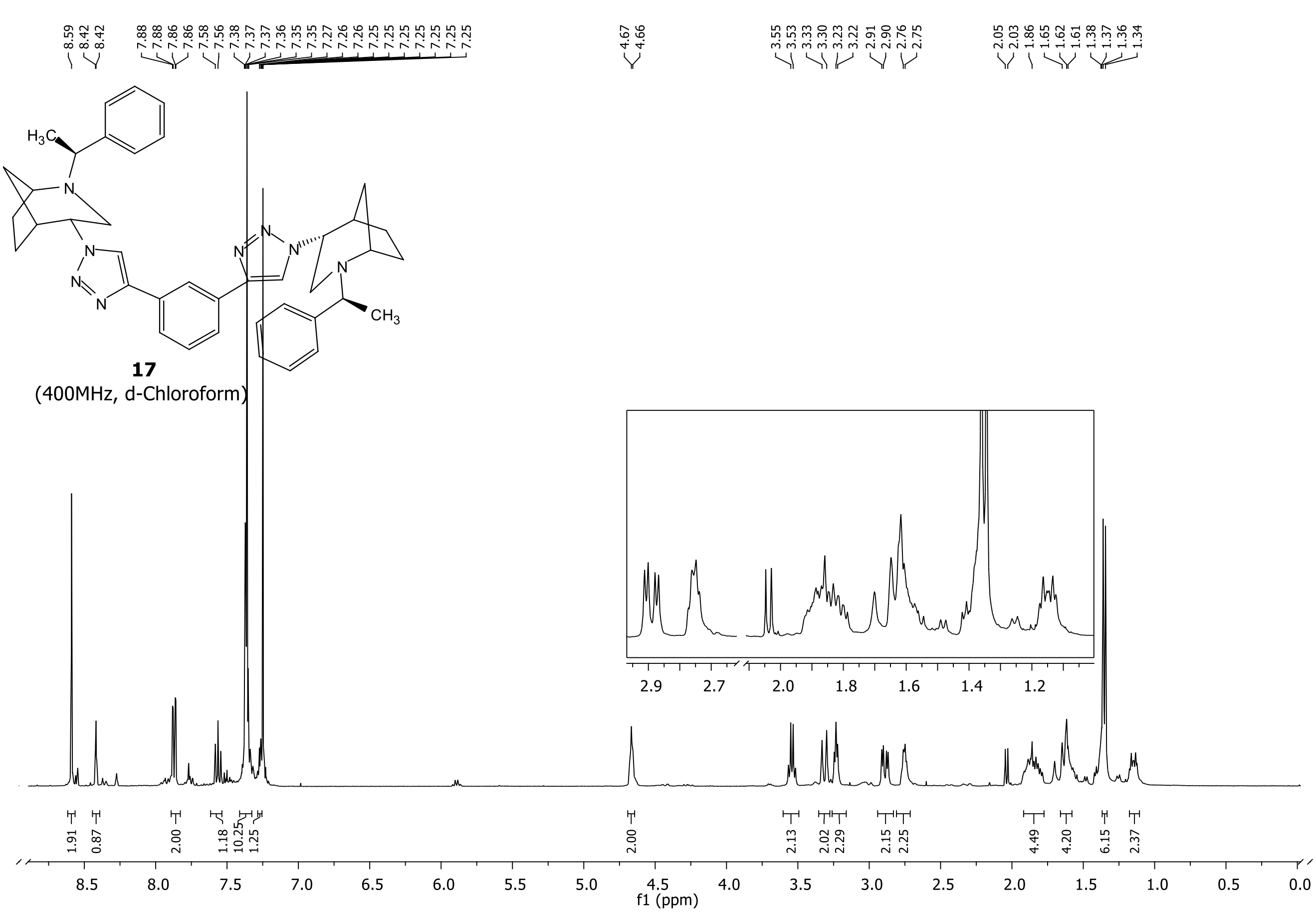
17
(400MHz, d-Chloroform)
f1 (ppm)

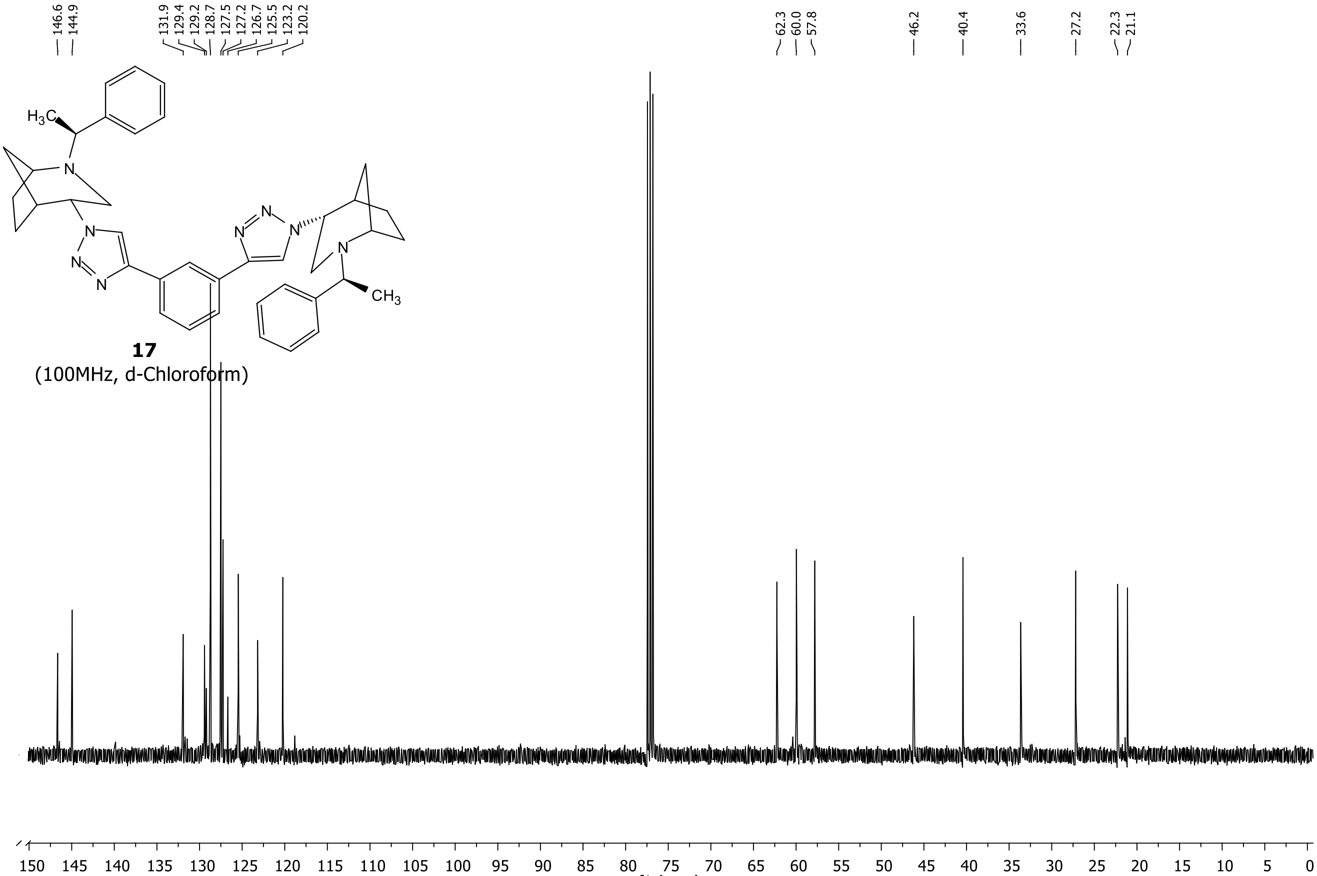
17
(100MHz, d-Chloroform)
f1 (ppm)

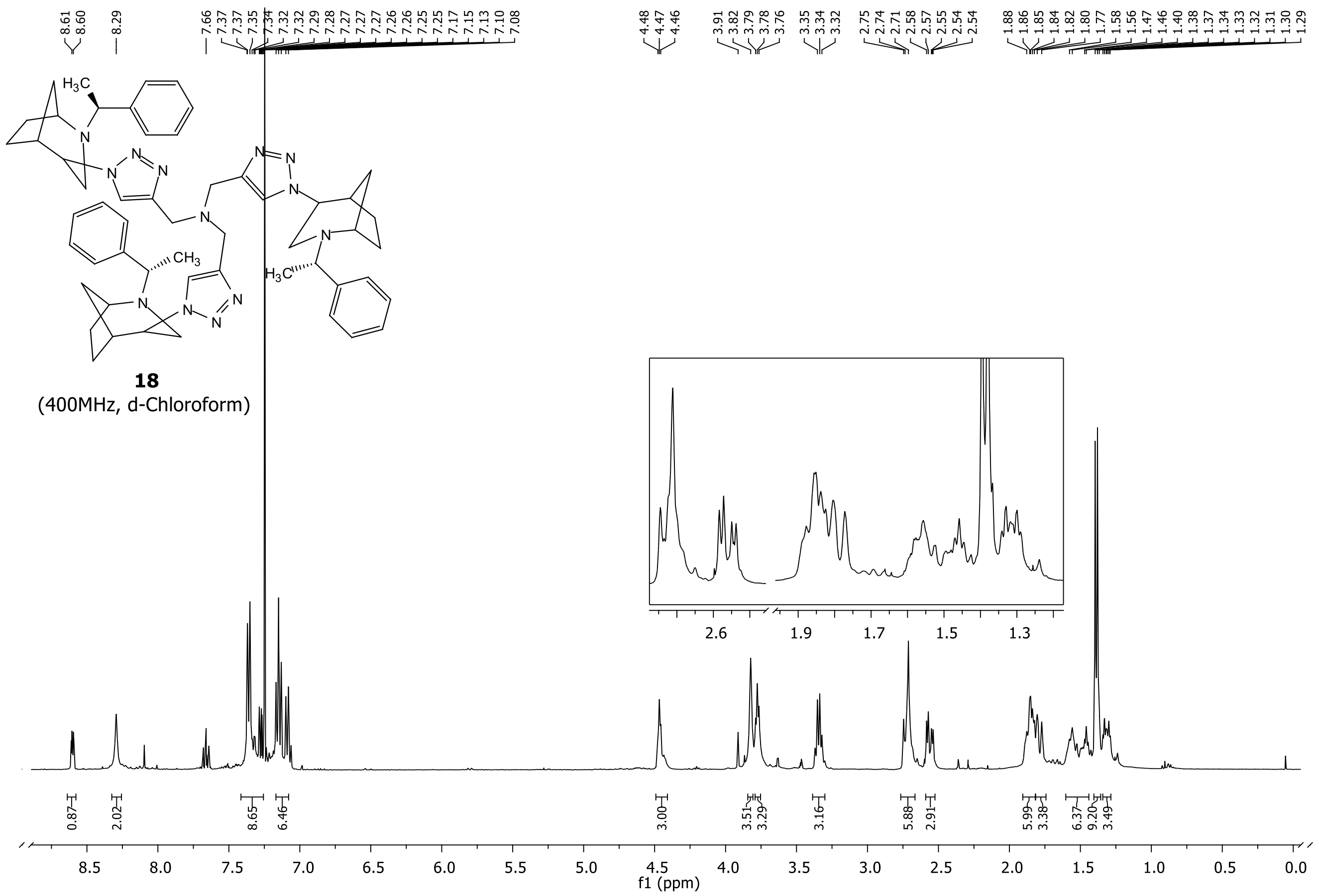
8.61
8.60
8.29
7.66
7.37
7.37
7.35
7.34
7.32
7.32
7.29
7.28
7.27
7.27
7.27
7.26
7.25
7.25
7.17
7.15
7.13
7.10
7.08
4.48
4.47
4.46
3.91
3.82
3.79
3.78
3.76
3.35
3.34
3.32
2.75
2.74
2.71
2.58
2.57
2.55
2.54
2.54
1.88
1.86
1.85
1.84
1.82
1.80
1.77
1.58
1.56
1.47
1.46
1.40
1.38
1.37
1.34
1.33
1.32
1.31
1.30
1.29
H_3C
N
N
CH_3
18
(400MHz, d-Chloroform)
f1 (ppm)

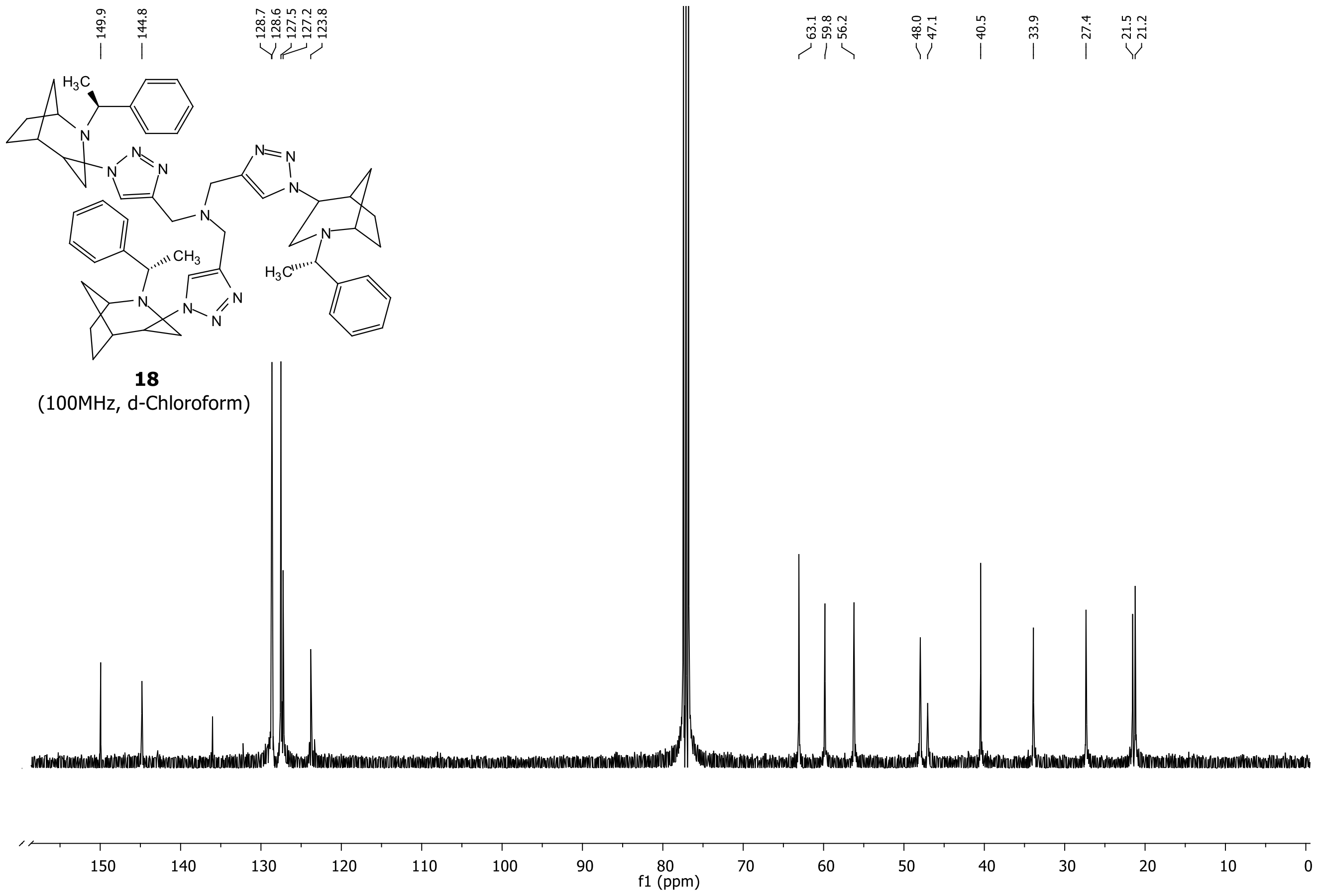
149.9
144.8
128.7
128.6
127.5
127.2
123.8
63.1
59.8
56.2
48.0
47.1
40.5
33.9
27.4
21.5
21.2
H_3C
N
N
CH_3
18
(100MHz, d-Chloroform)
f1 (ppm)

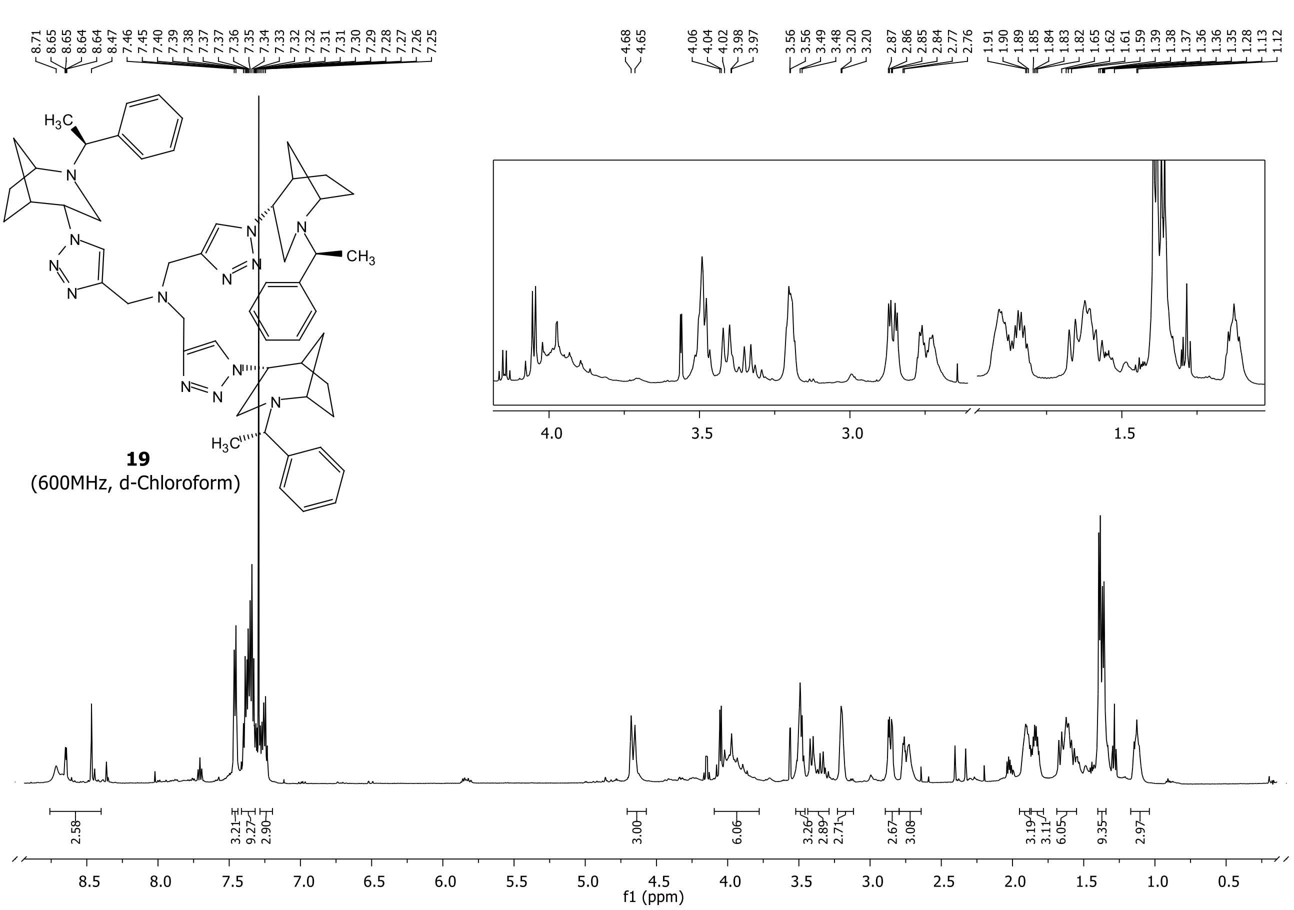
19
(600MHz, d-Chloroform)
H_3C
CH_3
f1 (ppm)

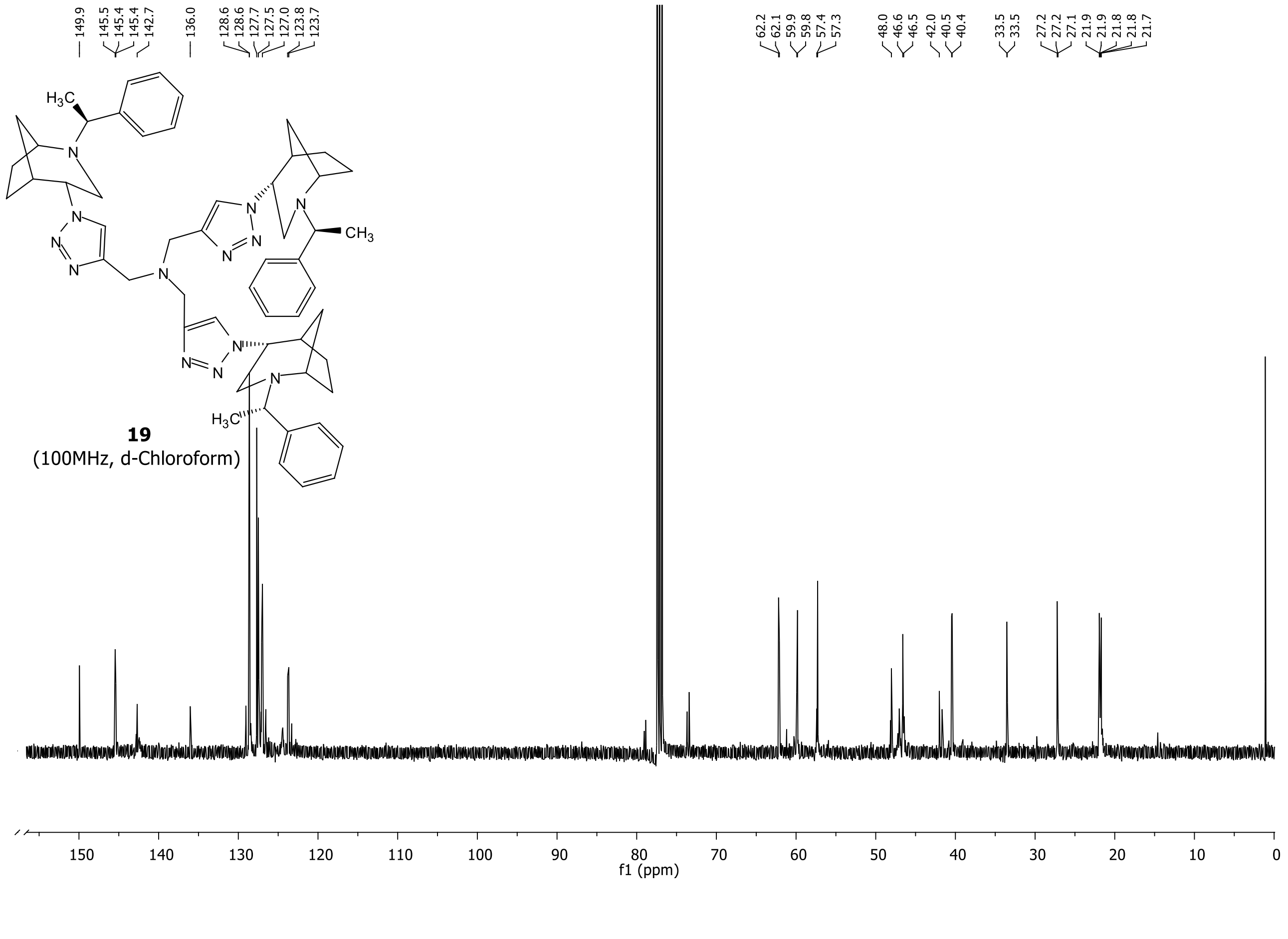
19
(100MHz, d-Chloroform)
H_3C
CH_3
f1 (ppm)

Raw IC_{50} data [μg/ml] and non-rounded data [μM]

Compound	IC_{50} [μg/ml]				IC_{50} [μM]				SI
	Hs294T	MIA PaCa-2	NCI-H1581	BALB3T3	Hs294T	MIA PaCa-2	NCI-H1581	BALB3T3	
Cisplatin	0.39±0.15	3.79±0.94	2.18±0.85	0.55±0.21	1.30±0.50	12.63±3.13	7.27±2.83	1.83±0.70	0.3
**1**	na[a]	na[a]	na[a]	na[a]	na[a]	na[a]	na[a]	na[a]	-
**2**	46.15±2.17	71.37±11.75	22.60±10.62	56.10±8.24	na[a]	na[a]	72.34±33.99	na[a]	-
**3**	27.57±2.93	31.11±1.39	3.51±0.39	29.41±0.59	88.53±9.41	99.90±4.46	11.27±1.25	94.44±1.89	8
**4**	3.49±0.92	32.15±0.61	2.90±0.44	0.64±0.28	9.52±2.51	87.73±1.66	7.91±1.20	1.75±0.76	0.2
**5**	26.68±3.56	31.63±0.13	6.88±1.63	10.46±8.31	72.8±9.71	86.31±0.35	18.77±4.45	28.54±22.68	1.5
**6**	27.84±5.58	31.53±0.39	11.49±1.31	28.79±0.41	74.54±14.94	84.42±1.04	30.76±3.51	77.08±1.10	2.5
**7**	24.60±3.10	28.74±0.74	2.76±0.43	19.68±2.30	65.87±8.30	76.95±1.98	7.39±1.15	52.69±6.16	7
**8**	29.71±4.07	31.85±0.20	6.23±0.05	24.74±1.32	73.81±10.11	79.12±0.5	15.48±0.12	61.46±3.28	4
**9**	28.53±2.36	30.78±1.76	3.43±0.56	20.14±1.31	70.88±5.86	76.47±4.37	8.52±1.39	50.03±3.25	6
**10**	6.26±2.05	24.31±0.97	2.54±0.22	7.17±3.04	10.35±3.39	40.19±1.60	4.20±0.36	11.85±5.03	3
**11**[b]	5.87±1.86	22.73±3.33	2.66±0.37	16.16±2.88	9.71±3.08	37.58±5.51	4.40±0.61	26.72±4.76	6
**12**	19.67±4.75	31.23±0.97	3.64±0.52	26.94±4.55	32.42±7.83	51.47±1.60	6.00±0.86	44.40±7.50	7
**13**	8.28±1.40	27.25±2.24	2.96±0.25	27.95±6.85	13.65±2.31	44.91±3.69	4.88±0.41	46.06±11.29	9
**14**[b]	49.36±6.07	na[a]	13.36±9.71	na[a]	77.26±9.50	na[a]	20.91±15.20	na[a]	-
**15**[b]	37.58±2.60	71.21±6.10	4.39±0.93	38.01±7.48	58.82±4.07	111.47±9.55	6.87±1.46	59.5±11.71	9
**16**[b]	na[a]	na[a]	34.45±3.24	na[a]	na[a]	na[a]	53.93±5.07	na[a]	-
**17**	na[a]	na[a]	na[a]	na[a]	na[a]	na[a]	na[a]	na[a]	-
**18**	18.75±4.52	23.11±0.23	2.20±0.06	20.78±2.45	20.83±5.02	25.67±0.26	2.44±0.07	23.08±2.72	9.5
**19**	26.22±1.22	28.66±2.08	2.94±0.38	24.89±2.92	29.13±1.36	31.84±2.31	3.27±0.42	27.65±3.24	8.5
**20**	27.46±6.57	30.35±3.51	3.01±0.66	18.98±2.34	57.01±13.64	63.01±7.29	6.25±1.37	39.40±4.86	6
**21**[b]	41.81±9.92	40.77±0.31	35.03±3.92	48.78±10.07	92.58±21.97	90.28±0.69	77.57±8.68	na[a]	-

[a] not active (IC_{50} > 100 μM) [b] poor or no solvability in DMSO, The IC_{50} value defined as the concentration of a compound which inhibited cell proliferation in 50%. The SI (Selectivity Index) was calculated for each compound using the formula: SI = IC_{50} for normal cell line BALB/3T3/ IC_{50} for NCI-H1581 human lung cancer cell line. The beneficial SI > 1.0 indicates a compound with efficacy against tumor cells greater than the toxicity against normal cells.
